# Use of temperature to improve West Nile virus forecasts

**DOI:** 10.1371/journal.pcbi.1006047

**Published:** 2018-03-09

**Authors:** Nicholas B. DeFelice, Zachary D. Schneider, Eliza Little, Christopher Barker, Kevin A. Caillouet, Scott R. Campbell, Dan Damian, Patrick Irwin, Herff M. P. Jones, John Townsend, Jeffrey Shaman

**Affiliations:** 1 Department of Environmental Health Sciences, Mailman School of Public Health, Columbia University, New York, New York, United States of America; 2 Department of Epidemiology, Mailman School of Public Health, Columbia University, New York, New York, United States of America; 3 Center for Vectorborne Diseases, University of California Davis, Davis, California; 4 St. Tammany Parish Mosquito Abatement District, St. Tammany Parish, Slidell, Louisiana, United States of America; 5 Arthropod-Borne Disease Laboratory, Suffolk County Department of Health Services, Yaphank, New York, United States of America; 6 Vector Control Division, Maricopa County Environmental Services Department, Phoenix, Arizona, United States of America; 7 Northwest Mosquito Abatement District, Wheeling, Illinois, United States of America; 8 Iberia Parish Mosquito Abatement District, Iberia Parish, New Iberia, Louisiana, United States of America; UNITED STATES

## Abstract

Ecological and laboratory studies have demonstrated that temperature modulates West Nile virus (WNV) transmission dynamics and spillover infection to humans. Here we explore whether inclusion of temperature forcing in a model depicting WNV transmission improves WNV forecast accuracy relative to a baseline model depicting WNV transmission without temperature forcing. Both models are optimized using a data assimilation method and two observed data streams: mosquito infection rates and reported human WNV cases. Each coupled model-inference framework is then used to generate retrospective ensemble forecasts of WNV for 110 outbreak years from among 12 geographically diverse United States counties. The temperature-forced model improves forecast accuracy for much of the outbreak season. From the end of July until the beginning of October, a timespan during which 70% of human cases are reported, the temperature-forced model generated forecasts of the total number of human cases over the next 3 weeks, total number of human cases over the season, the week with the highest percentage of infectious mosquitoes, and the peak percentage of infectious mosquitoes that on average increased absolute forecast accuracy 5%, 10%, 12%, and 6%, respectively, over the non-temperature forced baseline model. These results indicate that use of temperature forcing improves WNV forecast accuracy and provide further evidence that temperature influences rates of WNV transmission. The findings provide a foundation for implementation of a statistically rigorous system for real-time forecast of seasonal WNV outbreaks and their use as a quantitative decision support tool for public health officials and mosquito control programs.

## Author summary

West Nile virus (WNV) is the leading cause of domestically acquired arthropod-borne viral disease in the United States. Here we show that accurate retrospective forecasts of mosquito infection rates and human WNV cases can be generated for a variety of locations in the U.S. Incorporation of temperature forcing into a baseline dynamic model improves our ability to accurately forecast WNV outbreaks and provides further evidence that temperature modulates rates of WNV transmission. These findings provide a foundation for implementation of a statistically rigorous system for real-time short-term and seasonal forecast of WNV. Such a decision support tool would help public health officials and mosquito control programs target control of infectious mosquito populations, alert the public to future periods of elevated WNV spillover transmission risk, and identify when to intensify blood donor screening.

## Introduction

West Nile virus (family *Flaviviridae*, genus *Flavivirus*, WNV) is the leading cause of domestically acquired arthropod-borne viral (arboviral) disease in the United States (U.S.) [1]. It was first identified in North America in New York City during the summer of 1999 [2] and by 2003 had spread throughout the continent [3]. Following this expansion, the virus did not disappear but instead settled into a pattern of endemic zoonotic transmission between vector mosquitoes and avian hosts. Spillover transmission to humans has since continued; however, during 2012 human WNV cases surged to numbers not seen since 2003 suggesting that it will continue to produce unpredictable local and regional outbreaks throughout the U.S. [3].

Vector control agencies monitor mosquito and viral activity and use this information to guide mosquito and WNV control measures [3, 4]; however, due to the nonlinearity of WNV transmission dynamics [5] it is non-trivial to project future WNV activity from current, observed conditions. Indeed, seasonal WNV outbreaks vary considerably in size and scope [3, 6] such that, even after an outbreak has begun, it remains difficult to predict the future characteristics of the epidemic curve [6]. Accurate forecasts of WNV outbreak characteristics are thus needed so that public health response and mosquito control efforts can be more effectively coordinated and implemented [7].

In a recent study, we showed that accurate and reliable predictions of seasonal WNV outbreaks can be made using a parsimonious mathematical model representing the transmission dynamics of WNV among mosquitoes and birds, as well as spillover to humans [8]. The dynamic model was recursively optimized using the ensemble adjustment Kalman filter (EAKF), a data assimilation technique, and observational estimates of the proportion of infectious mosquitoes and the number of reported human cases. This model system accurately forecast mosquito infection rates prior to the week of mosquito peak infection, and accurately predicted the seasonal total number of human WNV cases up to 9 weeks prior to the last reported case. The model-inference system thus provides a basic, parsimonious structure for forecasting WNV; however, it is natural to wonder whether inclusion of additional physical environmental factors within the model structure, which have been shown to affect WNV transmission dynamics, would improve forecast skill [8].

Ecological and laboratory studies have demonstrated that physical environmental factors (e.g., temperature, precipitation, hydrology, and humidity [9–26]) influence WNV transmission dynamics and spillover infection of humans, often in complex, nonlinear fashion. For example, rainfall increases near-surface humidity, which enhances mosquito flight activity and host-seeking behavior and alters the abundance and type of aquatic habitats available to the mosquito for egg deposition and sub-adult growth [16]. Thus, for some species, above average rainfall can increase vector populations, but extreme rainfall events can decrease survival by flushing larvae from aquatic habitats [17]. On the other hand, years with below average rainfall may concentrate water resources for both avian hosts and mosquitoes creating optimal conditions for WNV amplification [18]. Additionally, warmer temperatures affect mosquito development rates, shorten the duration of the gonotrophic period [12], and decrease the extrinsic incubation period of the virus [9–11]; however, at high temperatures, vector survival decreases [13, 14]. These myriad non-linear effects make it difficult to estimate how environmental variables influence overall WNV risk.

**Fig 1:**
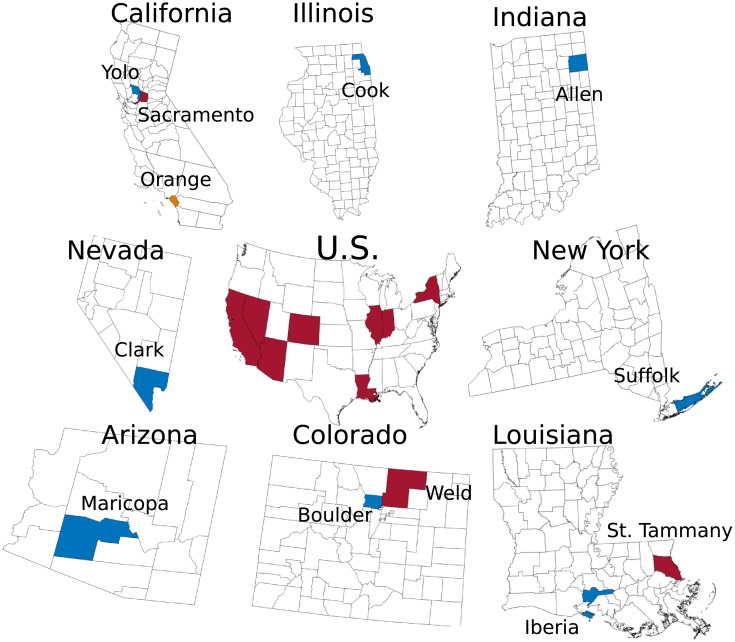
Twelve counties in 8 different states (red) were evaluated: Yolo County CA (blue), Sacramento County CA (red), Orange County CA (Orange), Clark County NV (blue), Maricopa County AZ (blue), Boulder County CO (blue), Weld County CO (red), Iberia Parish LA (blue), St Tammany Parish LA (red), Suffolk County NY (blue), Cook County IL (blue), and Allen County IN (blue).

**Fig 2:**
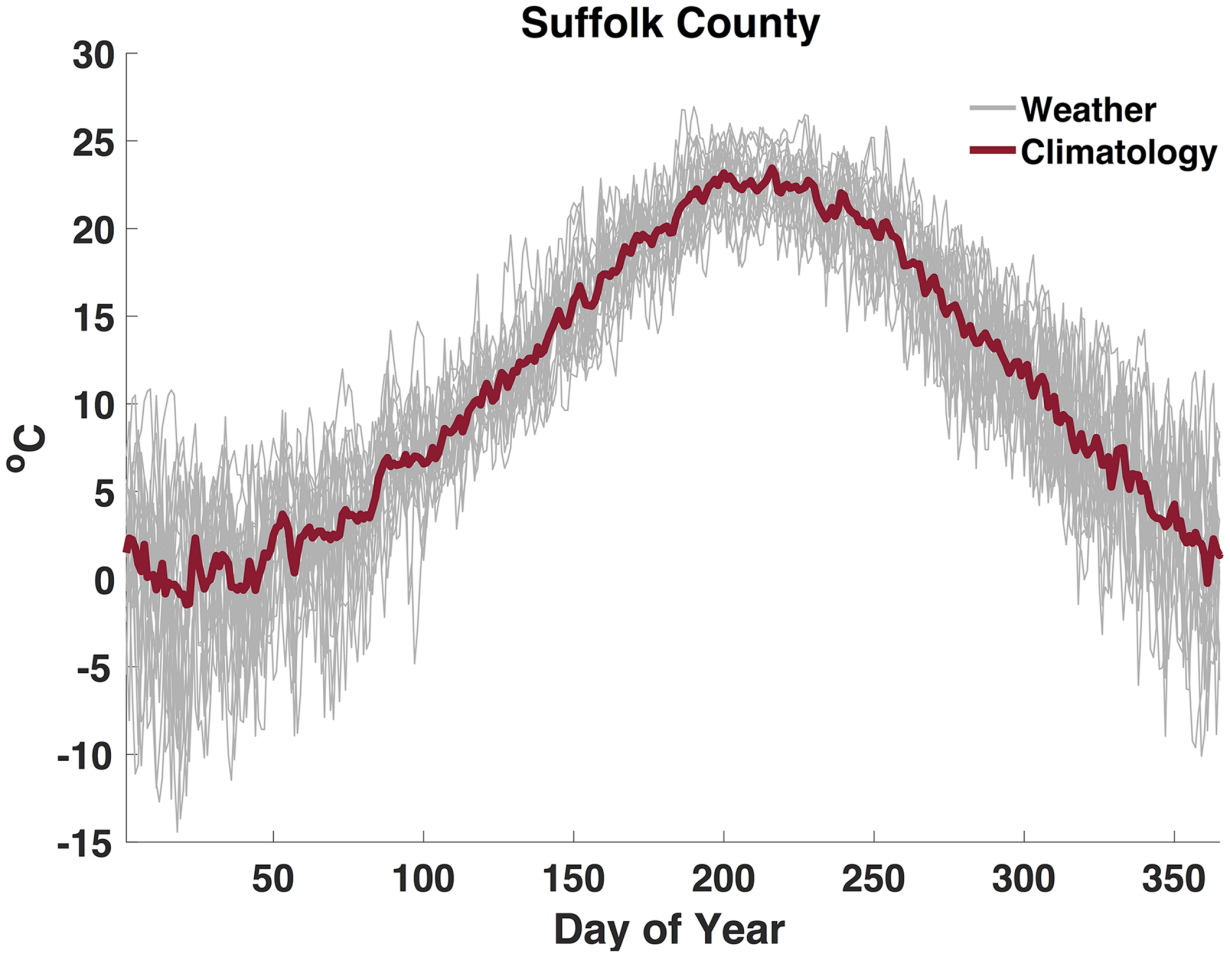
Shows daily weather from 1981 to 2000 and climatology over that period for Suffolk County. Grey represents daily temperature, and red is the temperature climatology, i.e. the average of daily weather observations.

Here, we explore whether the addition of a biological parameter depicting the relationship between temperature and the extrinsic incubation period will improve our ability to forecast WNV. The challenge is that the inclusion of too many processes results in a high-dimensional model structure, which, given the limited observational data streams available, may be difficult to optimize. We use the relationship between temperature and the extrinsic incubation period to expand our previously developed parsimonious model to include an environmental factor, a temperature-forcing parameter, that modulates the zoonotic transmission of WNV between mosquito vectors and avian hosts. We couple this mechanistic model with the ensemble adjustment Kalman filter (EAKF) [27] for data assimilation and generate retrospective forecasts for a total of 110 outbreak years in 12 different U.S. counties (Fig 1). The accuracy of the forecasts that use historical climatology for temperature forcing, see Fig 2 for example, are compared with WNV forecasts generated with our baseline system, which lacks temperature forcing and has one less parameter to optimize [8]. We show that temperature forcing improves forecast accuracy for total human case numbers, the week of peak infectious mosquito abundance, and the magnitude of that abundance for much of the WNV season, whereas the total number of infectious mosquitoes is more accurately predicted using the baseline forecast model.

## Results

### Retrospective forecasts

**Fig 3:**
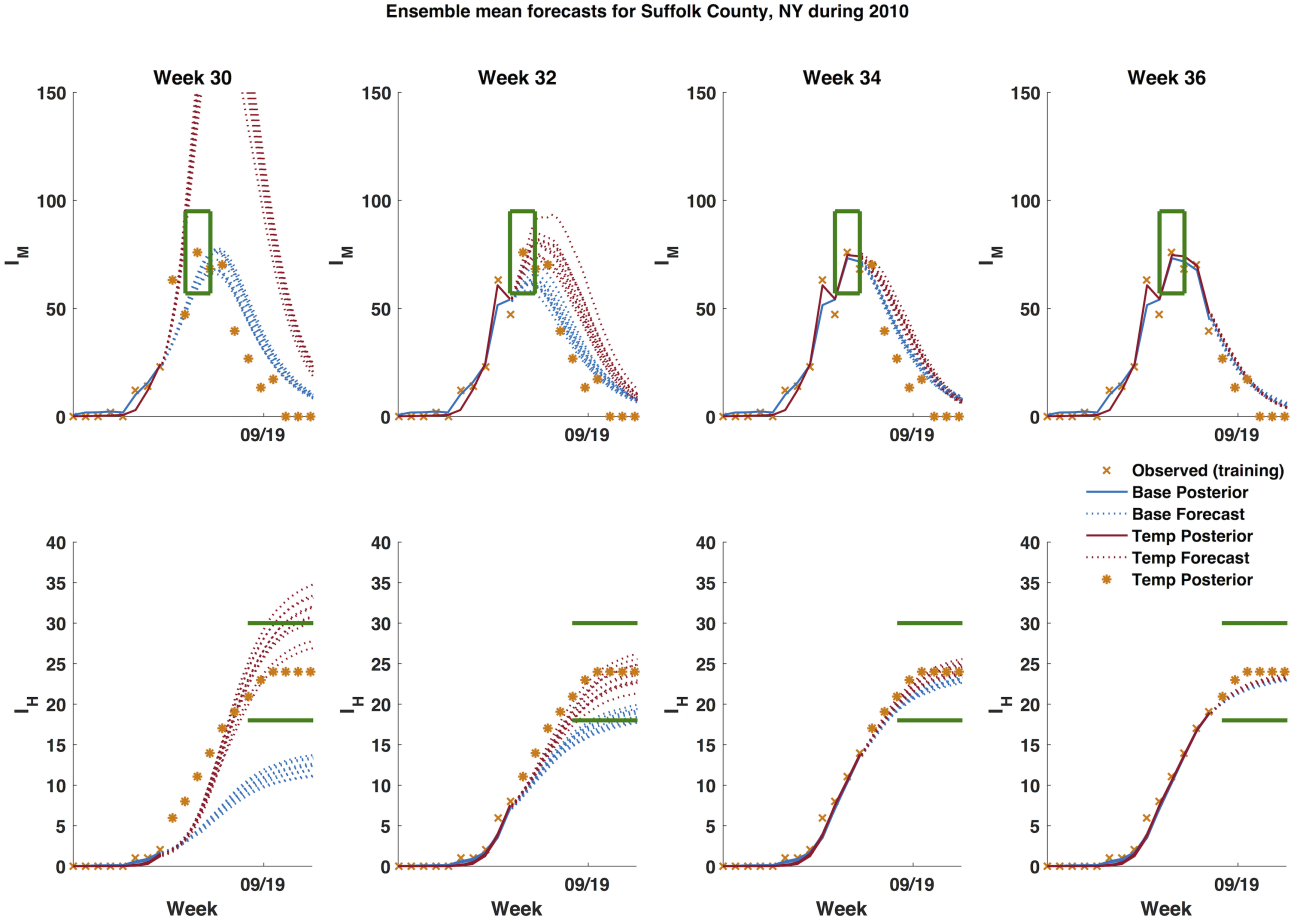
Example forecasts of infectious mosquitoes and human WNV cases for Suffolk County, NY during 2010. Blue represents the baseline model and red represents the temperature-forced model. The dotted lines are the ensemble mean forecasts and solid lines are the ensemble mean posterior distribution, orange *x*’s are data points assimilated into the model, orange * are future observations, and the green lines represent the target range of an accurate forecast. A forecast was deemed accurate if: 1) peak timing was within ±1 week of the observed peak of infectious mosquitoes; 2) peak infection rate was within ±25% of the observed peak infection rate; and 3) human WNV cases were within ±25% of the total number of reported cases.

Retrospective WNV predictions were generated for 12 different counties representing a total of 110 outbreak years (Fig 1, see Methods). For each annual outbreak, the model-EAKF system was initiated four weeks prior to the first positive mosquito observation. Each week, observations of human WNV cases and, when more than 300 mosquitoes were sampled, mosquito infection rates were assimilated using the EAKF, and a forecast was generated by integrating the posterior model ensemble to the end of the outbreak season. Forecasts were generated each week from the first detection of infectious mosquitoes to the end of the year using both the baseline system [8], which lacks temperature forcing, and the temperature-forced system (for example forecasts see Fig 3 and supplementary information (SI) S1 and S2 Figs).

Forecast accuracy was evaluated for both the short-term, 1–4 weeks in the future, and the season. Short-term forecast were deemed accurate if the ensemble mean trajectory was within ±25% or ±1 case, whichever was larger, of the number of human cases reported for each of the next 4 weeks. Seasonal forecast accuracy was assessed using 4 metrics: total human WNV cases, total infectious mosquitoes, peak infectious mosquitoes and peak timing. Forecasts were deemed accurate if the ensemble mean trajectory was within ±25% or ±1 case, whichever was larger, of the first metric, within ±25% of the next two metrics, and within ±1 week of the fourth metric. Forecast accuracy across all outbreaks, regions and seasons, was assessed for both the baseline model and temperature-forced model as a function of calendar week. Forecasts were further grouped by prediction lead-time, here defined as the week of forecast generation minus the week of predicted peak mosquito infection.

Both the baseline model and temperature-forced model produced accurate short-term forecasts (see S3 and S4 Figs). For weeks 31–40, when 70% of human cases were reported (see S5 Fig), the temperature-forced model forecasts accurately predicted human case numbers 1, 2, 3, and 4 weeks in advance 74, 63, 57 and 52% of the time, respectively. Without temperature forcing, the baseline model was accurate 1, 2, 3, and 4 weeks in advance 73, 60, 52, and 48% of the time, respectively. For forecasts with predicted leads between -1 to 4 weeks past the peak week of mosquito infection, when 52% of human cases were reported, the temperature-forced model on average improved absolute forecast accuracy for human case numbers 1, 2, 3, and 4 weeks ahead by 6, 8, 10 and 12%, respectively, over the baseline model.

**Fig 4:**
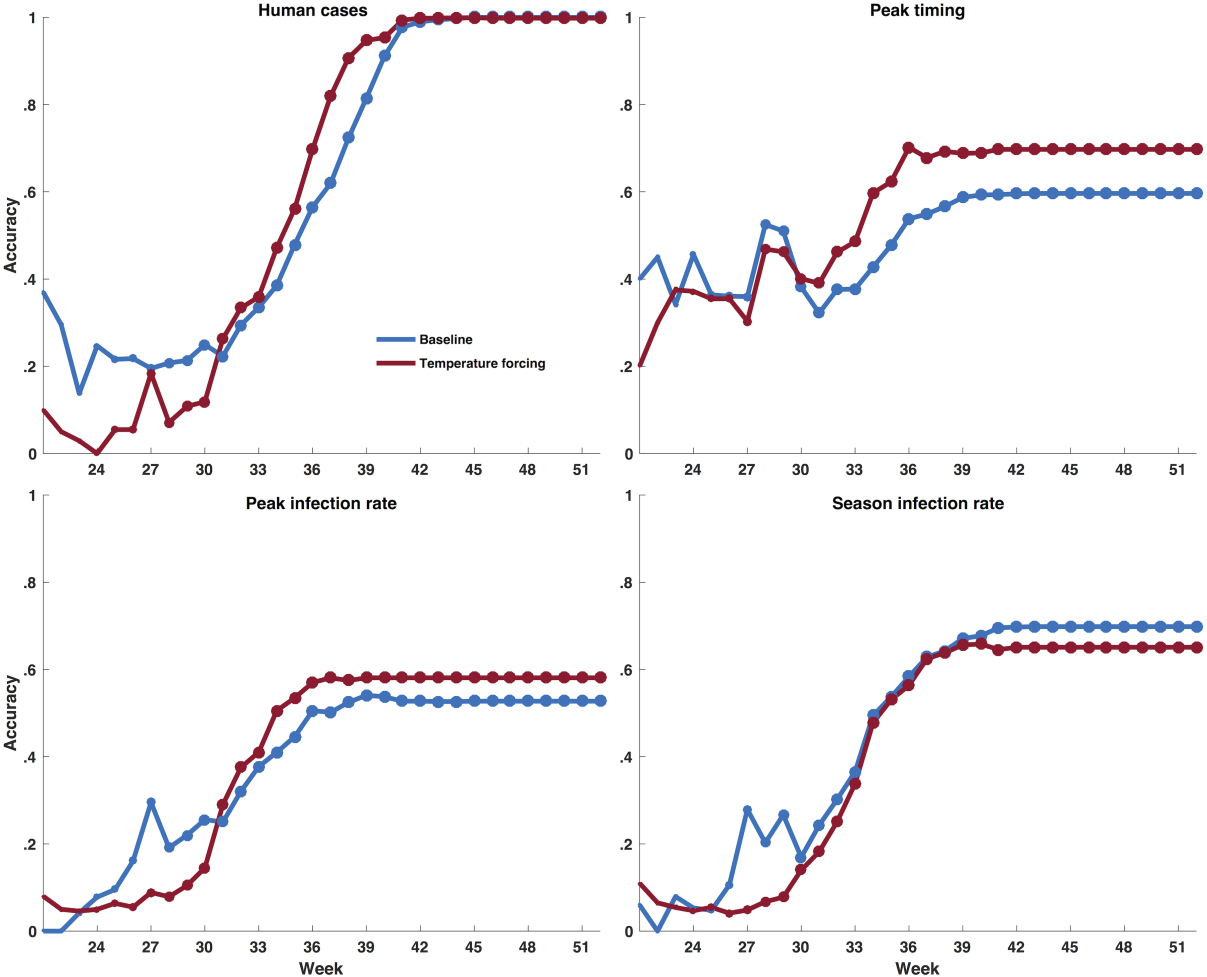
The fraction of forecasts accurate for both the temperature-forced model (red) and baseline model (blue) as a function of week of the year for the metrics human WNV cases, peak timing (week of peak mosquito infection rates), peak infection rate, and total infectious mosquitoes. A forecast was deemed accurate if: 1) peak timing was within ±1 week of the observed peak of infectious mosquitoes; 2) peak infection rate was within ±25% of the observed peak infection rate; 3) total infectious mosquitoes were within ±25% of the observed; and 4) human WNV cases were within ±25% or ±1 case of the total number of reported cases, whichever was larger.

For the seasonal forecasts, the baseline model generated more accurate forecasts as a function of calendar week for total number of human cases, peak timing, and peak magnitude early in the season but by the middle of the season (the end of July), weeks 31, 30, and 31, for these 3 metrics, respectively, the temperature-forced model forecasts were more accurate (Fig 4). Between week 31 and week 40, when 70% of human cases were reported, the temperature-forced model forecasts on average improved absolute accuracy by 10, 12, and 6% for the total number of human cases, peak timing of infectious mosquitoes, and peak magnitude, respectively, over the baseline model forecasts. Only one-fifth of outbreaks among mosquitoes peaked prior to week 31, and only one-quarter of human cases were reported prior to week 31 (see S5 Fig); thus, for the majority of outbreaks the temperature-forced model produced more accurate forecasts of peak timing, total number of human cases, and the peak mosquito infection rates. On the other hand, the baseline model was generally more accurate forecasting the total number of infectious mosquitoes recorded during an outbreak.

**Fig 5:**
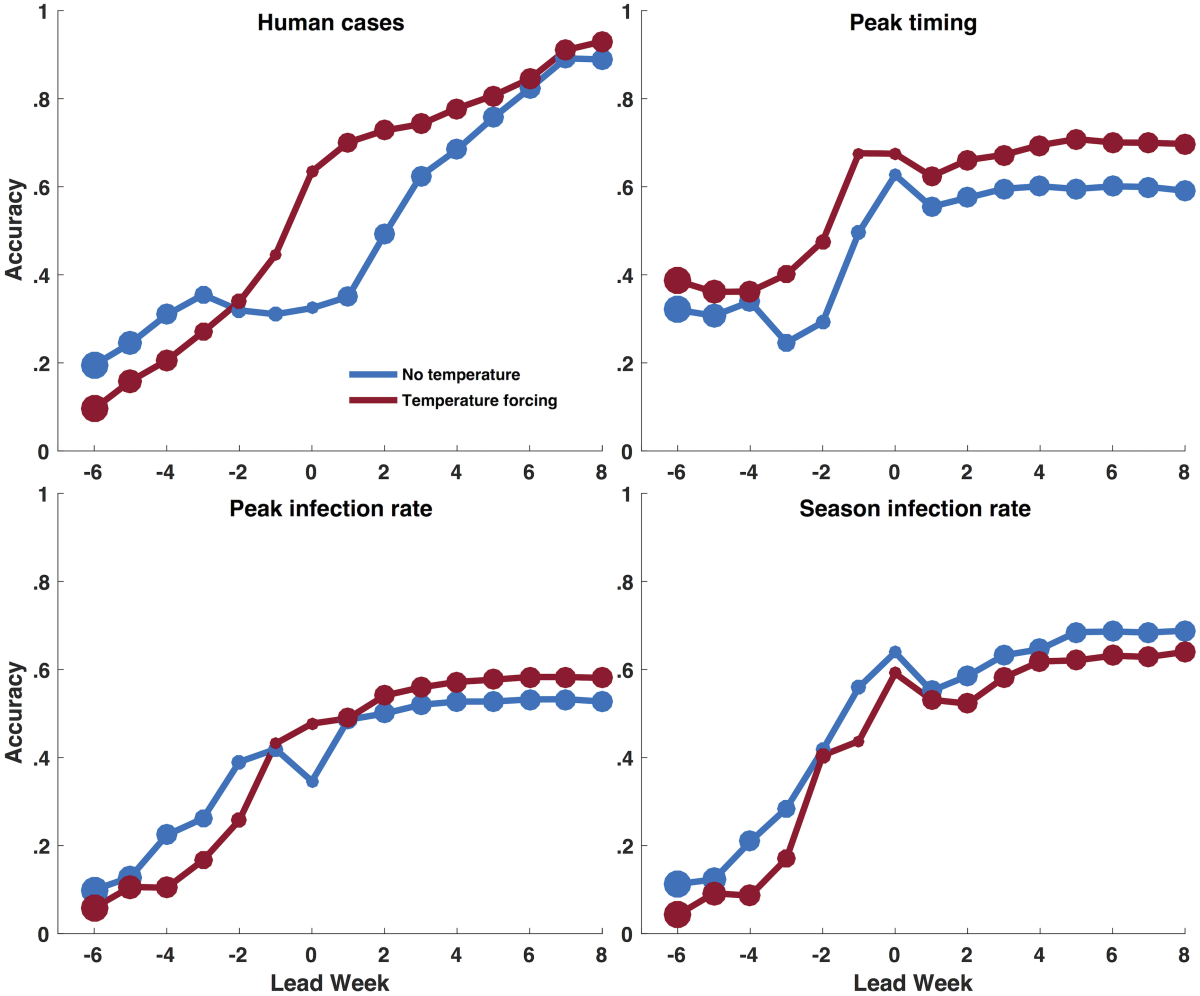
The fraction of forecasts accurate for both the temperature-forced model (red) and baseline model (blue) as a function of lead week for the metrics human WNV cases, peak timing (week of peak mosquito infection rates), peak infection rate, and total infectious mosquitoes. A forecast was deemed accurate if: 1) peak timing was within ±1 week of the observed peak of infectious mosquitoes; 2) peak infection rate was within ±25% of the observed peak infection rate; 3) total infectious mosquitoes were within ±25% of the observed; and 4) human WNV cases were within ±25% or ±1 case of the total number of reported cases, whichever was larger. Note that for all metrics lead week is shown with respect to the week of peak mosquito infection. The size of the dot at each lead week represents the number of forecast generated for that lead week; larger dots indicate more forecasts were generated with a particular predicted lead.

Prior to the predicted peak, the baseline system forecast the number of human cases, the peak mosquito infection rate, and the seasonal mosquito infection rate more accurately than the temperature-forced system (Fig 5); however, peak timing was more accurately forecast using the temperature-forced system at all leads. Though the temperature-forced model was less accurate predicting seasonal mosquito infection rates, it generated more accurate forecasts of spillover transmission to humans. At 0, 1 and 2 weeks past the predicted week of peak mosquito infection, the temperature-forced model forecasts of total human WNV cases were accurate 63, 70 and 73% of the time, respectively, whereas the baseline model was accurate 32, 35 and 49%, of the time, respectively. Only one-quarter of human cases were reported before the week of peak mosquito infection. Consequently, forecasts of human cases near the predicted peak of mosquito infection are prior to reporting of the majority of human cases and thus provide considerable advanced warning (see S5 Fig).

Seasonal forecast accuracy was also compared to local historical average outbreak conditions to determine if the system could simply forecast accurately whether an outbreak was earlier or later than normal, or larger or smaller than normal. The average outbreak for each county was defined as the mean value for the 4 metrics (total human WNV cases, total infectious mosquitoes, peak infectious mosquitoes and peak timing) for all years excluding the forecast year. Both forecasting approaches were greater than 65% accurate predicting all 4 metrics 3 weeks prior to the predicted week of peak mosquito infection, see S6 Fig.

**Fig 6:**
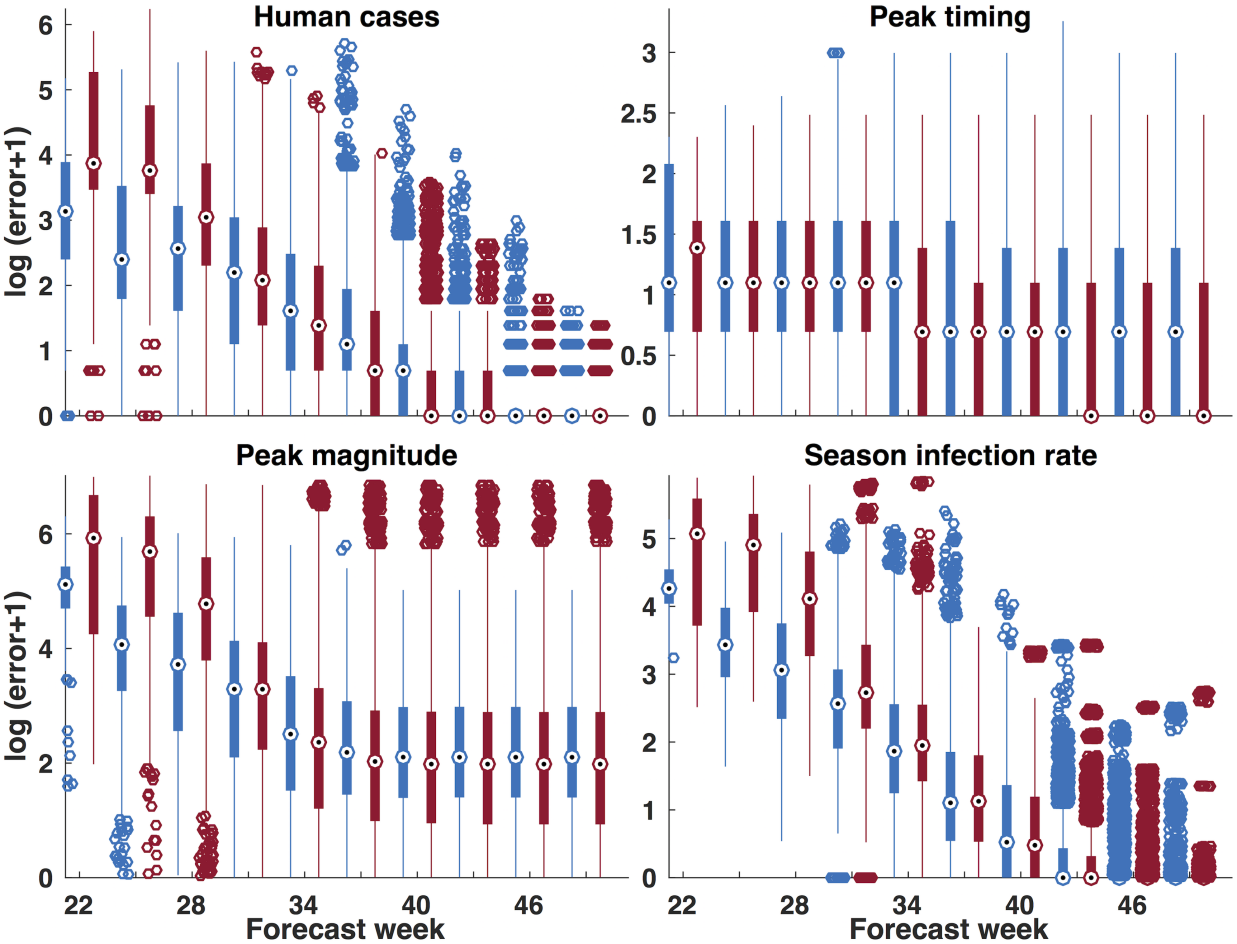
Log (error +1) of forecasts generated by the baseline model, blue, and the temperature-forced model, red. Absolute error was calculated and compared for each prediction of observed peak of infectious mosquitoes, maximum mosquito infection rate, and the total number of human cases over the entire season, while the root mean squared error (RMSE) was calculated for predictions of the total number of mosquitoes observed over the season.

Fig 6 shows absolute error for the 4 forecast metrics in which the predictions have been binned in 3-week groupings. We used the Wilcoxon signed-rank test to evaluate which forecasts had significantly lower error for a given week (see S1 Table). The baseline model had significantly lower error than the temperature-forced model for human cases, peak magnitude and total infectious mosquitoes prior to weeks 31, 31 and 35, respectively. However, during the part of the season when the majority of spillover transmission occurs, the temperature forced model had significantly lower error than the baseline model for human cases, peak magnitude and remaining weekly seasonal infectious mosquitoes for weeks 31 to 47, 34 to 52 and 35 to 50, respectively. From week 31 until the end of the outbreak the temperature-forced model had statistically significant lower error than the baseline model in its forecasts of mosquito infection peak timing.

The short-term forecast accuracy for human cases was also evaluated using the Wilcoxon signed-rank test (see S2 Table). The temperature-forced forecast had statistically significant lower error for the remainder of the season beginning week 33, 32, 31 and 31 for the 1, 2, 3 and 4 week forecasts, respectively.

We further evaluated differences in forecast accuracy based on geographic location (northern v. southern) and precipitation levels (wetter v. drier) (see S1 Text for details). For 3 of the 4 sub-groups we found similar results in which the temperature-forced model improved retrospective forecast accuracy for 3 of the 4 metrics evaluated relative to the baseline model (see S7–S10 Figs). Using the temperature-forced model, counties experiencing more precipitation were more accurately forecast than drier counties (see S11 Fig), whereas there were no consistent differences between northern versus southern counties (see S12 Fig).

**Fig 7:**
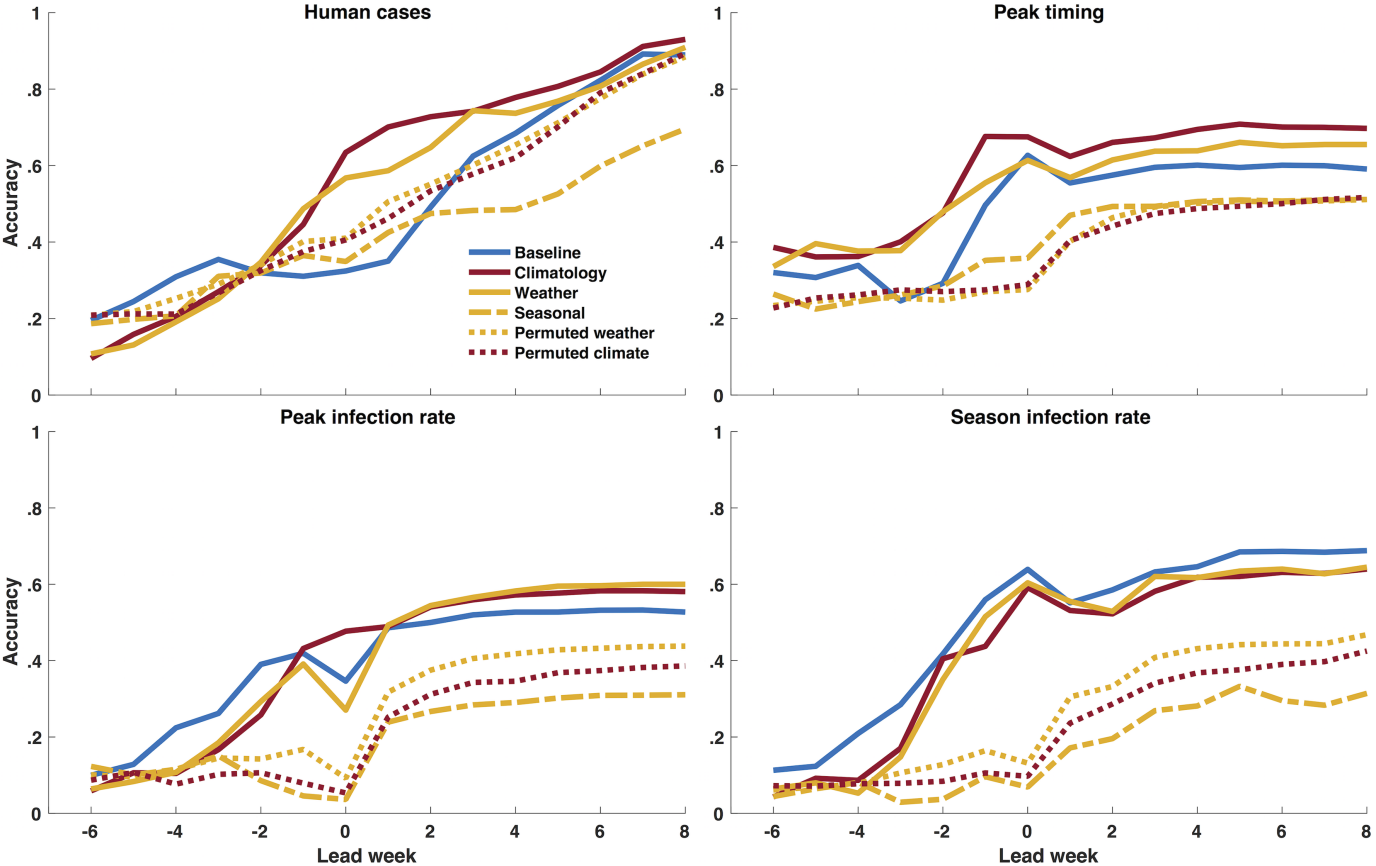
Comparison of forecast accuracy as a function of calendar week using different temperature forcings for the metrics human WNV cases, peak timing (week of peak mosquito infection rates), peak infection rate, and total infectious mosquitoes. Red represents climatology and yellow represents observed local temperature—daily climatology (red line), observed local daily temperature (yellow line), observed seasonal average temperature (yellow dashed), permuted observed local daily temperature (yellow dot) and permuted climatology (red dot). A forecast was deemed accurate if: 1) peak timing was within ±1 week of the observed peak of infectious mosquitoes; 2) peak infection rate was within ±25% of the observed peak infection rate; 3) total infectious mosquitoes were within ±25% of the observed; and 4) human WNV cases were within ±25% or ±1 case of the total number of reported cases, whichever was larger.

We additionally simulated and compared 6 different temperature-forcing structures in order to better understand the impact of temperature on WNV disease dynamics (see Methods). The 6 scenarios are: 1) the baseline model without temperature forcing, 2) our principal temperature forcing: daily climatology temperature forcing, 3) observed daily temperature forcing (i.e., real weather, which is not feasible for use in real time forecasting as such data would not be available), 4) observed seasonal average temperature forcing, 5) permuted observed temperature values for a given year and location; and 6) permuted temperature values from the historical 1981–2000 record (see S13 Fig for examples of each temperature time series). The climatology and real weather-forced models both performed better than the baseline model (see Fig 7 and S14 Fig). Real weather forcing was more accurate than the baseline model for peak timing, peak magnitude and total human WNV cases during the majority of the outbreak season (S3 Table). However, forecast accuracy was best with climatology temperature forcing (S4 Table), indicating that a smoothed temperature forcing function generates more accurate forecasts. The seasonal average and permuted temperature forcings degraded forecast accuracy, indicating that a seasonal cycle and realistic weather patterns are needed to improve forecast accuracy over the baseline model.

## Discussion

Our findings demonstrate that a simple WNV model, iteratively optimized with data assimilation methods and weekly observations of mosquito infection rates and human WNV cases, can produce accurate forecasts of mosquito infection rates, infectious mosquito biting pressure and human cases in a variety of locations around the U.S. (Fig 1). Though WNV transmission dynamics vary by location [10, 28–32], the simple forecasting framework presented here was designed for broad application, and, in representing modulation of the extrinsic incubation period, temperature forcing improves forecast skill over the non-temperature forced model for much of the WNV season.

Specifically, the temperature-forced forecast model improves prediction accuracy for 4 of the 5 metrics evaluated: short-term (1–4 week) human cases, the total number of human cases, peak timing of infectious mosquitoes, and peak magnitude of infectious mosquitoes. These improvements manifest prior to when the majority of human cases are reported and prior to the peak week of mosquito infectiousness. Also, the results were not sensitive to the tolerance (e.g. ±25%) used to define accuracy (see S15 Fig).

The forecasts of the total number of infectious mosquitoes on average were more accurately predicted using the baseline model. When broken down by calendar week it is apparent the baseline model is more accurate forecasting total infectious mosquitoes early in the season, but around week 30, when mosquito infection rates have risen, the temperature-forced model is more accurate (see S16 Fig).

Estimated mosquito-bird contact rates differed between the baseline model and the temperature-forced model (S17 Fig). This discrepancy is not surprising given the imposed differences in the model forms (see Eqs 6 and 7 in the Methods). In contrast estimates of the probability of spillover from mosquitoes to humans were more aligned for the two models (see S18 Fig). For both mosquito-bird contact and spillover average parameter values varied between counties, and counties with greater population density tended to have higher spillover rates. For the majority of counties spillover increases later in the season, and this increase occurs around the time when mosquito-bird contact rates decreases. These shifts represent changes in mosquito feeding preference from avian to mammalian hosts [33–35]. For two counties, Allen County and Iberia Parish, we did not see an increase in human spillover due to the majority of years having few (0 or 1) human WNV cases reported. The other counties had higher numbers of human cases.

In expanding the WNV forecast system, we had to choose which physical environmental effects to incorporate. While precipitation, hydrology, humidity and temperature all influence WNV dynamics [9–26], inclusion of all these factors would increase the dimension of the model structure, which, given the limited observational data streams available, might make optimization more difficult. On the other hand, the model needs to contain sufficient dynamics to generate a characteristic WNV outbreak in free simulation. Addition of temperature-forcing appears to provide additional realistic biological constraint of WNV transmission dynamics and allows more accurate forecast during the heart of WNV outbreaks.

In addition to affecting viral development, temperature forcing may also impact host seeking behavior, gonotrophic period, and vector survival. For instance, estimates of mosquito-bird contact rates and mosquito birth/mortality rates differ between the two model forms (see S17 and S19 Figs). These differences are consistent with previous studies, which have shown that warmer temperatures influence mosquito development rates, shorten the duration of the gonotrophic period and decrease the extrinsic incubation period of the virus, increasing disease transmission, but at the same time decrease vector survival [9–14]. Thus, the temperature-forced model appears to more accurately depict transmission dynamics and improves forecast accuracy.

Although the number of counties included in this study is not geographically exhaustive, the findings provide evidence that the methods presented can be flexibly applied to a diversity of regions to produce accurate forecasts. The 12 counties included here represent a diverse set of locations with differences in population density, ecosystems (e.g.. wet/dry, warm/cold), primary vectors, and mosquito monitoring practices. In spite of these differences the EAKF was still able optimize the forecast model for simulation and skillful forecast. We believe that the method is applicable to regions (e.g. the Northern Plains) not represented in this sample, provided appropriate mosquito monitoring and disease surveillance systems are in place. We also expect the temperature-forced model to function better than the baseline model given the improvements in forecast accuracy apparent across latitude and precipitation levels (see S1 Text and S7–S10 Figs).

The greater forecast accuracy for wet versus dry counties may be indicative of a need to represent hydrologic effects. Precipitation in dry counties may have a larger impact on vector abundance than in wet counties, where breeding habitats are more plentiful and thus not as dependent on rainfall [21] and irrigation practices [22]. Further, in drier counties, mosquito collections were more variable. Low mosquito trap numbers corrupt inference of the proportion of infected mosquitoes in a given week, and when collections fell below 300 in a week, these observations were discarded (see Methods and S1 Text). As a consequence, drier counties had more gaps in their mosquito infection data records. In the future, more robust collection data may improve forecast accuracy in drier regions.

Mosquito population dynamics vary with local conditions [20] and may play an important role determining WNV spillover risk. We tested incorporating vector abundance by using entomological risk rather than prevalence as the estimate of mosquito infection rate. This approach produced forecasts with similar, though somewhat lower accuracy for most outcomes (see S1 Text and S20 and S21 Figs). In the future, particularly should forecasts be generated at a smaller spatial scale, it will be important to reexamine inclusion of population dynamics in the model framework.

Among the different temperature-forcing approaches compared, we found evidence that observed seasonal temperature variability is important but inter annual differences are not. Both the climatological and daily-observed temperature-forced forecasts were more accurate than the baseline model for peak timing, peak magnitude and total human WNV cases during the majority of the outbreak season. In contrast, forcing with a seasonal average temperature or permuted weather performed worse than the baseline model. These findings indicate that a local seasonal temperature cycle improves forecast accuracy by giving the model additional biologically relevant structure.

It is interesting that forecasts with climatological temperature forcing were more accurate than those generated using observed temperature conditions. This finding indicates that short-term fluctuations of temperature due to synoptic variability may actually degrade forecast accuracy. Whether this effect is due to the transience of these signals, which corrupts filter optimization, or the simplicity of the model, which may not appropriately represent the effects of these fluctuations on transmission dynamics, is not clear.

Overall, inclusion of temperature forcing improves the forecast skill of our parsimonious WNV model and provides further evidence that temperature modulates rates of WNV transmission. Though these forecast models do not represent the full complexity of WNV transmission dynamics, including effects such as ongoing mosquito control efforts, within county spatial heterogeneity [28], bird migration [31], variable susceptibility among different hosts [36] and vectors [29], WNV strain variability [30], vertical transmission [29], and mosquito overwintering patterns [13], all of which can affect WNV transmission dynamics and spillover infection to humans, the system does generate accurate predictions of a number of outbreak metrics. As more data become available, inclusion of additional effects in the model structure, such as differences in risk based on population exposure probabilities, climatic zone and mosquito species, may be possible and may enable more accurate and nuanced forecast.

The present forecasts, if operationalized, could provide public health officials, mosquito control programs, and parks departments a quantified estimate of WNV spillover transmission risk. Such enumeration is important because decision makers often rely on a limited number of heuristic principles instead of assessing probabilities when making operational decisions. In general, these heuristic principals are useful, but sometimes lead to severe and systematic errors [37]. More quantitative decision tools, such as these forecasts, could thus provide a systematic platform for implementing response and control efforts.

As real-time forecast of WNV is operationalized, potential challenges will arise due to the need for robust timely data sets of both mosquito monitoring and human cases. In response to the emergence of WNV, an electronic surveillance system for arboviral disease, ArboNET, was developed by the Centers for Disease Control and Prevention (CDC) in 2000, and the CDC classified human cases of WNV as a nationally notifiable disease. ArboNET requires state and local health departments to report weekly human WNV case counts, along with infection of mosquitoes, birds and other animals, in order to monitor WNV activity across the country; however, this passive system has long lag times between when the data are generated and reported. In a retrospective study of Colorado hospital discharge data between 2003 and 2005 only 77% of hospital WNV cases were ever reported, and of those cases only 51% were reported within 7 days [38]. If real-time WNV forecast is to be operationalized, electronic monitoring systems, such as ArboNET, will need to shorten the delay between when data are collected and reported.

In addition to accelerated human case reporting, some areas of the US might benefit from more active mosquito monitoring. Mosquito monitoring practices vary around the country and are influenced by local socioeconomic factors, the tax base, and the public/political will to budget for mosquito surveillance [4]. Previous studies have shown that increasing mosquito trap density decreases measurement uncertainty and remains a cost effective way to monitor WNV activity [4]. If some mosquito abatement districts were to increase their monitoring budgets, additional mosquito traps early in the season might provide important data for improving forecast accuracy, and model studies could potentially provide information on how to design a monitoring program for optimal surveillance.

In addition to a more active monitoring program, operational forecasting would benefit from shorter lags between when mosquitoes are trapped and test results are received. The lag associated with tests results varies by abatement district. Large districts such as the city of Chicago or Maricopa County run in-house laboratories that provide same day or one-to-two day testing lags, whereas others ship samples to state laboratories, which subsidize testing, but have substantially longer lags: 7 to 10 days [39, 40]. For example, New York state lab fully subsides WNV testing for abatement districts [39] and the Louisiana state lab only charges $1.50 per WNV sample [40]. If St. Tammany Parish, LA, conducted in-house WNV sampling, each sample would cost approximately 10 times more than the state lab [40]. More work is needed to understand the costs associated with these lag times and the value of real-time data relative to the impact associated with each human cases of WNV, which is expensive and debilitating. For instance, the economic impact of an outbreak in Louisiana was significant with the median cost per human case of WNV around $11,000 (ranging from $830 -$218,600) [41]. In California the costs associated with neuroinvasive WNV and WNV fever are $75,600 and $1,430 [42], respectively (2016 US$). State subsidies might be better spent in providing counties with more resources for onsite testing that reduces the time between when mosquitoes are collected and a decision maker has quantified information pertaining to the outbreak.

The general under reporting of human WNV infections [38, 43, 44] is an observational bias, and we implicitly account for this bias in the forecasting model. That is, the parameter, *η*, which estimates mosquito-to-human transmission (see Methods), is necessarily only estimating recorded human cases. As long as roughly 4% of all human WNV infections are recorded each year [45]—and this rate appears relatively stable—the forecasts should perform reliably well. The human WNV case predictions are thus for the 1 in 25 cases that are clinically identified. However, the existence of a larger sub-clinical set of human WNV infections, whose numbers correlate with the clinically identified cases, must also be recognized.

Should resources be provided to generate timely data streams, real-time WNV forecasts could be operationalized. These real-time forecasts could be used as a decision support tool by public health officials, mosquito control programs, and parks departments to help target control of infectious mosquito populations, alert the public when WNV spillover transmission risk is elevated, and identify when to intensify blood donor screening.

## Methods

### Data

#### Mosquito data

In order to forecast WNV, abundant mosquito infection rate data are needed. We contacted 68 agencies that either collect or store mosquito-monitoring data. These agencies were contacted because historical WNV outbreaks had occurred in these regions, providing incentive for in-depth mosquito monitoring. Twenty-six agencies agreed to provide data, of which 12 different US counties (Fig 1) met the minimum requirements for forecasting: weekly mosquito monitoring with on average 15 mosquito pools sampled for a minimum of 10 weeks in a year [39, 40, 46–54]. In total, records for 110 WNV outbreaks were available for this study. See S1 Text for a more detailed description of the mosquito infection data, S22 Fig and S5 and S6 Tables.

#### Observed human cases

Weekly human cases of WNV were obtained from ArboNET, the national arboviral surveillance system and local county health departments [39, 40, 46–49, 55, 56]. Human cases of WNV were aggregated by week according to the date of illness onset (see S6 Table and S6 and S23 Fig). We assumed the error variance associated with each weekly observation, i.e. the observational error variance (OEV), was half the reported number of WNV cases for that week. If zero or one case was reported, the OEV was set to one (see S1 Text for more details on human cases of WNV).

#### Temperature data

Local temperature data for each of the 12 counties included in this study were assembled for 1981–2000 and each outbreak year. Temperature (T) data were compiled from the National Land Data Assimilation System (NLDAS) project-2 dataset at an hourly time step on a 0.125° regular grid from 1979 through the present [57]. Air temperatures at 2 meters above the surface are derived through spatial interpolation, temporal disaggregation and vertical adjustment from station measurements and National Center for Environmental Prediction North American Regional Reanalysis [58]. These hourly data were then averaged to daily resolution and a 20-year daily climatology was constructed for each county (see S24 Fig). Daily temperature climatology for a given county is simply the historical average of temperature conditions each day (Fig 2).

### Modeling approach

#### Compartmental model

Forecasts of WNV were generated using compartmental models that describe the transmission dynamics of WNV among mosquitoes and birds, as well as spillover transmission to humans. Two different compartmental models were tested: one with temperature forcing and the other without. Both models employed a standard susceptible-infected-recovered (SIR) epidemiological construct. The model with temperature forcing is represented by the following equations:

$$\frac{dS_{M}}{dt}\;=\;\mu_{M}N_{M}-\kappa (T)\beta (t)S_{M}\frac{I_{B}}{N_{B}}-\mu_{M}S_{M}-\alpha S_{M}$$ (1)

$$\frac{dI_{M}}{dt}\;=\;\kappa (T)\beta (t)S_{M}\frac{I_{B}}{N_{B}}-\mu_{M}I_{M}+\alpha S_{M}$$ (2)

$$\frac{dS_{B}}{dt}\;=\;-\kappa (T)\beta (t)I_{M}\frac{S_{B}}{N_{B}}$$ (3)

$$\frac{dI_{B}}{dt}\;=\;\kappa (T)\beta (t)I_{M}\frac{S_{B}}{N_{B}}-\frac{I_{B}}{\delta_{B}}$$ (4)

$$\frac{dI_{H}}{dt}\;=\;Poisson(\eta I_{M})$$ (5)

where *S*_*M*_ is the number of susceptible mosquitoes, *μ*_*M*_ is the mosquito birth and death rate (constant population), *N*_*M*_ is the mosquito population which is held constant, *t* is time in days, *β(t)* is the contact rate or probability of transmission between birds and mosquitoes at time *t*, κ(*T*) is the extrinsic incubation period for a given temperature *T*, α is the rate of WNV seeding into the local model domain for the first 50 days, *I*_*M*_ is the number of infected mosquitoes, *N*_*B*_ is the bird population which is held constant, *I*_*B*_ is the number of infected birds, *S*_*B*_ is the number of susceptible birds in the population, *δ*_*B*_ is the bird infection recovery rate, *I*_*H*_ is the number of infected humans, and *η* is a scalar that accounts for the contact rate and probability of transmission from mosquitoes to humans. The probability of WNV spilling over to humans is simulated using a Poisson random number generator, while the other equations are continuous and deterministic. The system of equations is solved using a 1-day time step with a Runge Kutta 4th order ODE solver.

The contact rate, *β(t)*, is designed to represent the switch in mosquito feeding preference from avian to mammalian hosts [33–35] and is modeled as a generalized logistic equation:

$$\beta (t)\;=\;A+\frac{K-A}{1+e^{\left(-r(t-t_{0})\right)}}$$ (6)

where *A* is the lower asymptote, *K* is the upper asymptote, *r* is the growth rate, and *t*_*0*_ is the inflection point.

Temperature may also influence transmission efficiency. Here we model the extrinsic incubation period as a function of temperature using published rates for *Cx*. *Tarsalis* [10], which are also similar for *Cx*. *pipiens* complex [59]:

κ(T)=-0.132+CT(t) (7)

where *T* is daily time varying temperature in degrees Celsius and C is a regression coefficient (here to be estimated using the EAKF). If κ(*T*) is below the thermal minimum, less than 0, then the function is set to 0. For the baseline forecast, κ(*T*) is set to 1. The structural difference between the temperature-forced and baseline contact rates, representing the probability of transmission between birds and mosquitoes, is seen in S25 Fig.

#### Retrospective forecast

For each annual outbreak, a 300-member ensemble of the compartmental model was initiated (see S1 Text for details on initial conditions) and run until the point of observation. Each week, available observations of human WNV cases and mosquito infection rates were assimilated into the model using the EAKF (see S1 Text for details on the model EAKF system). Through this assimilation process, the model state space and parameters are iteratively updated to better represent current local outbreak dynamics. From week 20 until the end of the calendar year, starting with the first observation of infectious mosquitoes, forecasts were generated following the most recent update of the model state variables and parameters. That is, the forecasts were generated by integrating the latest posterior estimate for the WNV compartmental model (Eqs 1–7) through time until the end of the outbreak season. This process was repeated weekly, with each successive forecast having one additional week of observational data assimilated. Each 300-member ensemble forecast was repeated 10 times with different randomly selected initial conditions and evaluated for accuracy according to prescribed forecast metrics (see Results and S1 Text for more details).

#### Effects of temperature variability

We also explored how knowledge of observed temperature, in theory, would improve our ability to forecast. The use of observed temperature is not realistic for real-time forecast, as future temperature conditions are not known; however, as applied here, it can be used to forecast WNV outbreaks retrospectively and determine if such information would, in theory, improve forecast accuracy.

We compared 6 different scenarios in which temperature was applied differently into the model. The 6 scenarios are: 1) the baseline model without temperature forcing, 2) our principal temperature forcing: daily climatology temperature forcing, 3) observed daily temperature forcing (i.e., real weather, which is not feasible for use in real time forecasting as such data would not be available), 4) observed seasonal average temperature forcing, 5) permuted observed temperature values for a given year and location; and 6) permuted temperature values from the historical 1981–2000 record. S13 Fig shows examples of these different temperature forcing time series.

In addition to evaluating forecast ensemble mean trajectory accuracy for the 6 different scenarios in which temperature forcing was applied differently, we also assessed whether forecast error differed significantly for climatology temperature forcing versus observed daily temperature forcing and observed daily temperature forcing versus the baseline model using a Wilcoxon signed-rank test.

#### Code availability

The code that support the findings of this study are available from the corresponding author upon request.

## Supporting information

S1 Text (DOCX)

S1 Fig The baseline ensemble forecast. The blue dotted lines are the ensemble mean forecasts from the baseline model, the grey area is the spread of the ensemble forecast (light grey represents area between the 10th and 90th percentile and the darker grey area represents the spread between the 25th and 75th percentile), blue lines are the ensemble mean posterior distribution, orange *x*’s are data points assimilated into the model, orange * are future observations, and the green lines represent the target area of an accurate forecast. A forecast was deemed accurate if: 1) peak timing was within ±1 week of the observed peak of infectious mosquitoes; 2) peak infection rate was within ±25% of the observed peak infection rate; and 3) human WNV cases were within ±25% of the total number of reported cases.
(TIFF)

S2 Fig The temperature-forced ensemble forecast. The red dotted lines are the ensemble mean forecasts from the temperature forced model, the grey area is the spread of the ensemble forecast (light grey represents area between the 10th and 90th percentile and the darker grey area represents the spread between the 25th and 75th percentile), red lines are the ensemble mean posterior distribution, orange *x*’s are data points assimilated into the model and orange * are future observations, and the green lines represent the target area of an accurate forecast. A forecast was deemed accurate if: 1) peak timing was within ±1 week of the observed peak of infectious mosquitoes; 2) peak infection rate was within ±25% of the observed peak infection rate; and 3) human WNV cases were within ±25% of the total number of reported cases.
(TIFF)

S3 Fig The fraction of forecasts accurate for both the temperature-forced model (red) and baseline model (blue) as a function of calendar week for the number of human WNV cases reported 1, 2, 3 and 4 weeks in the future. A forecast was deemed accurate if the forecast number of human WNV cases were within ±25% or ±1 case of the number of reported cases from point of forecast to the number of weeks in the future, whichever was larger.
(TIFF)

S4 Fig The fraction of forecasts accurate for both the temperature-forced model (red) and baseline model (blue) as a function of predicted lead for the number of human WNV cases reported 1, 2, 3 and 4 weeks in the future. A forecast was deemed accurate if the forecast number of human WNV cases were within ±25% or ±1 case of the number of reported cases from point of forecast to the number of weeks in the future, whichever was larger. Note that lead week is shown with respect to the week of peak mosquito infection. The size of the dot at each lead week represents the number of forecast generated for that lead week; larger dots indicate more forecasts were generated with a particular predicted lead.
(TIFF)

S5 Fig The fraction of human cases reported for the 110 outbreaks relative to the week of peak infectious mosquito rates (top) and calendar week (bottom). (JPG)

S6 Fig The fraction of forecasts accurate for both the temperature-forced model (red) and baseline model (blue) as a function of lead week for the metrics human WNV cases, peak timing (week of peak mosquito infection rates), peak infection rate, and total infectious mosquitoes. A forecast was deemed accurate if: 1) peak timing was earlier or later than average; 2) peak infection rate was higher or lower than average; 3) total infectious mosquitoes were higher or lower than average; and 4) human WNV cases were higher or lower than average. Note that for all metrics lead week is shown with respect to the week of peak mosquito infection. The size of the dot at each lead week represents the number of forecast generated for that lead week; larger dots indicate more forecasts were generated with a particular predicted lead.
(TIFF)

S7 Fig Southern counties: The fraction of forecasts accurate for both the temperature-forced model (red) and baseline model (blue) as a function of lead week for the metrics human WNV cases, peak timing (week of peak mosquito infection rates), peak infection rate, and total infectious mosquitoes. A forecast was deemed accurate if: 1) peak timing was within ±1 week of the observed peak of infectious mosquitoes; 2) peak infection rate was within ±25% of the observed peak infection rate; 3) total infectious mosquitoes were within ±25% of the observed; and 4) human WNV cases were within ±25% or ±1 case of the total number of reported cases, whichever was larger. Note that for all metrics lead week is shown with respect to the week of peak mosquito infection. The size of the dot at each lead week represents the number of forecast generated for that lead week; larger dots indicate more forecasts were generated with a particular predicted lead. Counties south of 40° N were used in this analysis (Maricopa County AZ, Orange County CA, Sacramento County CA, Yolo County CA, Iberia Parish LA, St Tammany Parish LA, and Clark County NV).
(TIFF)

S8 Fig Northern counties: The fraction of forecasts accurate for both the temperature-forced model (red) and baseline model (blue) as a function of lead week for the metrics human WNV cases, peak timing (week of peak mosquito infection rates), peak infection rate, and total infectious mosquitoes. A forecast was deemed accurate if: 1) peak timing was within ±1 week of the observed peak of infectious mosquitoes; 2) peak infection rate was within ±25% of the observed peak infection rate; 3) total infectious mosquitoes were within ±25% of the observed; and 4) human WNV cases were within ±25% or ±1 case of the total number of reported cases, whichever was larger. Note that for all metrics lead week is shown with respect to the week of peak mosquito infection. The size of the dot at each lead week represents the number of forecast generated for that lead week; larger dots indicate more forecasts were generated with a particular predicted lead. Counties north of 40° N were used in this analysis (Boulder County CO, Weld County CO, Cook County IL, and Allen County IN, and Suffolk County NY).
(TIFF)

S9 Fig Dry counties: The fraction of forecasts accurate for both the temperature-forced model (red) and baseline model (blue) as a function of lead week for the metrics human WNV cases, peak timing (week of peak mosquito infection rates), peak infection rate, and total infectious mosquitoes. A forecast was deemed accurate if: 1) peak timing was within ±1 week of the observed peak of infectious mosquitoes; 2) peak infection rate was within ±25% of the observed peak infection rate; 3) total infectious mosquitoes were within ±25% of the observed; and 4) human WNV cases were within ±25% or ±1 case of the total number of reported cases, whichever was larger. Note that for all metrics lead week is shown with respect to the week of peak mosquito infection. The size of the dot at each lead week represents the number of forecast generated for that lead week; larger dots indicate more forecasts were generated with a particular predicted lead. Counties that receive less than 10 mm/day of precipitation annually were used in this analysis (Maricopa County AZ, Orange County CA, Boulder County CO, Weld County CO, and Clark County NV).
(TIFF)

S10 Fig Wet counties: The fraction of forecasts accurate for both the temperature-forced model (red) and baseline model (blue) as a function of lead week for the metrics human WNV cases, peak timing (week of peak mosquito infection rates), peak infection rate, and total infectious mosquitoes. A forecast was deemed accurate if: 1) peak timing was within ±1 week of the observed peak of infectious mosquitoes; 2) peak infection rate was within ±25% of the observed peak infection rate; 3) total infectious mosquitoes were within ±25% of the observed; and 4) human WNV cases were within ±25% or ±1 case of the total number of reported cases, whichever was larger. Note that for all metrics lead week is shown with respect to the week of peak mosquito infection. The size of the dot at each lead week represents the number of forecast generated for that lead week; larger dots indicate more forecasts were generated with a particular predicted lead. Counties that receive more than 10 mm/day of precipitation annually were used in this analysis (Sacramento County CA, Yolo County CA, Cook County IL, Allen County IN, Iberia Parish LA, St Tammany Parish LA, and Suffolk County NY).
(TIFF)

S11 Fig Wet versus dry counties: The fraction of forecasts accurate, using the temperature-forced model as a function of lead week for the metrics human WNV cases, peak timing (week of peak mosquito infection rates), peak infection rate, and total infectious mosquitoes. A forecast was deemed accurate if: 1) peak timing was within ±1 week of the observed peak of infectious mosquitoes; 2) peak infection rate was within ±25% of the observed peak infection rate; 3) total infectious mosquitoes were within ±25% of the observed; and 4) human WNV cases were within ±25% or ±1 case of the total number of reported cases, whichever was larger. Note that for all metrics lead week is shown with respect to the week of peak mosquito infection. The size of the dot at each lead week represents the number of forecast generated for that lead week; larger dots indicate more forecasts were generated with a particular predicted lead. Counties that receive more than 10 mm/day of precipitation annually were considered wet (green), Sacramento County CA, Yolo County CA, Cook County IL, Allen County IN, Iberia Parish LA, St Tammany Parish LA, and Suffolk County NY and dry counties received less than 10 mm/day of precipitation annually (yellow), Maricopa County AZ, Orange County CA, Boulder County CO, Weld County CO, and Clark County NV.
(TIFF)

S12 Fig Northern versus southern counties: The fraction of forecasts accurate, using the temperature-forced model as a function of lead week for the metrics human WNV cases, peak timing (week of peak mosquito infection rates), peak infection rate, and total infectious mosquitoes. A forecast was deemed accurate if: 1) peak timing was within ±1 week of the observed peak of infectious mosquitoes; 2) peak infection rate was within ±25% of the observed peak infection rate; 3) total infectious mosquitoes were within ±25% of the observed; and 4) human WNV cases were within ±25% or ±1 case of the total number of reported cases, whichever was larger. Note that for all metrics lead week is shown with respect to the week of peak mosquito infection. The size of the dot at each lead week represents the number of forecast generated for that lead week; larger dots indicate more forecasts were generated with a particular predicted lead. Northern counties are north of 40° N latitude (Boulder County CO, Weld County CO, Cook County IL, and Allen County IN, and Suffolk County NY) and southern county are south of 40° N (Maricopa County AZ, Orange County CA, Sacramento County CA, Yolo County CA, Iberia Parish LA, St Tammany Parish LA, and Clark County NV).
(TIFF)

S13 Fig Temperature forcing time series for Sacramento County during 2009 to 2011. 5 different temperature data streams were used in the model: 1) daily climatology from 1981 to 2000 as described in the main manuscript (red line); 2) observed local daily temperature (yellow line); 3) observed seasonal average temperature, calculated for each outbreak year and location as the mean daily temperature for all weeks the forecast was generated (one temperature value is used for the whole season and fluctuates from season to season, yellow dashed line); 4) permuted observed temperature values: randomly select temperature values from all days in a season (yellow o); and 5) permuted temperature values from the climatology: randomly select temperature values from the day of year which were forecasted in that season (red o).
(TIFF)

S14 Fig Comparison of forecast accuracy as a function of lead week using different temperature forcings for the metrics human WNV cases, peak timing (week of peak mosquito infection rates), peak infection rate, and total infectious mosquitoes. Red represents climatology and yellow represents observed local temperature—daily climatology (red line), observed local daily temperature (yellow line), observed seasonal average temperature (yellow dashed), permuted observed local daily temperature (yellow dot) and permuted climatology (red dot). A forecast was deemed accurate if: 1) peak timing was within ±1 week of the observed peak of infectious mosquitoes; 2) peak infection rate was within ±25% of the observed peak infection rate; 3) total infectious mosquitoes were within ±25% of the observed; and 4) human WNV cases were within ±25% or ±1 case of the total number of reported cases, whichever was larger. Note that for all metrics lead week is shown with respect to the week of peak mosquito infection. The size of the dot at each lead week represents the number of forecast generated for that lead week; larger dots indicate more forecasts were generated with a particular predicted lead.
(TIFF)

S15 Fig Sensitivity of findings to accuracy definition: The fraction of forecasts accurate for both the temperature-forced model (red) and baseline model (blue) as a function of lead week for the metrics human WNV cases, peak timing (week of peak mosquito infection rates), peak infection rate, and total infectious mosquitoes. A forecast was deemed accurate if: 1) peak timing was within ±2, ±3, or ±4 weeks of the observed peak of infectious mosquitoes; 2) peak infection rate was within ±20%, ±30% or ±50% of the observed peak infection rate; 3) total infectious mosquitoes were within ±20%, ±30% or ±50% of the observed; and 4) human WNV cases were within ±20%, ±30% or ±50% or ±1 case of the total number of reported cases, whichever was larger. Note that for all metrics lead week is shown with respect to the week of peak mosquito infection.
(TIFF)

S16 Fig The fraction of forecasts accurate for both the temperature-forced model (red) and baseline model (blue) as a function of week of the year for the metric total weekly infectious mosquitoes over a season. A forecast was deemed accurate if total weekly infectious mosquitoes over a season were within ±25% of the observed.
(TIFF)

S17 Fig The average posterior contact rate, or probability of transmission between birds and mosquitoes, as a function of time for the temperature-forced model (red) and baseline, non-temperature forced model, (blue). (TIFF)

S18 Fig The average posterior spillover rate, or probability of transmission between mosquitoes and humans, as a function of time for the temperature-forced model (red) and baseline, non-temperature forced, model, (blue). (TIFF)

S19 Fig The average posterior birth death rate of mosquitoes as a function of time for the temperature-forced model (red) and baseline, non-temperature forced model, (blue). (TIFF)

S20 Fig The fraction of forecasts accurate for both the temperature-forced model (red) and baseline model (blue) using both prevalence (solid line) and entomological risk (dashed line) to represent infectious mosquitoes, shown as a function of week of the year for the metrics human WNV cases, peak timing (week of peak mosquito infection rates), peak infection rate, and total infectious mosquitoes. A forecast was deemed accurate if: 1) peak timing was within ±1 week of the observed peak of infectious mosquitoes; 2) peak infection rate was within ±25% of the observed peak infection rate; 3) total infectious mosquitoes were within ±25% of the observed; and 4) human WNV cases were within ±25% or ±1 case of the total number of reported cases, whichever was larger.
(TIFF)

S21 Fig The fraction of forecasts accurate for both the temperature-forced model (red) and baseline model (blue) using both prevalence (solid line) and entomological risk (dashed line) to represent infectious mosquitoes, shown as a function of lead week for the metrics human WNV cases, peak timing (week of peak mosquito infection rates), peak infection rate, and total infectious mosquitoes. A forecast was deemed accurate if: 1) peak timing was within ±1 week of the observed peak of infectious mosquitoes; 2) peak infection rate was within ±25% of the observed peak infection rate; 3) total infectious mosquitoes were within ±25% of the observed; and 4) human WNV cases were within ±25% or ±1 case of the total number of reported cases, whichever was larger. Note that for all metrics lead week is shown with respect to the week of peak mosquito infection. The size of the dot at each lead week represents the number of forecast generated for that lead week; larger dots indicate more forecasts were generated with a particular predicted lead.
(TIFF)

S22 Fig Box and whisker plots of the number of infected mosquitoes per 1,000 tested for each outbreak evaluated. The boxes and whiskers show the median (red horizontal line), 25th and 75th percentiles (box boundaries), the whiskers mark the highest and lowest values within a multiple of 1.5 of the interquartile range of the box boundaries, and outliers are shown as red (+).
(TIFF)

S23 Fig Weekly average human cases of WNV, dated by the week of illness onset, for years forecast by county. (TIFF)

S24 Fig 20-year daily climatology of 2m above-ground temperature (blue line) for each county. These data are the daily input for the temperature-forced model. The red dotted line is 14.3°C, which represents the minimum threshold for virus development [10, 59].
(JPG)

S25 Fig Structural differences in how the temperature-forced model (red) and baseline, non-temperature forced model, (blue) define the contact rate, or probability of transmission between birds and mosquitoes, as a function of time. (TIFF)

S26 Fig The natural log of the weekly number of mosquitoes sampled compared to the width of the 95% confidence interval of estimated proportion of infectious mosquitoes. The uncertainty associated with the proportion of mosquitoes infected becomes sensible when 300 or more mosquitoes are sampled.
(TIFF)

S1 Table Wilcoxon signed-rank test comparing predicted error between the two modeling approaches. Absolute error was calculated and compared for each prediction of observed peak of infectious mosquitoes, maximum mosquito infection rate, and the total number of human cases over the entire season, whereas root mean squared error (RMSE) was used to calculate future forecasts of the total number of mosquitoes observed over the season. 1 indicates the temperature-forced model forecasts had statistically significantly less error than the baseline model and -1 indicates the baseline model forecasts had statistically significant less error.
(DOCX)

S2 Table Wilcoxon signed-rank test comparing predicted error between the two modeling approaches. Absolute error was calculated and compared for each prediction of total number of human cases over the next week, 2 weeks, 3 weeks and 4 weeks. 1 indicates the temperature-forced model forecasts had statistically significantly less error than the baseline model and -1 indicates the baseline model forecasts had statistically significant less error.
(DOCX)

S3 Table Wilcoxon signed-rank test comparing predicted error between the two modeling approaches. Absolute error was calculated and compared for each prediction of observed peak of infectious mosquitoes, maximum mosquito infection rate, and the total number of human cases over the entire season, whereas root mean squared error (RMSE) was used to calculate future forecasts of the total number of mosquitoes observed over the season. 1 indicates the reported daily observed temperature-forced model forecasts had statistically significantly less error than the baseline model and -1 indicates the baseline model forecasts had statistically significant less error.
(DOCX)

S4 Table Wilcoxon signed-rank test comparing predicted error between the using weather and climatology in the temperature-forced model. Absolute error was calculated and compared for each prediction of observed peak of infectious mosquitoes, maximum mosquito infection rate, and the total number of human cases over the entire season, whereas root mean squared error (RMSE) was used to calculate future forecasts of the total number of mosquitoes observed over the season. 1 indicates the daily observed temperature-forced model forecasts had statistically significantly less error than the daily climatology temperature-forced forecasts and -1 indicates the daily climatology temperature-forced forecasts had statistically significant less error.
(DOCX)

S5 Table Overview of the dominant WNV vector in each county along with the trap type used to monitor these mosquitoes. (DOCX)

S6 Table Overview of human WNV cases, mosquito infection rates, and mosquito data. (DOCX)

S7 Table Annual average precipitation, climatology and latitude for each county evaluated. (DOCX)

S8 Table Correlation between the seasonal sum of weekly observed infected mosquito (prevalence) and the total number of human WNV cases over the season or seasonal sum of weekly observed infected mosquito (Entomological risk, prevalence times weekly average number of mosquitoes per trap night) and the total number of human WNV cases over the season. (DOCX)
