# Supplementary material for: Use of temperature to improve West Nile virus forecasts
Source: PLoS Comput Biol. 2018 Mar 9;14(3):e1006047. doi: 10.1371/journal.pcbi.1006047 (PMC5862506; doi:10.1371/journal.pcbi.1006047)
Supplement: S1 Text — (DOCX) [file pcbi.1006047.s001.docx]

**Supplementary Information Appendix**

**Title: Use of Temperature to Improve West Nile Virus Forecasts**

**Authors:** Nicholas B. DeFelice, Zachary D. Schneider, Eliza Little, Christopher Barker, Kevin A. Caillouet, Scott R. Campbell, Dan Damian, Patrick Irwin, Herff M.P. Jones, John Townsend, & Jeffrey Shaman

**Data.**

*Mosquito Data*

Mosquito surveillance data were obtained from 12 different US counties (Figure 1) [1-11]. These counties conducted weekly mosquito surveillance subject to budgetary constraints, the severity of WNV, mosquito nuisance problems and weather. In total, records for 110 county-seasons were available for this study. Mosquito surveillance programs in counties with a more clearly defined mosquito season trapped from the beginning of June until the end of October, while counties in warmer regions monitored for disease year round. Weekly mosquito surveillance was conducted using gravid, CO_2_-baited, light, and sentinel traps, depending on the county and mosquito species (see table S5). The total number of traps set each week within a season and county varied.

This study focuses on WNV-assayed pools of *Culex* spp. mosquitoes as only a few *Culex* mosquito species drive enzootic transmission [12]. In the Northeast and Midwest we focus on *Cx. pipiens* and *Cx. restuans* [13, 14], in the Southeast U.S. *Cx. quinquefasciatus,* Southwest *Cx. tarsalis* and *Cx. quinquefasciatus,* and West *Cx. tarsalis* and *Cx. pipiens*, which have been shown to be the main drivers of the vector-avian host enzootic WNV transmission cycle, as well as the vector(s) responsible for human spillover [12]. Table S5 lists the primary WNV vector by county.

Collected mosquitoes were identified and counted, and a portion of mosquitoes were pooled and assayed for the presence of WNV. These assays were performed using either real-time reverse transcription-polymerase chain reaction (PCR) or a rapid analyte measurement platform (RAMP). The number and size of the pools tested for WNV varied each week.

We combined all pools of mosquitoes sampled in a week and used a maximum likelihood approach to estimate the total weekly proportion of positive mosquitoes [15]. If less than 300 mosquitoes per week were sampled, the observation was excluded and not assimilated, due to the high uncertainty of the maximum likelihood estimate (Fig. S26, see below for description of maximum likelihood estimation). Fig. S22 shows the weekly variability of the prevalence of infected mosquitoes. Table S6 presents statistics on mosquito collection by county and season, including the number of weeks sampled, the estimated peak mosquito infection rate, the week of peak infection rates (peak timing), the number of *Culex* pools assayed for WNV, and the number of WNV positive *Culex* pools.

*Mosquito infection rates*

It is assumed that if a pool tests positive at least one mosquito is WNV positive whereas a negative result indicates all mosquitoes are WNV negative. The maximum likelihood estimation (MLE) is considered the most appropriate estimate of infection rate when either pool size varies or infection levels are high [16]. County average mosquito infection rates were estimated using MLE and a binomial distribution, and all pooled samples for the county over a given week. Specifically, the log-likelihood equation for data ***x*=**(*x_1_, x_2_, … x_M_*) is:

$\boldsymbol{l}\left( \boldsymbol{p;x} \right)\boldsymbol{=l}\left( \boldsymbol{p} \right)\boldsymbol{=}\sum_{\boldsymbol{i=0}}^{\boldsymbol{M}} \boldsymbol{x}_{\boldsymbol{i}}\boldsymbol{log[1-}\left( \boldsymbol{1-p}\boldsymbol{)}^{\boldsymbol{m}_{\boldsymbol{i}}} \right]\boldsymbol{+}\boldsymbol{log(1-p)}\sum_{\boldsymbol{i=0}}^{\boldsymbol{M}} {\boldsymbol{m}_{\boldsymbol{i}}\boldsymbol{(}\boldsymbol{n}_{\boldsymbol{i}}\boldsymbol{-x}}_{\boldsymbol{i}}\boldsymbol{)}$ **(S1)**

where *x_i_* is the number of positive samples for a given pool size, *m_i_* is the distinct number of mosquitoes sampled in a pool, *n_i_* is the number of times the distinct pool size was sampled, and M is the number of distinct pool sizes. The solution to *p,* the maximum likelihood estimate of equation S6, was obtained using the Newton-Raphson method. The ninety-five percent confidence intervals were also numerically computed around each point estimate using the following equation:

$\frac{\sum_{\boldsymbol{i=0}}^{\boldsymbol{M}} \frac{\boldsymbol{m}_{\boldsymbol{i}}^{\boldsymbol{2}}\boldsymbol{n}_{\boldsymbol{i}}\boldsymbol{(1-p}\boldsymbol{)}^{\boldsymbol{m}_{\boldsymbol{i}}\boldsymbol{-2}}}{\boldsymbol{1-(1-p}\boldsymbol{)}^{\boldsymbol{m}_{\boldsymbol{i}}}}}{\left[ \frac{\boldsymbol{1}}{\boldsymbol{1-p}}\sum_{\boldsymbol{i=1}}^{\boldsymbol{M}} \left[ \frac{\boldsymbol{m}_{\boldsymbol{i}}\boldsymbol{x}_{\boldsymbol{i}}}{\boldsymbol{1-(1-p}\boldsymbol{)}^{\boldsymbol{m}_{\boldsymbol{i}}}}\boldsymbol{-}\boldsymbol{m}_{\boldsymbol{i}}\boldsymbol{n}_{\boldsymbol{i}} \right] \right]^{\frac{\boldsymbol{1}}{\boldsymbol{2}}}}\boldsymbol{=\pm1.96}$. **(S2)**

Variance was derived from these confidence intervals and used as the observational error variance (OEV) for a given week. If the OEV was less than 15, we set the OEV to 15. For more details on the MLE method, see Biggerstaff [15].

*Observed Human Cases*

WNV is a nationally notifiable disease. State and local health departments report the weekly number of human WNV cases to the Centers for Disease Control and Prevention through the ArboNET surveillance system [17]. Weekly human cases of WNV were obtained from ArboNET, the national arboviral surveillance system for 2001 to 2014 [18]. Due to changes in ArboNET’s data release polices, data for 2015 and 2016 were obtained from county health departments [1, 3-5, 8, 10, 19].

Weekly reported human cases of WNV, both neuroinvasive and non-neuroinvasive, were used in this study. Human cases of WNV were aggregated by week according to the date of illness onset with each week defined as Sunday to Saturday (Table S6 and Fig. S23).

*Initial Model Conditions*

A 300-member ensemble of compartmental model simulations was run [20] for each outbreak season (weeks 21 to 52). Each ensemble member was initialized with the following states: *S_m_*_(0)_ =4,000, *I_m_*_(0)_=*0* *S_B_*_(0)_=500, *I_B_*_(0)_=*0* and *I_H_*_(0)_=0. Model parameters were then randomly selected from the following initial prior uniform distributions: μ=U(0.05,0.08) [21, 22], δ= U(3.8,6.0) [23] *r*= U(-0.2, -0.05), and for the baseline model κ(*T*) =1, *A*= U(0.01,0.03), *K*= U(0.06,0.12) whereas for the temperature model *C*=(0.0078, 0.0105) [24], *A*= U(0.3,0.5), *K*= U(0.7,1.2). Differences in *A* and *K* among the two models are implemented for scaling. In addition, *t_0_* was initialized once the first infectious pool of mosquitoes was observed and set to U(5 weeks, 10 weeks), and *η*= U(0,0.005) for counties with a population less than 2 million people and *η*= U(0,0.015) for counties with 2 million or more people. Initial priors for mosquito expected lifespan [21, 22], bird duration of infection [23] and extrinsic incubation period [24] were selected from the literature whereas initial priors for *β(t)* and *η* were determined during synthetic testing of the model-EAKF system [25]. All simulations were seeded with infected mosquitoes, *α*, during the first 50 days of the integration period, at a rate of 1 infected mosquito per 250,000 susceptible mosquitoes.

*Model-EAKF system*

The EAKF data assimilation method has previously been used in conjunction with compartmental epidemiological models and infectious disease data to simulate diseases, including influenza, dengue and Ebola [26-30], as well as WNV [25]. This data assimilation technique uses Bayes’ rule to provide an updated posterior estimate of the system state at a given point in time, using current observations and the model prior estimate. In the presence of observations with prescribed observational error variance (OEV), the EAKF adjusts the ensemble of model-simulated state variables toward the target true state. Unobserved state variables and parameters are then adjusted as well using cross ensemble co-variability. When observational error is not correlated, the filter can be applied to multiple, simultaneously observed variables sequentially, which is done here for observations of infectious mosquitoes and human cases. For further details on the EAKF algorithm see Anderson [31]; for further information on its application to infectious disease modeling and forecasting see Shaman and Karspeck [26].

In this study, a 300-member ensemble simulation of the WNV compartmental model (Equations 1-7) was run in conjunction with the EAKF and observations of mosquito WNV infection rates and human WNV cases. The model-EAKF system contained the modeled state space composed of the five disease state variables and seven or eight parameters depending on model form, i.e. ***z_t_***=(*S_M_, I_M_, S_B_, I_B_, I_H_, μ, A, K, r, t_0_, δ_B_, η* and *C*). Whenever an observation of human WNV cases and mosquito WNV infection rates, ***y_t_*=(***I_M_* and *I_H_***),** was available—in this study human WNV observations were reported weekly and mosquito observations occurred when more than 300 mosquitoes were sampled in a given week—the EAKF algorithm was used to assimilate those new observations and update the model observed state variables and unobserved state variables and parameters. The model was then integrated forward to the next observation, using the updated (posterior) model state variables and parameters, and the data assimilation updating process was repeated. Through this iterative optimization process, the ensemble of model simulations was aligned to better represent current local outbreak dynamics.

*Forecast Metrics*

The accuracy of the forecasts was assessed through comparison of the forecast ensemble mean trajectory and observed outcomes. A short-term forecasts was deemed accurate if the mean trajectory of human cases over the next 1, 2, 3, or 4 weeks was within ±25% or ±1 case of the total number cases, whichever was greater, during that time period. A seasonal forecast was deemed accurate if: 1) it peaked within ±1 week of the observed peak of infectious mosquitoes; 2) the maximum mosquito infection rate was within ±25% of the observed peak infection rate; 3) the total number of infectious mosquitoes over the entire season was within ±25% of the observed; and 4) the total number of human cases over the entire season was within ±25% or ±1 case of the total number of reported cases, whichever was larger. Additionally, forecasts were examined across all counties and years. All forecasts with the same lead were grouped or week of year, and the fraction of accurate forecasts was quantified.

Seasonal forecasts accuracy was also compared to historical average outbreaks to determine if the system could simply forecast accurately whether an outbreak was earlier or later than average or larger or smaller than average. The average outbreak for each county was defined as the mean value for the 4 metrics (total human WNV cases, total infectious mosquitoes, peak infectious mosquitoes and peak timing) for all years excluding the forecast year. A seasonal forecast was deemed accurate if the forecast coincided with the outbreak being either earlier or later than average or higher or lower than average.

The performances of the temperature-forced and baseline models were compared using a Wilcoxon signed-rank test to assess whether forecast error of the two modeling approaches is statistically different. Absolute error was calculated and compared for predictions of observed peak of infectious mosquitoes, maximum mosquito infection rate, and the total number of human cases over the entire season, while root mean squared error (RMSE) was used to calculate the total weekly forecast error associated with the number of infectious mosquito observations over the season.

*Ecological Differences*

We further evaluated forecast accuracy as a function of geographic location and precipitation levels. For geographic location, we grouped the forecasts into northern or southern counties. Specifically, counties north of 40°N were classified as northern counties and possess roughly a maximum of 21 weeks with temperatures above 14.3°C, the theoretical threshold for viral development (11) and the remaining counties below 40°N were classified as southern counties and have longer WNV seasons. For the second grouping we classified counties as wet if receiving greater than 10 mm/day of annual precipitation, and dry if 10mm/day or less [32] (see Table S7).

Evaluating the differences among these groupings we saw that counties experiencing more precipitation were more accurately forecast than dry counties (SI Fig. S10); however, for the northern versus southern counties the results were mixed (SI Fig. S11). Specifically, northern counties were more accurate forecasting the total number of human cases and the total number of infectious mosquitoes, whereas the southern counties were more accurate forecasting the peak timing of infectious mosquitoes and peak magnitude of infectious mosquitoes.

The difference in forecast accuracy between the wet and dry counties may be embedded in the assumption that the mosquito population is constant and the difference in the mosquito population dynamics [33]. We observed that when less than 300 mosquitoes per week were sampled, which often occurred early in the season, observations were highly variable and less reliable (see SI Fig. S23). Thus, we excluded such observations in order to avoid substantial over- or under- estimation of the proportion of mosquitoes infected. However, weeks with less than 300 mosquitoes are disproportionately found within dry counties. Indeed, for dry counties 20% of the time we had to exclude infected mosquito observations due to undersampling, whereas in wet counties 95% of the time we had ample mosquito data.

## Entomological risk

Entomological risk and prevalence of infected mosquitoes can both be used as indicators of WNV activity and both have been associated with the number of WNV disease cases [34, 35]. Either indicator can be used to help infer the risk of human spillover and both were considered for use as the observation of mosquito infection and assimilation into the model.

For the 9 locations where we had numbers of mosquitoes per trap night, we examined the correlation between the seasonal sum of weekly observed infected mosquitoes and the total number of human WNV cases over the season (Table S8). The seasonal sum of weekly observed infected mosquito was calculated in two ways: 1) prevalence derived through maximum likelihood estimation from mosquito pool data; and 2) entomological risk, prevalence times the weekly average number of mosquitoes per trap night. Six counties had statically significant correlations for prevalence while 5 did for entomological risk.

Seasonal forecasts using entomological risk for the observed infectious mosquitoes showed similar results to the prevalence model when comparing the baseline model to the temperature forced model. The baseline model generated more accurate forecasts as a function of calendar week for total number of human cases, peak timing, peak magnitude, and infectious mosquitoes over the season early in the forecast but by the middle of the season (the end of July), weeks 32, 32, 33,and 35, for these 4 metrics, respectively, the temperature-forced model forecasts were more accurate (Fig. S17).

Comparing the entomological risk observations using forecast lead week, we see that the temperature-forced model is more accurate at predicting human cases, peak infection rates and seasonal infection rates beginning two weeks prior to the peak. In addition it was more accurate forecasting peak timing most weeks beginning 5 weeks prior to the peak (Fig. S18).

For both the baseline model and the temperature-forced model, entomological risk can be used to forecast outbreaks of WNV; however, for the 9 counties evaluated, on average prevalence produced more accurate forecasts. Indeed, prevalence was more accurate for all 4 indicators when temperature forcing was applied; however, the baseline model had more mixed results.

1. Barker C. California Vectorborne Disease Surveillance System. California Vectorborne Disease Surveillance System2016.

2. Raman V, Bramley CT. Mosquito Pooled WNV Test Record 2008-2016. Southern Nevada Health District and Clark County Department of Public Works2016.

3. Caillouet KA. Mosquito Pooled WNV Test Record 2006-2014. St. Tammany Parish Mosquito Abatement District2016.

4. Campbell SR. Mosquito Pooled WNV Test Record 2001-2015. Suffolk County Department of Health Services Arthropod-Borne Disease Laboratory2016.

5. Fiess D, Blauvelt J. Mosquito Pooled WNV Test Record 2007-2016. Fort Wayne-Allen County Department of Health2016.

6. Geery P. Mosquito Pooled WNV Test Record 2007-2014. Des Plaines Valley Mosquito Abatement District2016.

7. Irwin P. Mosquito Pooled WNV Test Record 2007-2014. Northwest Mosquito Abatement District2016.

8. Jones HMP. Mosquito Pooled WNV Test Record 2006-2016. Iberia Parish Mosquito Abatement District2016.

9. Smith S. West Nile Virus (WNV) Mosquito Test Results. Chicago Department of Public Health 2016.

10. Townsend J, Damian D. Mosquito Pooled WNV Test Record 2006-2016. Maricopa County Environmental Services Department2016.

11. Weissmann M. Mosquito Pooled WNV Test Record 2007-2016. Colorado Mosquito Control2016.

12. Petersen LR, Brault AC, Nasci RS. West Nile virus: review of the literature. Jama. 2013;310(3):308-15.

13. Andreadis TG, Anderson JF, Vossbrinck CR, Main AJ. Epidemiology of West Nile virus in Connecticut: a five-year analysis of mosquito data 1999-2003. Vector-Borne & Zoonotic Diseases. 2004;4(4):360-78.

14. Kulasekera VL, Kramer L, Nasci RS, Mostashari F, Cherry B, Trock SC, et al. West Nile virus infection in mosquitoes, birds, horses, and humans, Staten Island, New York, 2000. Emerging Infectious Diseases. 2001;7(4):722.

15. Biggerstaff BJ. Confidence intervals for the difference of two proportions estimated from pooled samples. Journal of agricultural, biological, and environmental statistics. 2008;13(4):478-96.

16. Gu W, Lampman R, Novak R. Assessment of arbovirus vector infection rates using variable size pooling. Medical and Veterinary Entomology. 2004;18(2):200-4.

17. Centers for Disease Control and Prevention. West Nile Virus in the United States: Guidelines for surveillance, prevention, and control. Fort Collins, Colorado: Centers for Disease Control and Prevention. Retrieved from <http://www.cdc.gov/westnile/resources/pdfs/wnvGuidelines.pdf>; 2013.

18. Centers for Disease Control and Prevention. ArboNETData- base West Nile virus disease and other arboviral diseases - United States. West Nile virus disease cases and deaths reported to CDC by year and clinical presentation,1999‐2014; 2015.

19. Colton L. Colorado human WNV cases 2015-2016. Communicable Disease Branch Disease Control and Environmental Epidemiology Division Colorado Department of Public Health and Environment2016.

20. Yang W, Karspeck A, Shaman J. Comparison of filtering methods for the modeling and retrospective forecasting of influenza epidemics. PLoS Comput Biol. 2014;10(4):e1003583.

21. Wonham MJ, de-Camino-Beck T, Lewis MA. An epidemiological model for West Nile virus: invasion analysis and control applications. Proceedings of the Royal Society of London B: Biological Sciences. 2004;271(1538):501-7.

22. Hartley DM, Barker CM, Le Menach A, Niu T, Gaff HD, Reisen WK. Effects of temperature on emergence and seasonality of West Nile virus in California. The American journal of tropical medicine and hygiene. 2012;86(5):884-94.

23. Komar N, Langevin S, Hinten S, Nemeth N, Edwards E, Hettler D, et al. Experimental infection of North American birds with the New York 1999 strain of West Nile virus. Emerging infectious diseases. 2003;9(3):311.

24. Reisen WK, Fang Y, Martinez VM. Effects of temperature on the transmission of West Nile virus by Culex tarsalis (Diptera: Culicidae). Journal of medical entomology. 2006;43(2):309-17.

25. DeFelice NB, Little E, Campbell SR, Shaman J. Ensemble forecast of human West Nile virus cases and mosquito infection rates. Nature Communications. 2017;8:14592.

26. Shaman J, Karspeck A. Forecasting seasonal outbreaks of influenza. Proceedings of the National Academy of Sciences. 2012;109(50):20425-30.

27. Shaman J, Karspeck A, Yang W, Tamerius J, Lipsitch M. Real-time influenza forecasts during the 2012–2013 season. Nature communications. 2013;4.

28. Shaman J, Yang W, Kandula S. Inference and forecast of the current West African Ebola outbreak in Guinea, Sierra Leone and Liberia. PLoS currents. 2014;6.

29. Yang W, Cowling BJ, Lau EH, Shaman J. Forecasting Influenza Epidemics in Hong Kong. PLoS computational biology. 2015;11(7).

30. Yamana TK, Kandula S, Shaman J. Superensemble forecasts of dengue outbreaks. Journal of The Royal Society Interface. 2016;13(123):20160410.

31. Anderson JL. An ensemble adjustment Kalman filter for data assimilation. Monthly weather review. 2001;129(12):2884-903.

32. Xie P, Arkin PA. Analyses of global monthly precipitation using gauge observations, satellite estimates, and numerical model predictions. Journal of climate. 1996;9(4):840-58.

33. Morin CW, Comrie AC. Regional and seasonal response of a West Nile virus vector to climate change. Proceedings of the National Academy of Sciences. 2013;110(39):15620-5.

34. Bolling BG, Barker CM, Moore CG, Pape WJ, Eisen L. Seasonal patterns for entomological measures of risk for exposure to Culex vectors and West Nile virus in relation to human disease cases in northeastern Colorado. Journal of medical entomology. 2009;46(6):1519-31.

35. Little E, Campbell SR, Shaman J. Development and validation of a climate-based ensemble prediction model for West Nile Virus infection rates in Culex mosquitoes, Suffolk County, New York. Parasites & Vectors. 2016;9(1):443.
