# Supplementary figures and images for: Use of temperature to improve West Nile virus forecasts
Source: PLoS Comput Biol. 2018 Mar 9;14(3):e1006047. doi: 10.1371/journal.pcbi.1006047 (PMC5862506; doi:10.1371/journal.pcbi.1006047)

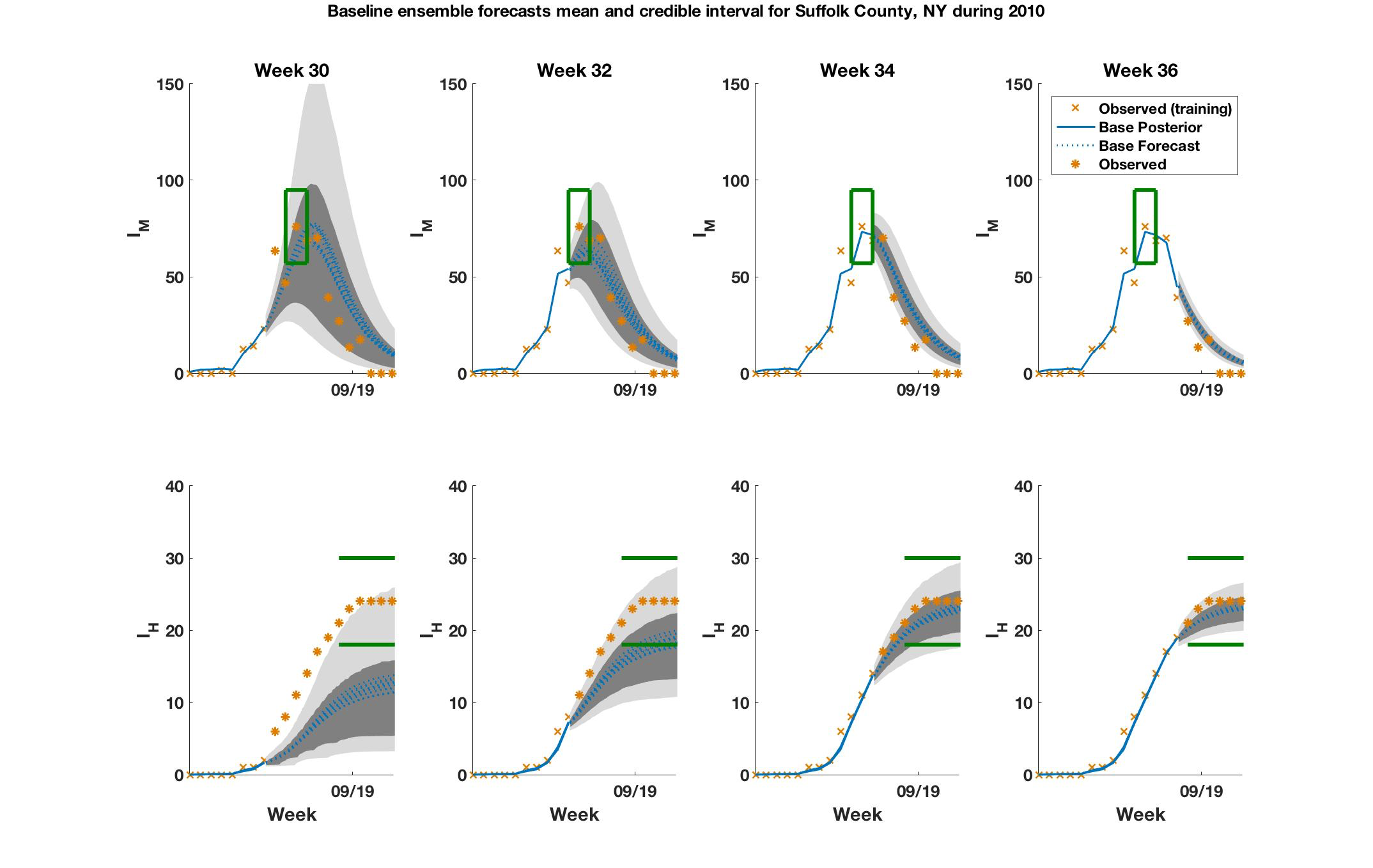

Supplement: S1 Fig — The blue dotted lines are the ensemble mean forecasts from the baseline model, the grey area is the spread of the ensemble forecast (light grey represents area between the 10th and 90th percentile and the darker grey area represents the spread between the 25th and 75th percentile), blue lines are the ensemble mean posterior distribution, orange x’s are data points assimilated into the model, orange * are future observations, and the green lines represent the target area of an accurate forecast. A forecast was deemed accurate if: 1) peak timing was within ±1 week of the observed peak of infectious mosquitoes; 2) peak infection rate was within ±25% of the observed peak infection rate; and 3) human WNV cases were within ±25% of the total number of reported cases. (TIFF) [file pcbi.1006047.s002.tiff]

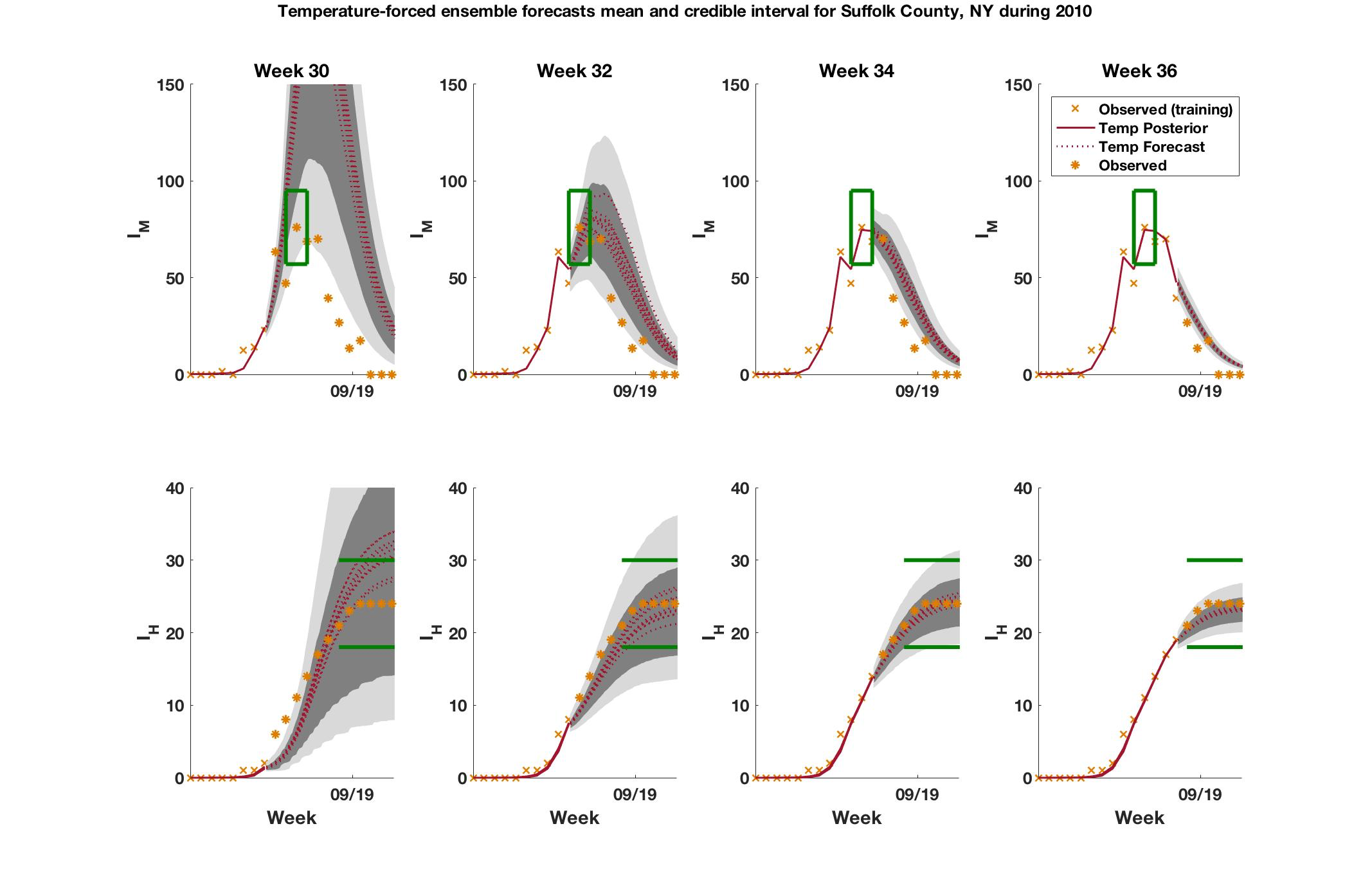

Supplement: S2 Fig — The red dotted lines are the ensemble mean forecasts from the temperature forced model, the grey area is the spread of the ensemble forecast (light grey represents area between the 10th and 90th percentile and the darker grey area represents the spread between the 25th and 75th percentile), red lines are the ensemble mean posterior distribution, orange x’s are data points assimilated into the model and orange * are future observations, and the green lines represent the target area of an accurate forecast. A forecast was deemed accurate if: 1) peak timing was within ±1 week of the observed peak of infectious mosquitoes; 2) peak infection rate was within ±25% of the observed peak infection rate; and 3) human WNV cases were within ±25% of the total number of reported cases. (TIFF) [file pcbi.1006047.s003.tiff]

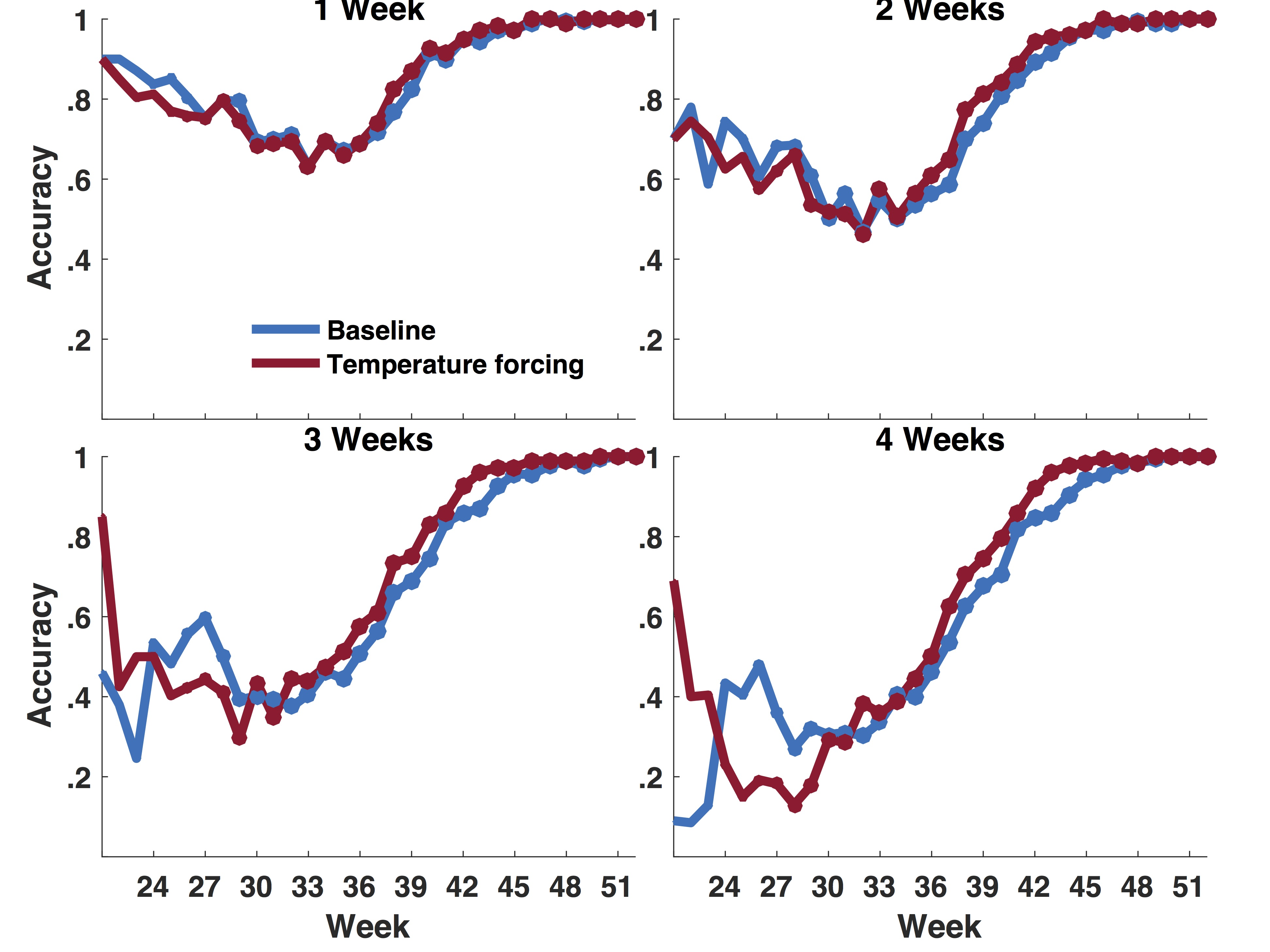

Supplement: S3 Fig — A forecast was deemed accurate if the forecast number of human WNV cases were within ±25% or ±1 case of the number of reported cases from point of forecast to the number of weeks in the future, whichever was larger. (TIFF) [file pcbi.1006047.s004.tiff]

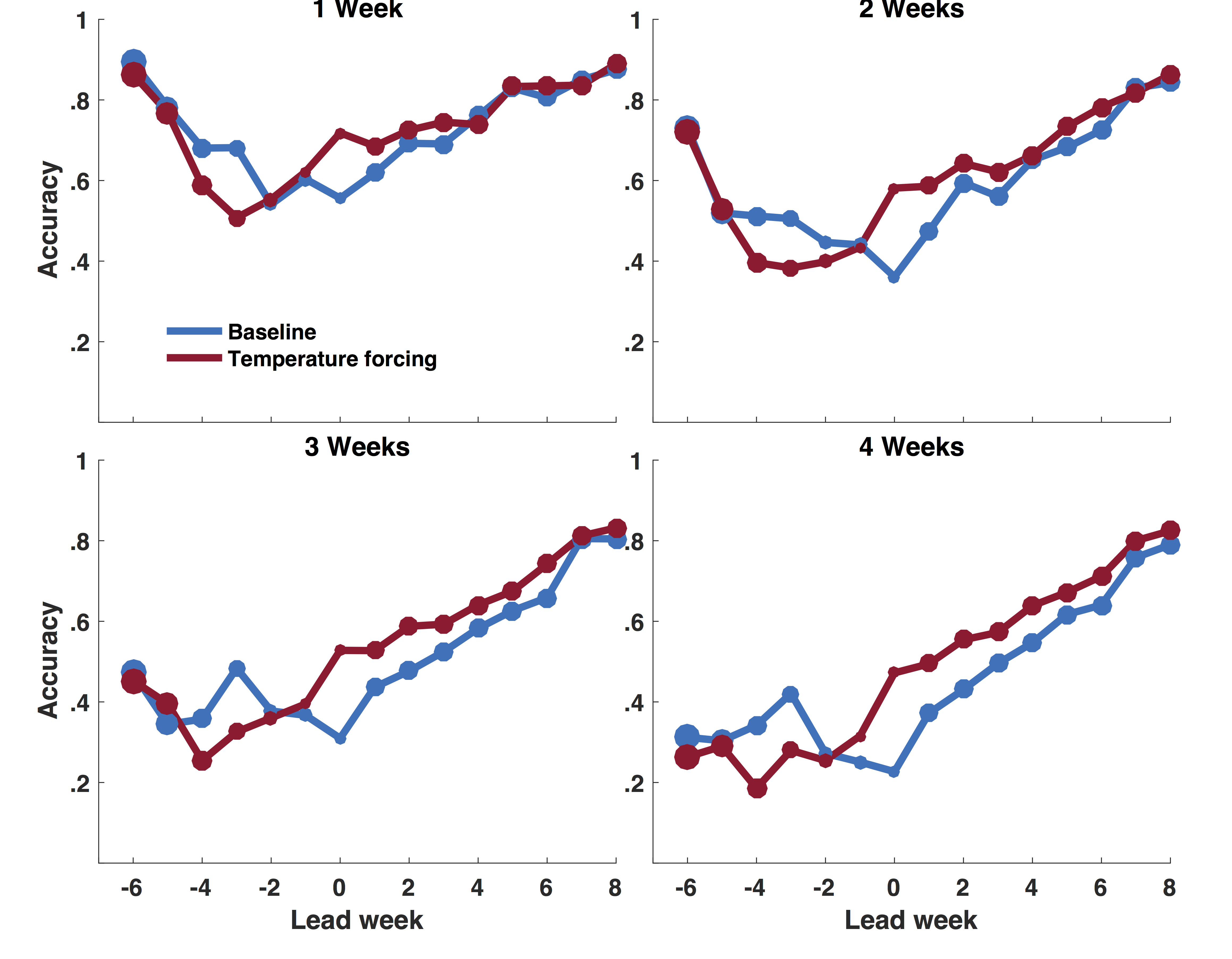

Supplement: S4 Fig — A forecast was deemed accurate if the forecast number of human WNV cases were within ±25% or ±1 case of the number of reported cases from point of forecast to the number of weeks in the future, whichever was larger. Note that lead week is shown with respect to the week of peak mosquito infection. The size of the dot at each lead week represents the number of forecast generated for that lead week; larger dots indicate more forecasts were generated with a particular predicted lead. (TIFF) [file pcbi.1006047.s005.tiff]

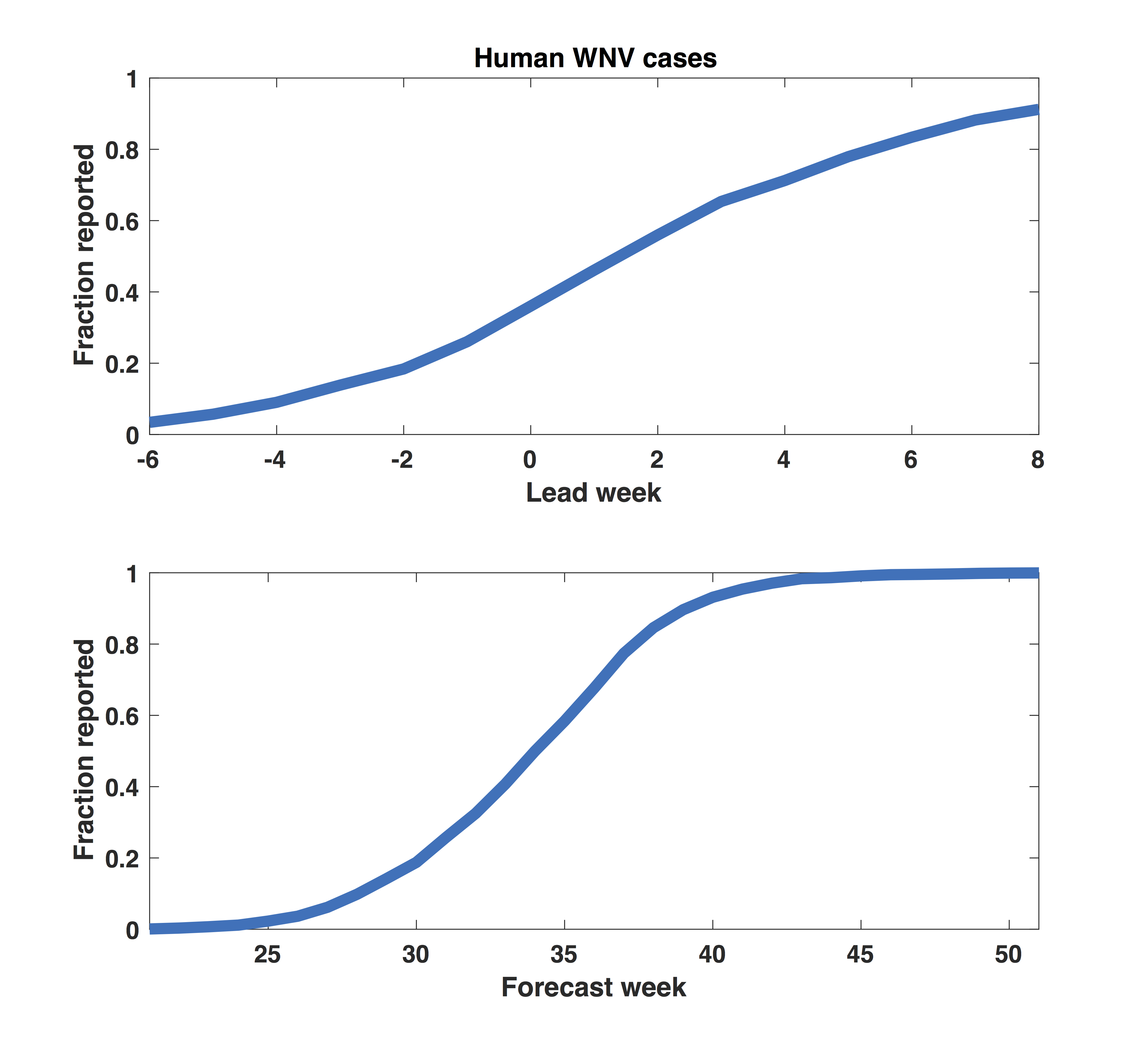

Supplement: S5 Fig — (JPG) [file pcbi.1006047.s006.jpg]

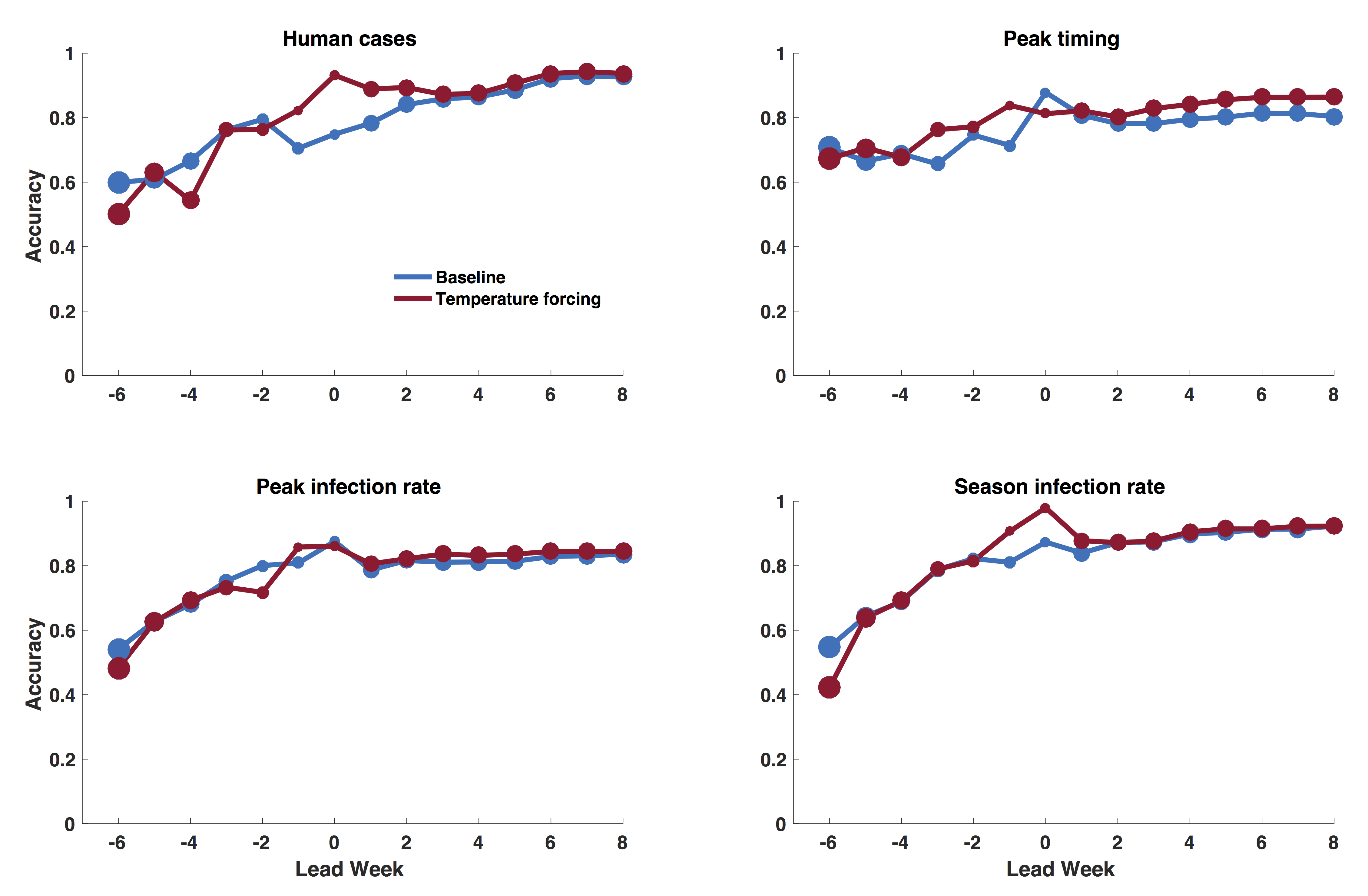

Supplement: S6 Fig — A forecast was deemed accurate if: 1) peak timing was earlier or later than average; 2) peak infection rate was higher or lower than average; 3) total infectious mosquitoes were higher or lower than average; and 4) human WNV cases were higher or lower than average. Note that for all metrics lead week is shown with respect to the week of peak mosquito infection. The size of the dot at each lead week represents the number of forecast generated for that lead week; larger dots indicate more forecasts were generated with a particular predicted lead. (TIFF) [file pcbi.1006047.s007.tiff]

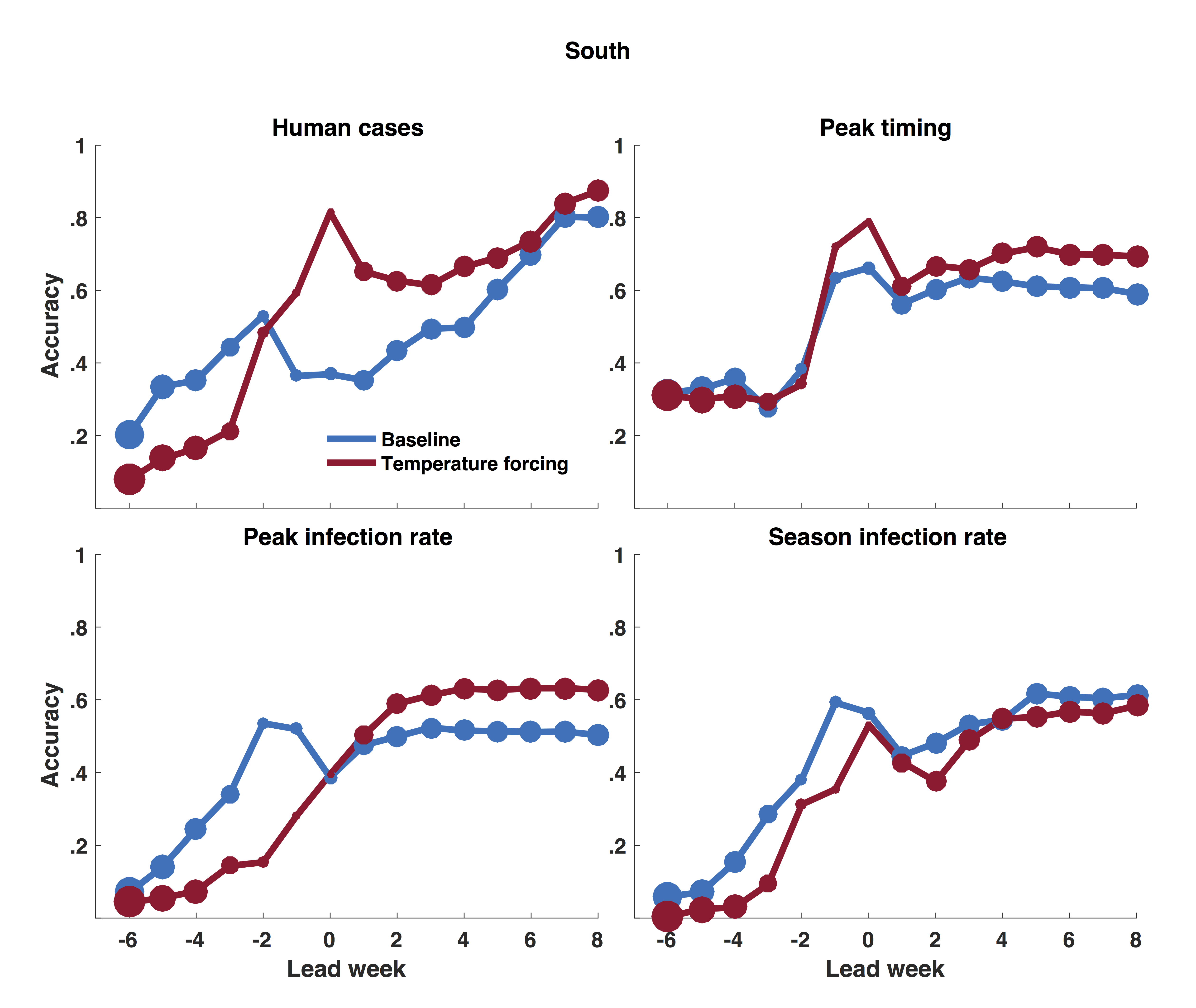

Supplement: S7 Fig — A forecast was deemed accurate if: 1) peak timing was within ±1 week of the observed peak of infectious mosquitoes; 2) peak infection rate was within ±25% of the observed peak infection rate; 3) total infectious mosquitoes were within ±25% of the observed; and 4) human WNV cases were within ±25% or ±1 case of the total number of reported cases, whichever was larger. Note that for all metrics lead week is shown with respect to the week of peak mosquito infection. The size of the dot at each lead week represents the number of forecast generated for that lead week; larger dots indicate more forecasts were generated with a particular predicted lead. Counties south of 40° N were used in this analysis (Maricopa County AZ, Orange County CA, Sacramento County CA, Yolo County CA, Iberia Parish LA, St Tammany Parish LA, and Clark County NV). (TIFF) [file pcbi.1006047.s008.tiff]

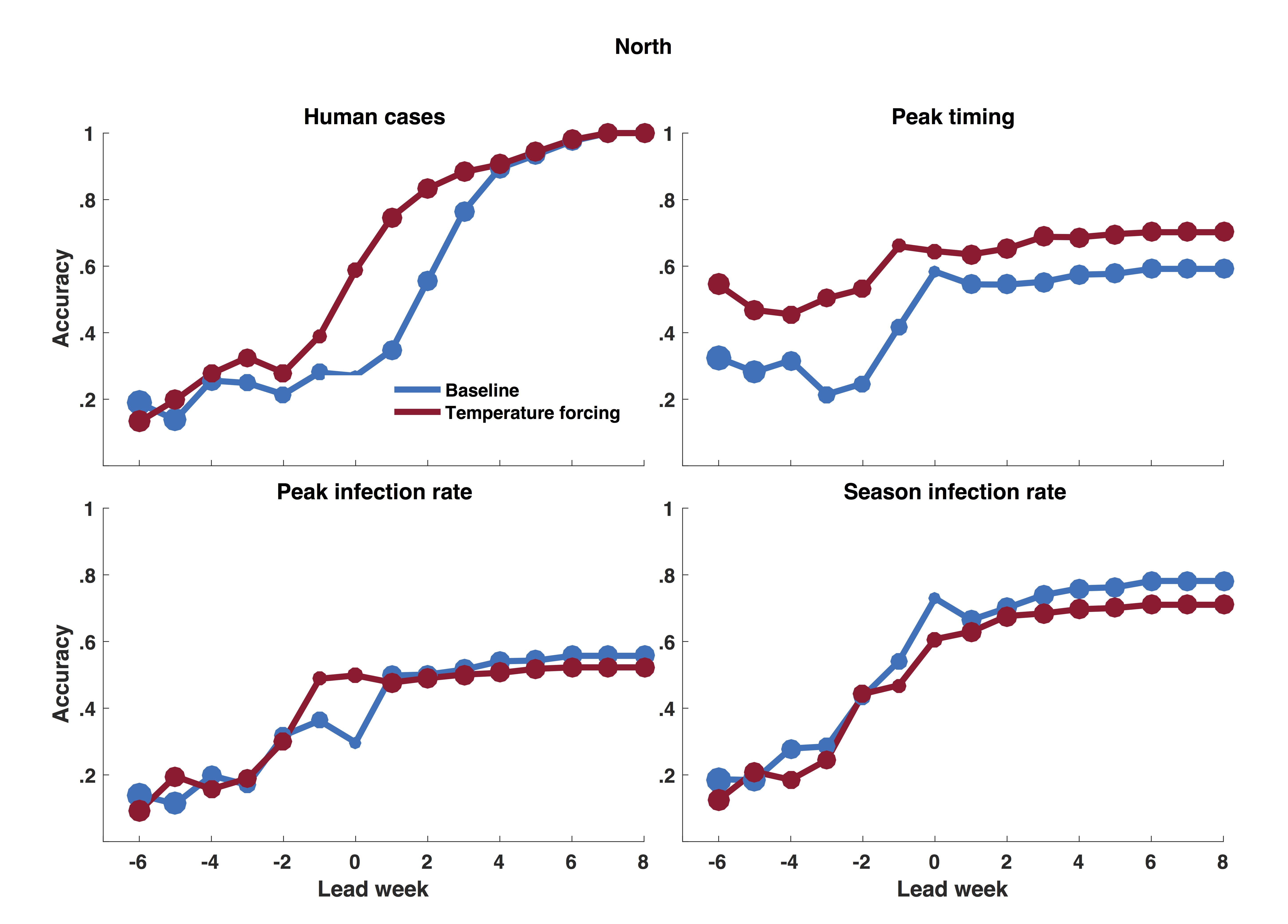

Supplement: S8 Fig — A forecast was deemed accurate if: 1) peak timing was within ±1 week of the observed peak of infectious mosquitoes; 2) peak infection rate was within ±25% of the observed peak infection rate; 3) total infectious mosquitoes were within ±25% of the observed; and 4) human WNV cases were within ±25% or ±1 case of the total number of reported cases, whichever was larger. Note that for all metrics lead week is shown with respect to the week of peak mosquito infection. The size of the dot at each lead week represents the number of forecast generated for that lead week; larger dots indicate more forecasts were generated with a particular predicted lead. Counties north of 40° N were used in this analysis (Boulder County CO, Weld County CO, Cook County IL, and Allen County IN, and Suffolk County NY). (TIFF) [file pcbi.1006047.s009.tiff]

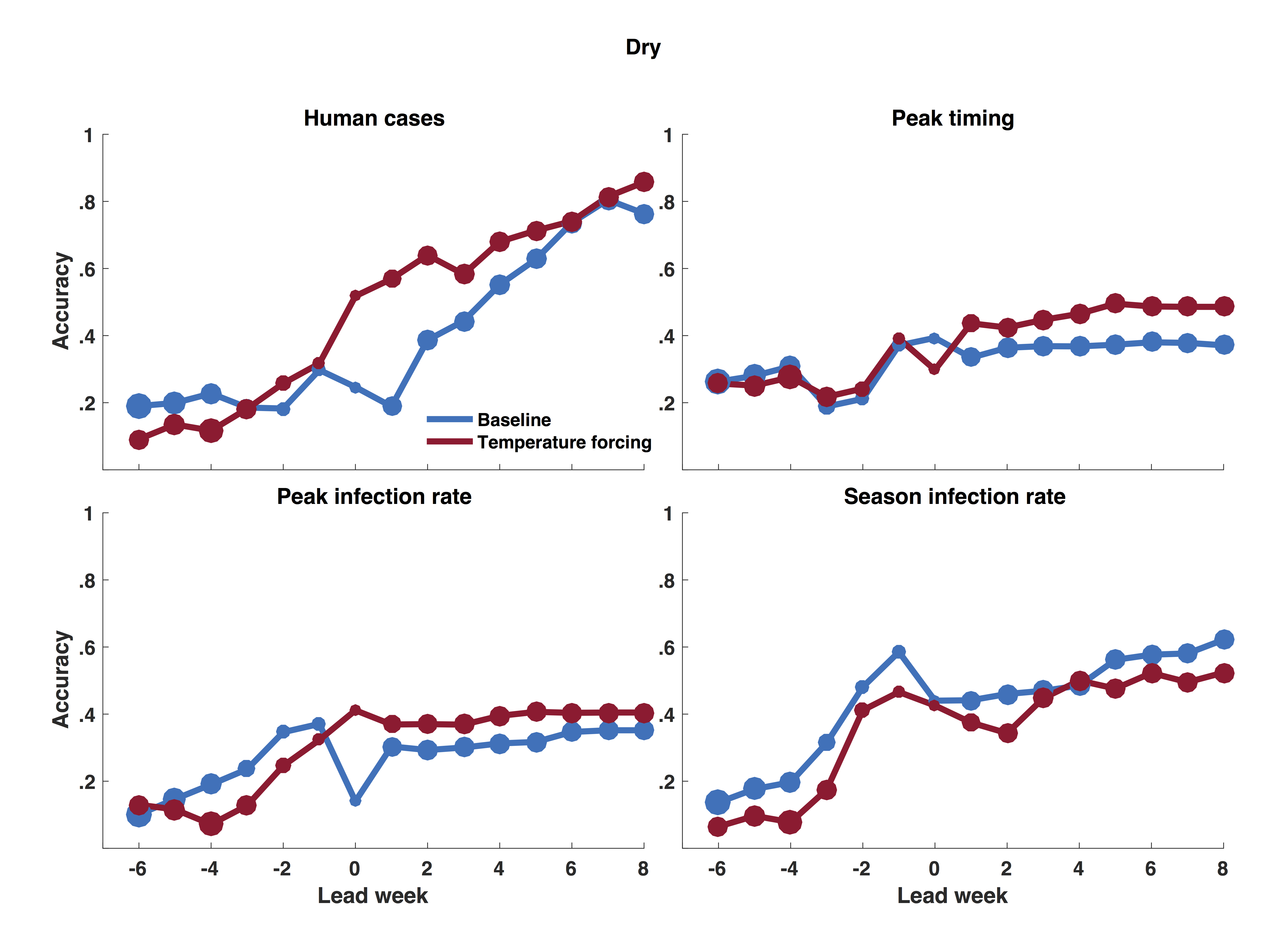

Supplement: S9 Fig — A forecast was deemed accurate if: 1) peak timing was within ±1 week of the observed peak of infectious mosquitoes; 2) peak infection rate was within ±25% of the observed peak infection rate; 3) total infectious mosquitoes were within ±25% of the observed; and 4) human WNV cases were within ±25% or ±1 case of the total number of reported cases, whichever was larger. Note that for all metrics lead week is shown with respect to the week of peak mosquito infection. The size of the dot at each lead week represents the number of forecast generated for that lead week; larger dots indicate more forecasts were generated with a particular predicted lead. Counties that receive less than 10 mm/day of precipitation annually were used in this analysis (Maricopa County AZ, Orange County CA, Boulder County CO, Weld County CO, and Clark County NV). (TIFF) [file pcbi.1006047.s010.tiff]

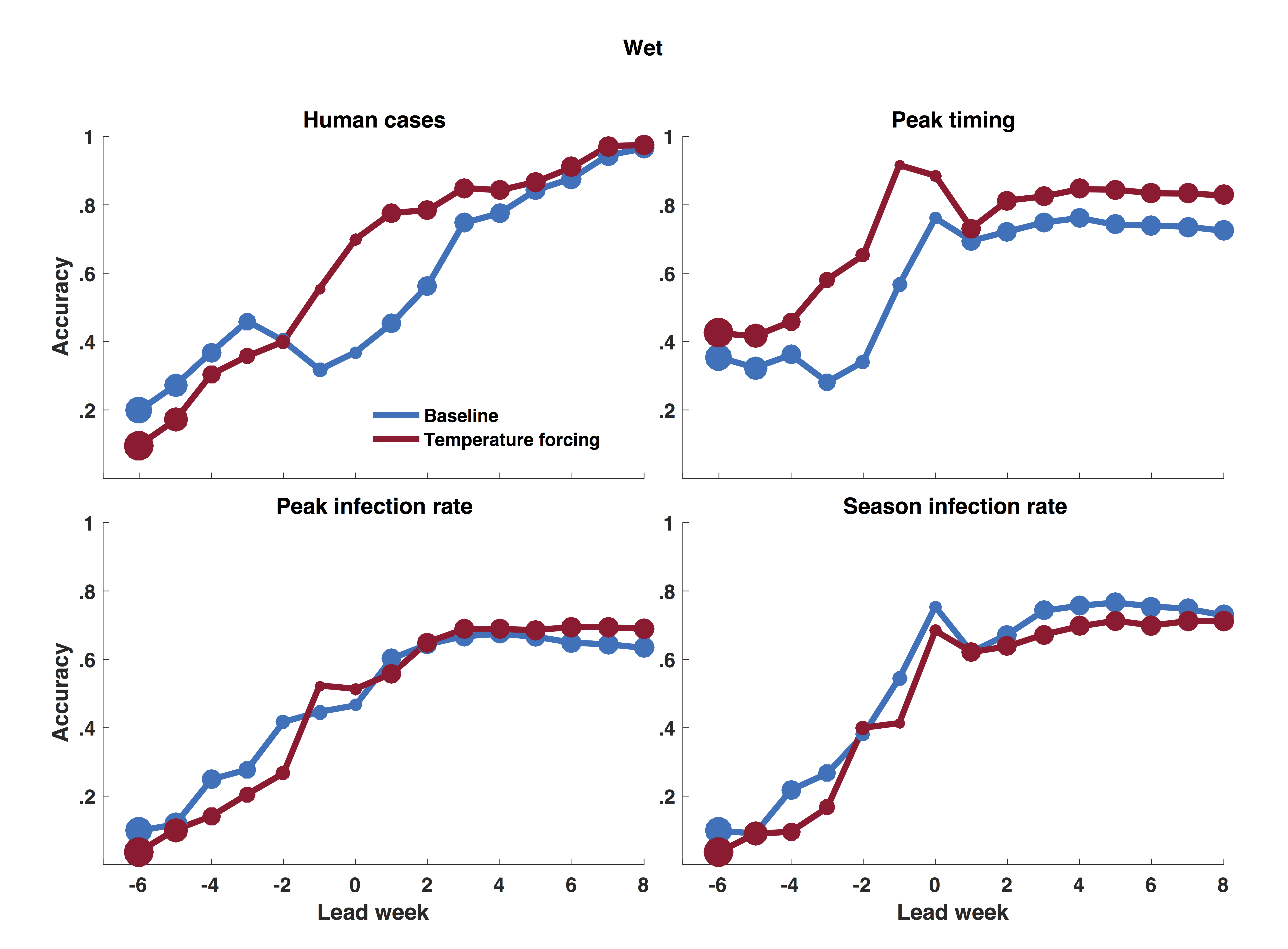

Supplement: S10 Fig — A forecast was deemed accurate if: 1) peak timing was within ±1 week of the observed peak of infectious mosquitoes; 2) peak infection rate was within ±25% of the observed peak infection rate; 3) total infectious mosquitoes were within ±25% of the observed; and 4) human WNV cases were within ±25% or ±1 case of the total number of reported cases, whichever was larger. Note that for all metrics lead week is shown with respect to the week of peak mosquito infection. The size of the dot at each lead week represents the number of forecast generated for that lead week; larger dots indicate more forecasts were generated with a particular predicted lead. Counties that receive more than 10 mm/day of precipitation annually were used in this analysis (Sacramento County CA, Yolo County CA, Cook County IL, Allen County IN, Iberia Parish LA, St Tammany Parish LA, and Suffolk County NY). (TIFF) [file pcbi.1006047.s011.tiff]

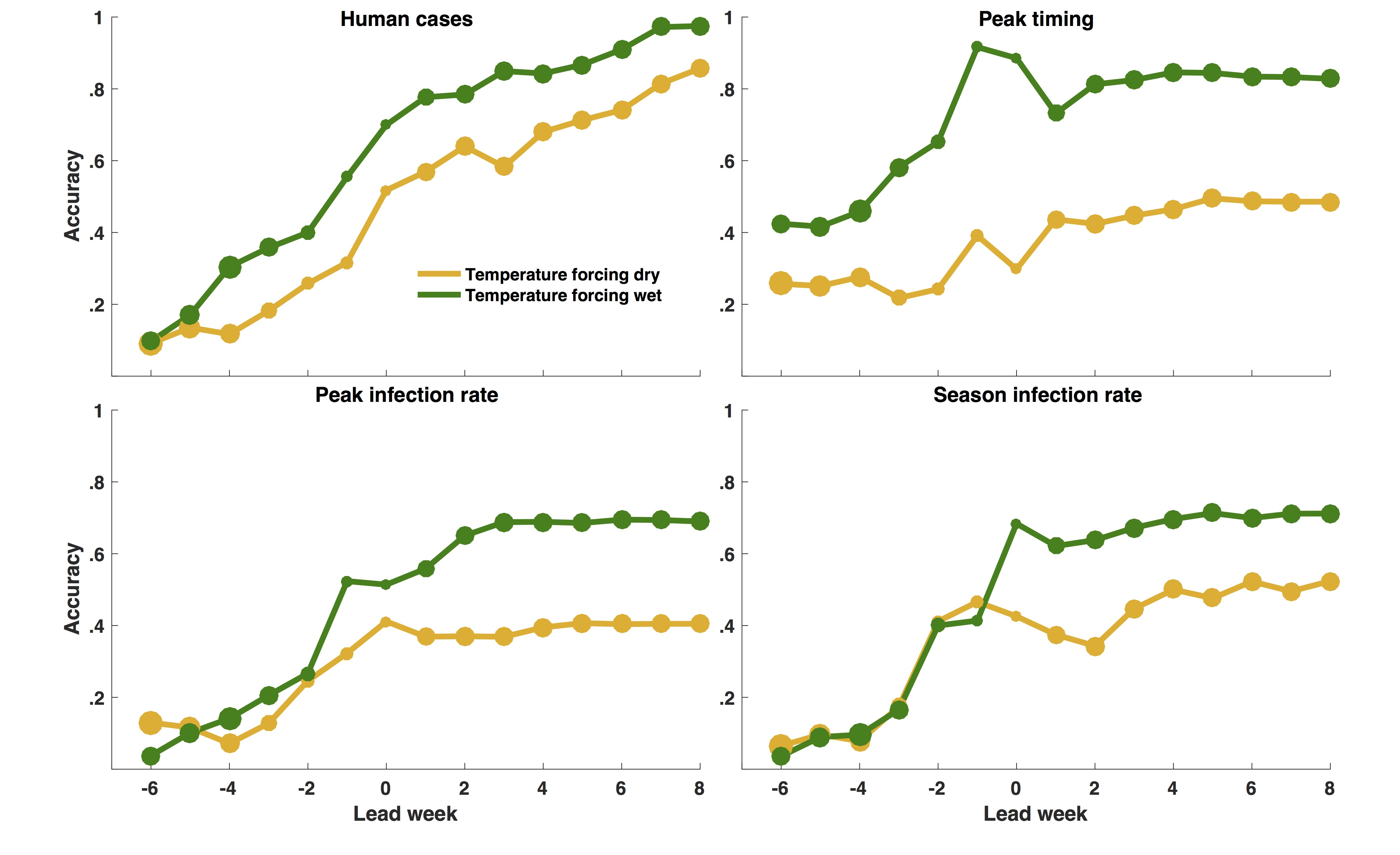

Supplement: S11 Fig — A forecast was deemed accurate if: 1) peak timing was within ±1 week of the observed peak of infectious mosquitoes; 2) peak infection rate was within ±25% of the observed peak infection rate; 3) total infectious mosquitoes were within ±25% of the observed; and 4) human WNV cases were within ±25% or ±1 case of the total number of reported cases, whichever was larger. Note that for all metrics lead week is shown with respect to the week of peak mosquito infection. The size of the dot at each lead week represents the number of forecast generated for that lead week; larger dots indicate more forecasts were generated with a particular predicted lead. Counties that receive more than 10 mm/day of precipitation annually were considered wet (green), Sacramento County CA, Yolo County CA, Cook County IL, Allen County IN, Iberia Parish LA, St Tammany Parish LA, and Suffolk County NY and dry counties received less than 10 mm/day of precipitation annually (yellow), Maricopa County AZ, Orange County CA, Boulder County CO, Weld County CO, and Clark County NV. (TIFF) [file pcbi.1006047.s012.tiff]

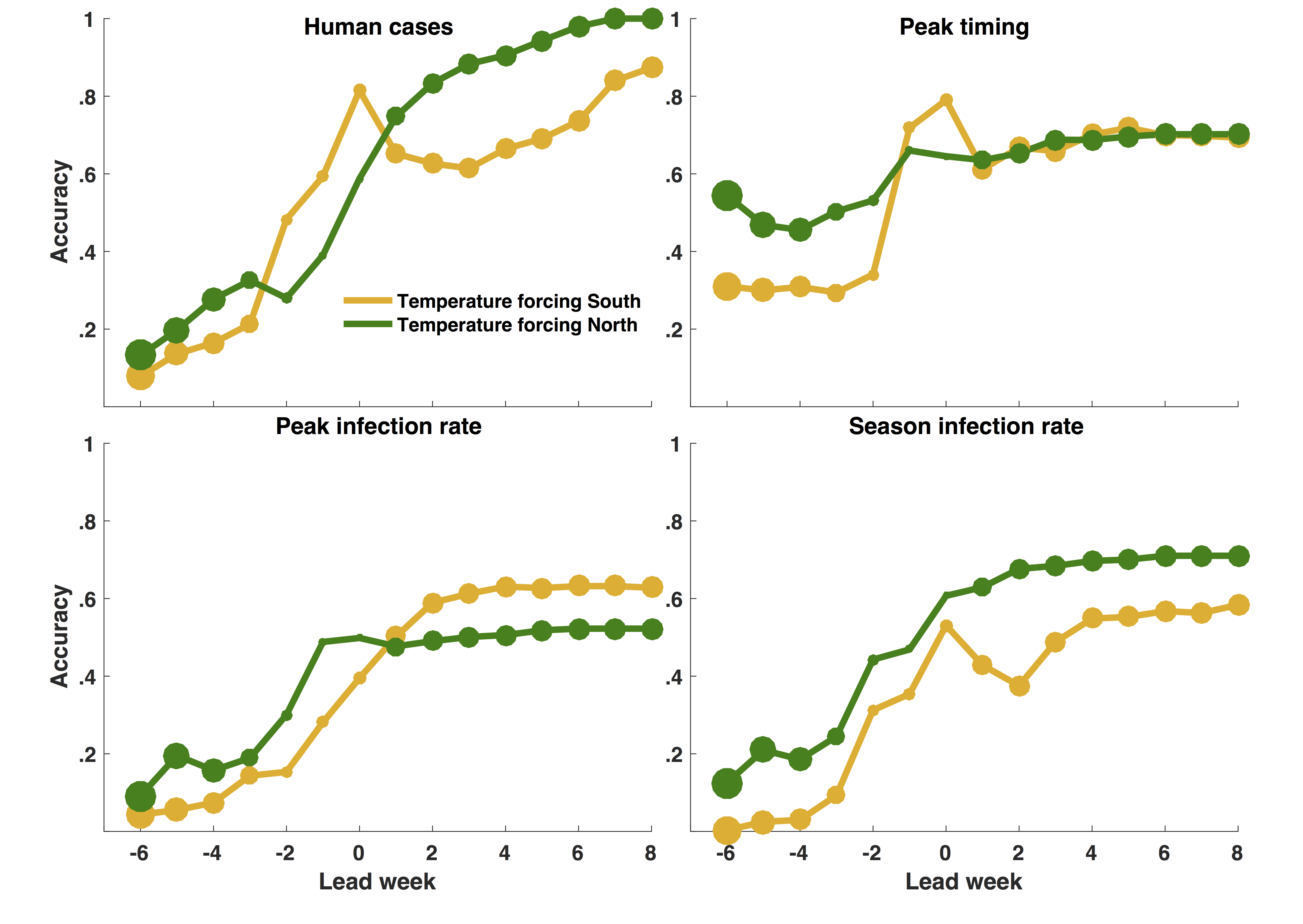

Supplement: S12 Fig — A forecast was deemed accurate if: 1) peak timing was within ±1 week of the observed peak of infectious mosquitoes; 2) peak infection rate was within ±25% of the observed peak infection rate; 3) total infectious mosquitoes were within ±25% of the observed; and 4) human WNV cases were within ±25% or ±1 case of the total number of reported cases, whichever was larger. Note that for all metrics lead week is shown with respect to the week of peak mosquito infection. The size of the dot at each lead week represents the number of forecast generated for that lead week; larger dots indicate more forecasts were generated with a particular predicted lead. Northern counties are north of 40° N latitude (Boulder County CO, Weld County CO, Cook County IL, and Allen County IN, and Suffolk County NY) and southern county are south of 40° N (Maricopa County AZ, Orange County CA, Sacramento County CA, Yolo County CA, Iberia Parish LA, St Tammany Parish LA, and Clark County NV). (TIFF) [file pcbi.1006047.s013.tiff]

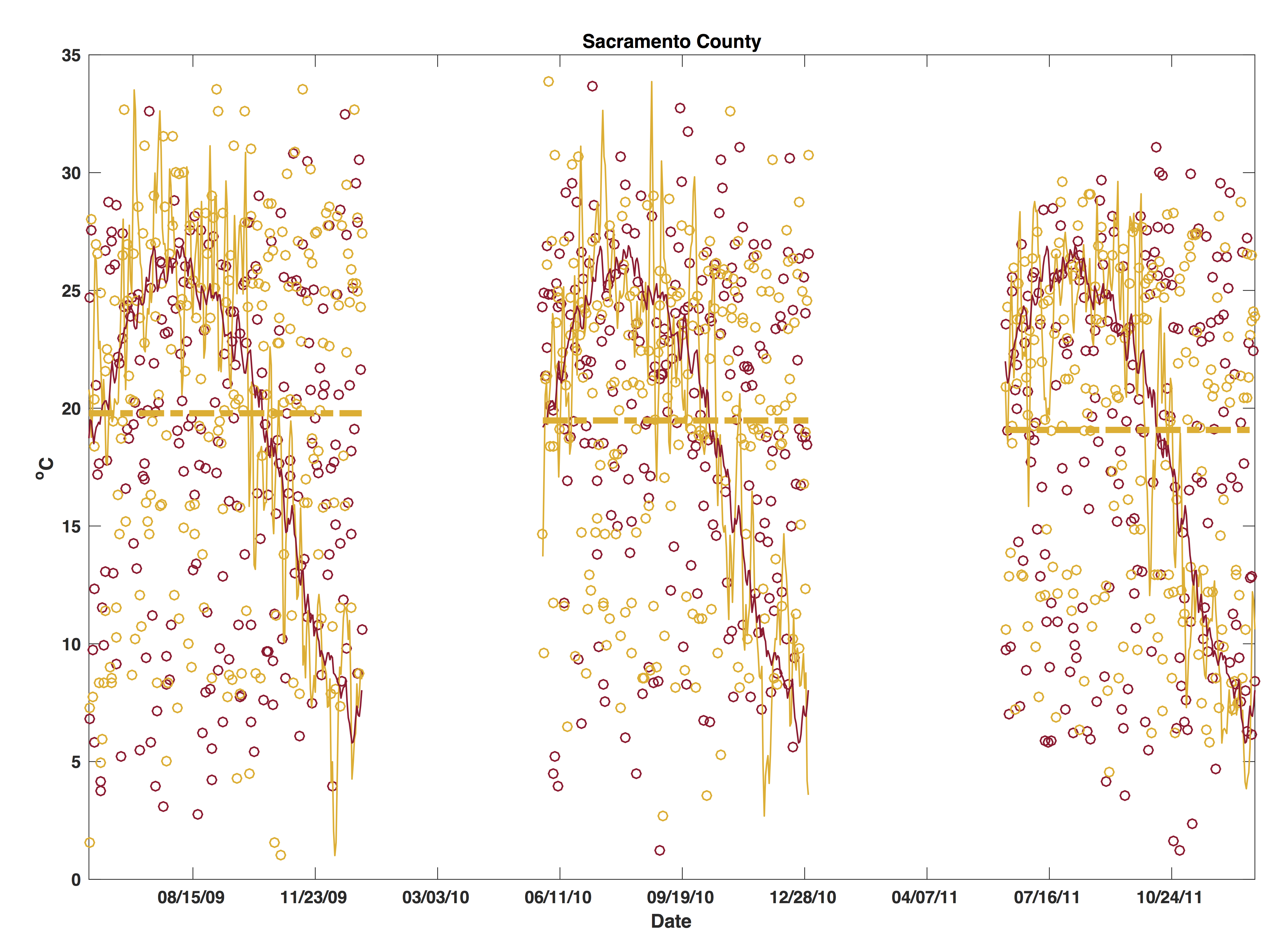

Supplement: S13 Fig — 5 different temperature data streams were used in the model: 1) daily climatology from 1981 to 2000 as described in the main manuscript (red line); 2) observed local daily temperature (yellow line); 3) observed seasonal average temperature, calculated for each outbreak year and location as the mean daily temperature for all weeks the forecast was generated (one temperature value is used for the whole season and fluctuates from season to season, yellow dashed line); 4) permuted observed temperature values: randomly select temperature values from all days in a season (yellow o); and 5) permuted temperature values from the climatology: randomly select temperature values from the day of year which were forecasted in that season (red o). (TIFF) [file pcbi.1006047.s014.tiff]

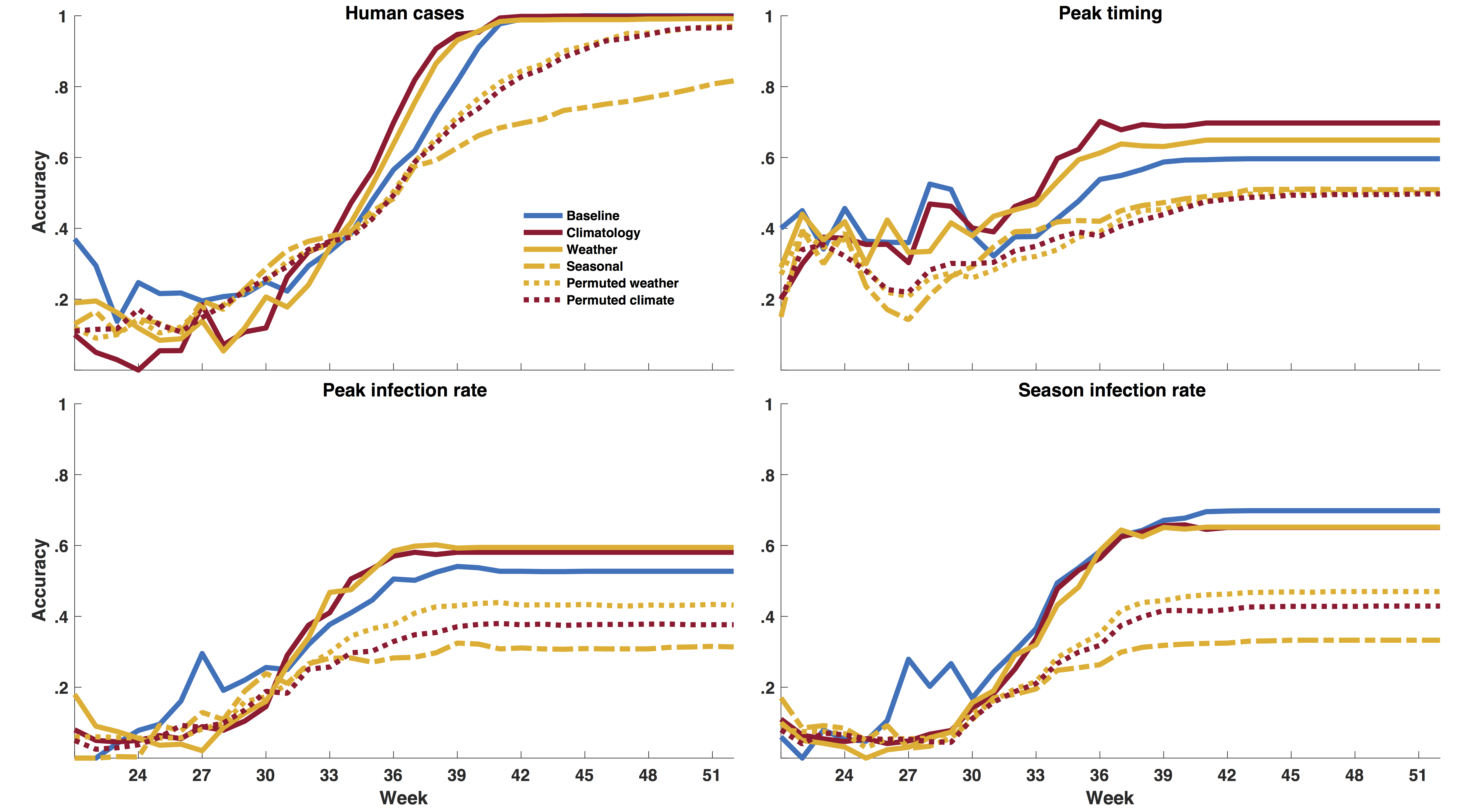

Supplement: S14 Fig — Red represents climatology and yellow represents observed local temperature—daily climatology (red line), observed local daily temperature (yellow line), observed seasonal average temperature (yellow dashed), permuted observed local daily temperature (yellow dot) and permuted climatology (red dot). A forecast was deemed accurate if: 1) peak timing was within ±1 week of the observed peak of infectious mosquitoes; 2) peak infection rate was within ±25% of the observed peak infection rate; 3) total infectious mosquitoes were within ±25% of the observed; and 4) human WNV cases were within ±25% or ±1 case of the total number of reported cases, whichever was larger. Note that for all metrics lead week is shown with respect to the week of peak mosquito infection. The size of the dot at each lead week represents the number of forecast generated for that lead week; larger dots indicate more forecasts were generated with a particular predicted lead. (TIFF) [file pcbi.1006047.s015.tiff]

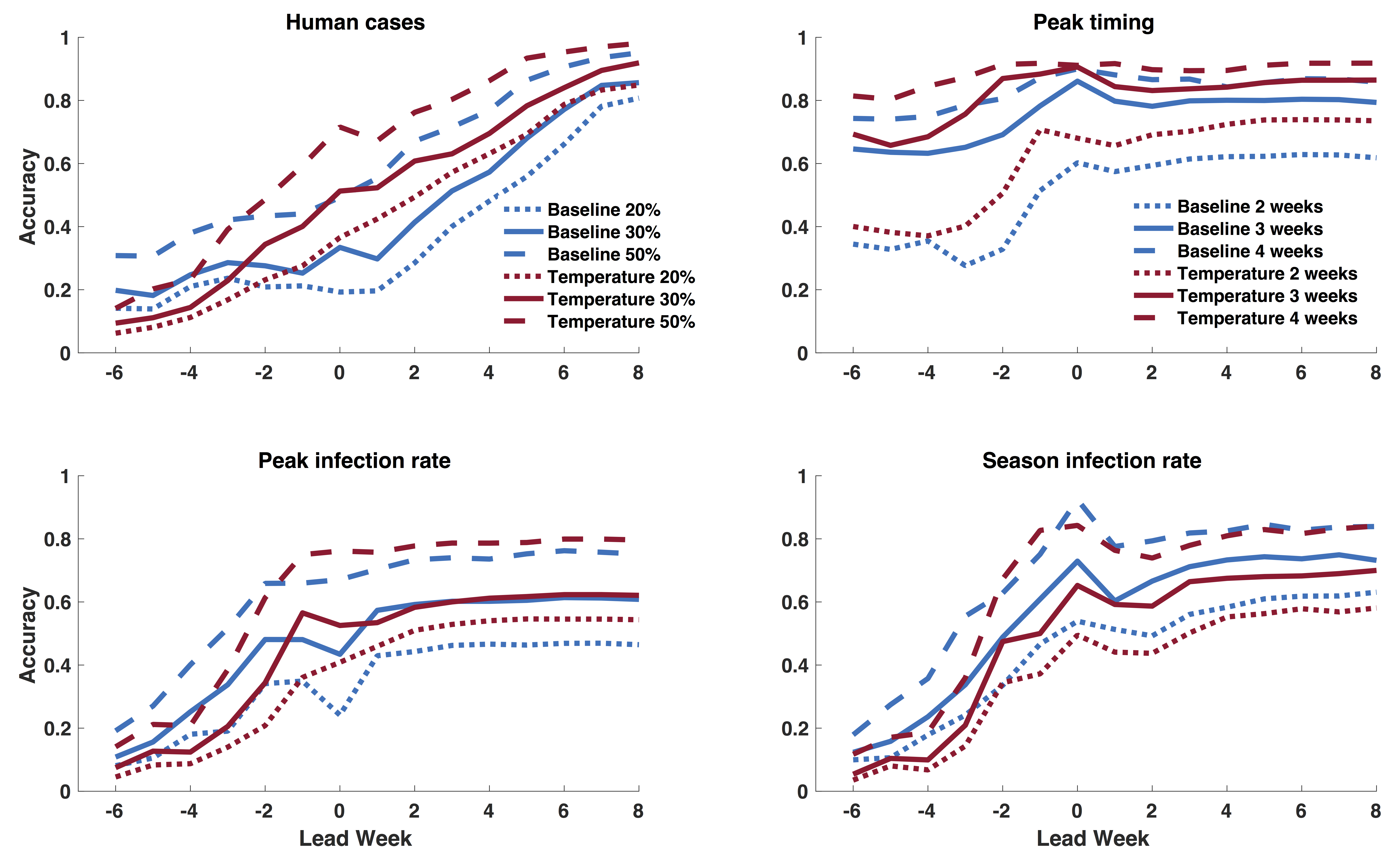

Supplement: S15 Fig — A forecast was deemed accurate if: 1) peak timing was within ±2, ±3, or ±4 weeks of the observed peak of infectious mosquitoes; 2) peak infection rate was within ±20%, ±30% or ±50% of the observed peak infection rate; 3) total infectious mosquitoes were within ±20%, ±30% or ±50% of the observed; and 4) human WNV cases were within ±20%, ±30% or ±50% or ±1 case of the total number of reported cases, whichever was larger. Note that for all metrics lead week is shown with respect to the week of peak mosquito infection. (TIFF) [file pcbi.1006047.s016.tiff]

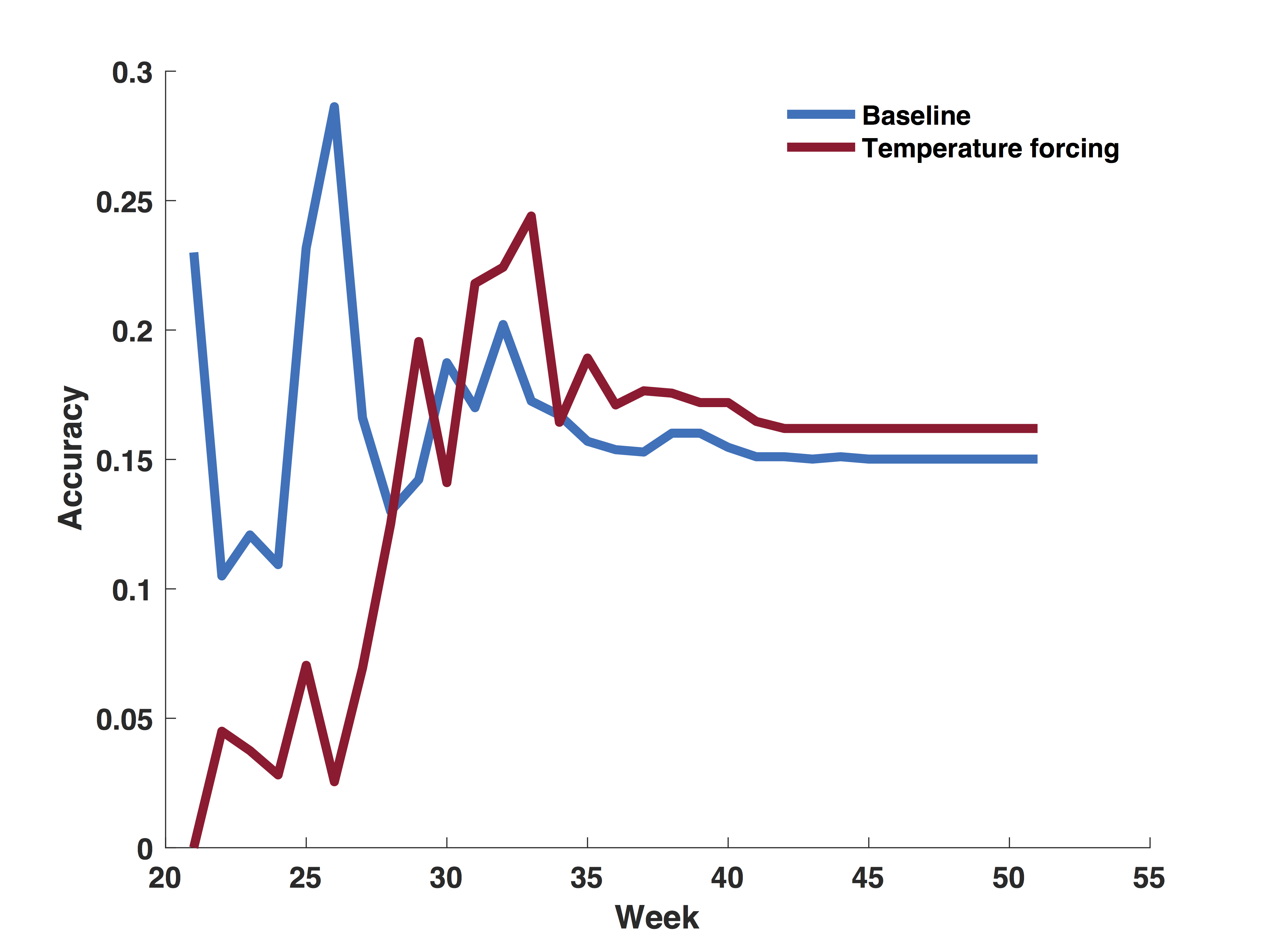

Supplement: S16 Fig — A forecast was deemed accurate if total weekly infectious mosquitoes over a season were within ±25% of the observed. (TIFF) [file pcbi.1006047.s017.tiff]

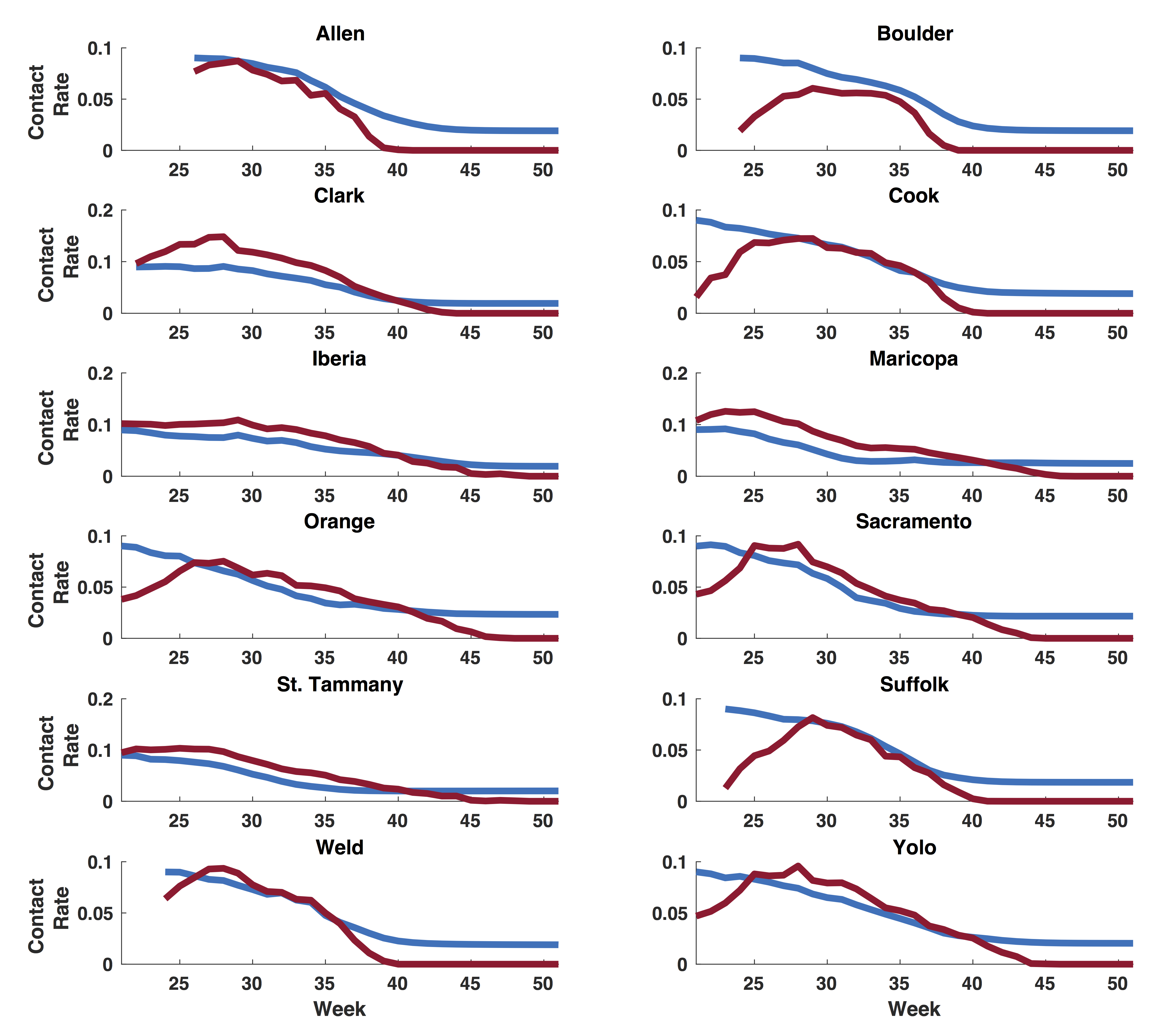

Supplement: S17 Fig — (TIFF) [file pcbi.1006047.s018.tiff]

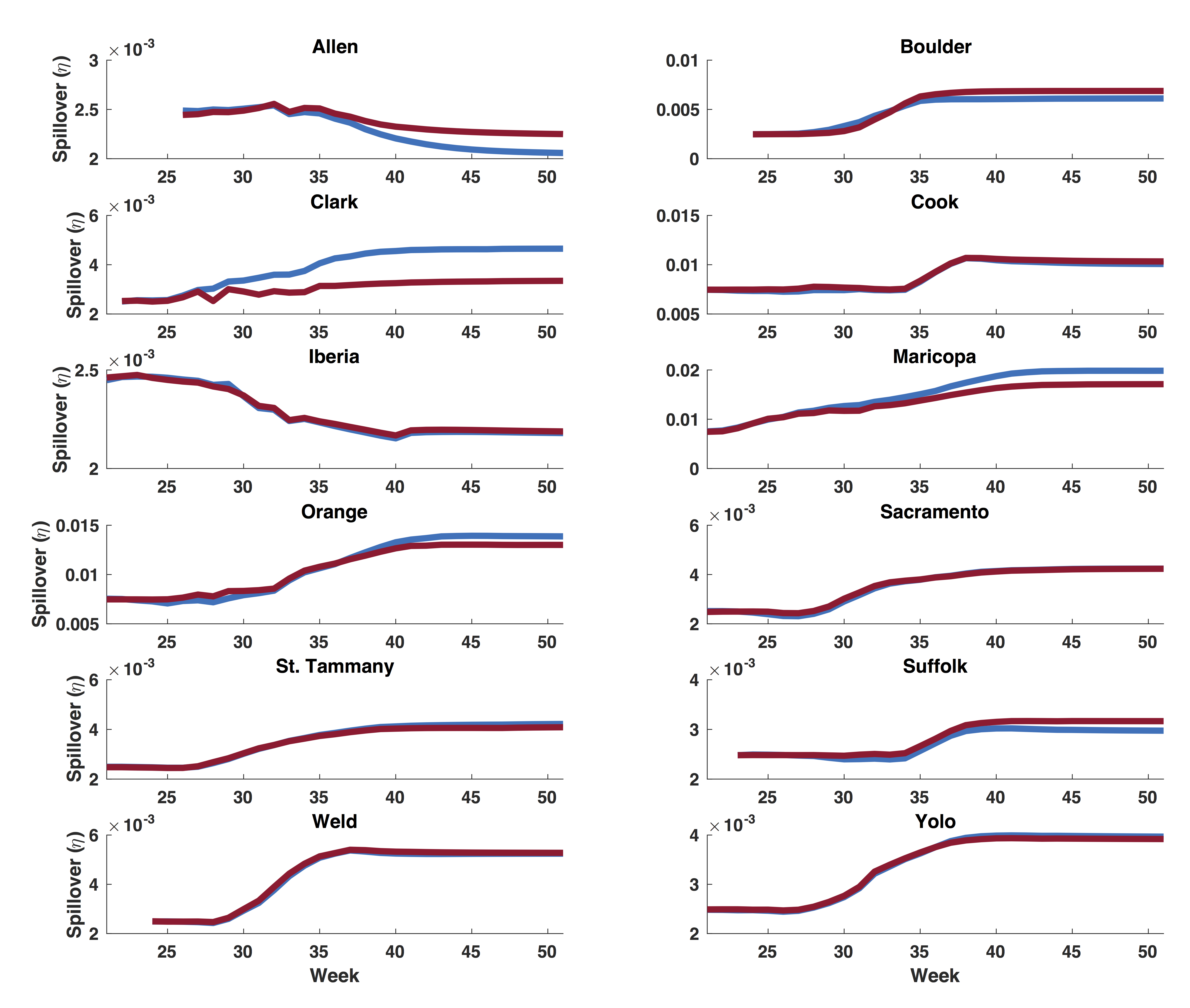

Supplement: S18 Fig — (TIFF) [file pcbi.1006047.s019.tiff]

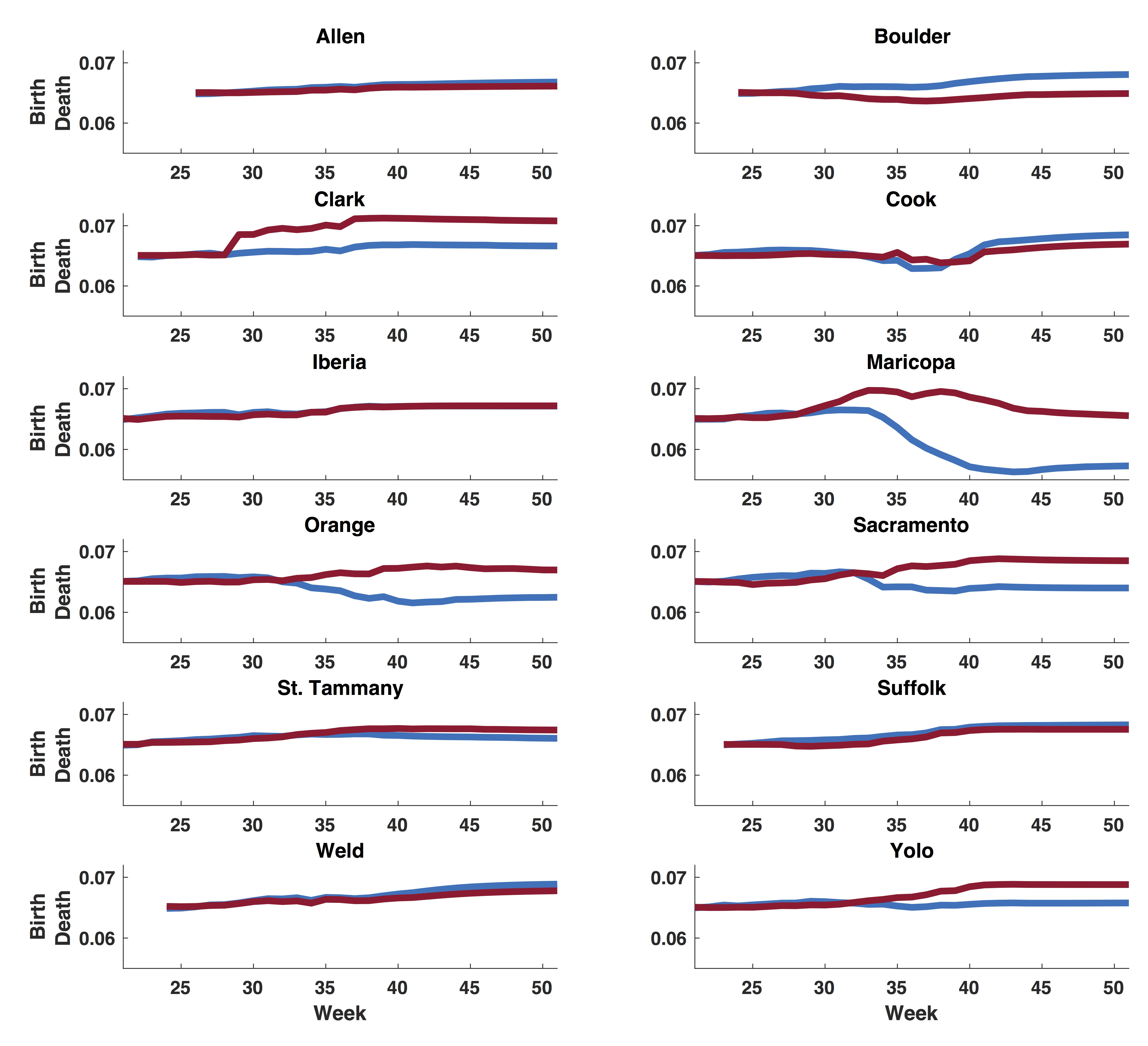

Supplement: S19 Fig — (TIFF) [file pcbi.1006047.s020.tiff]

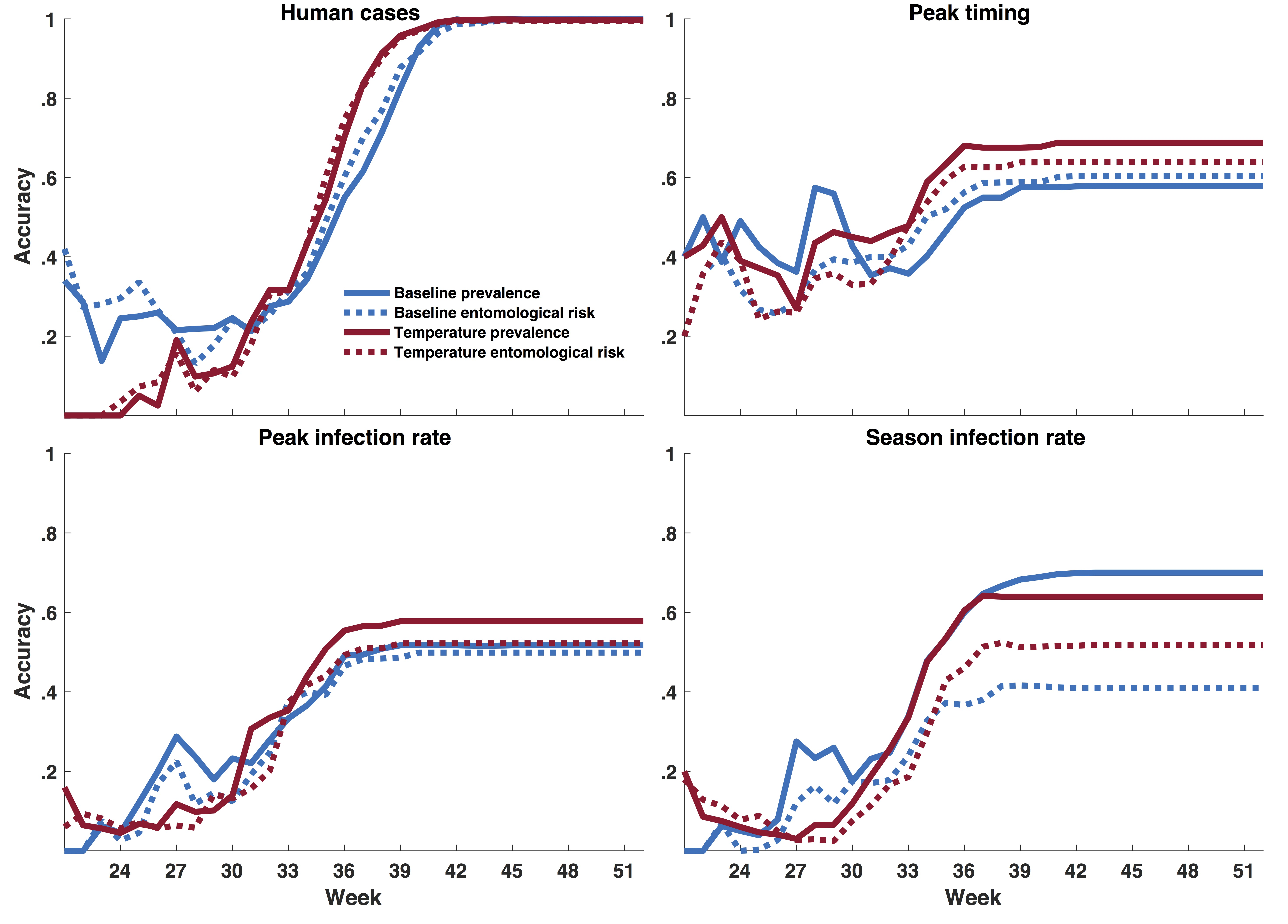

Supplement: S20 Fig — A forecast was deemed accurate if: 1) peak timing was within ±1 week of the observed peak of infectious mosquitoes; 2) peak infection rate was within ±25% of the observed peak infection rate; 3) total infectious mosquitoes were within ±25% of the observed; and 4) human WNV cases were within ±25% or ±1 case of the total number of reported cases, whichever was larger. (TIFF) [file pcbi.1006047.s021.tiff]

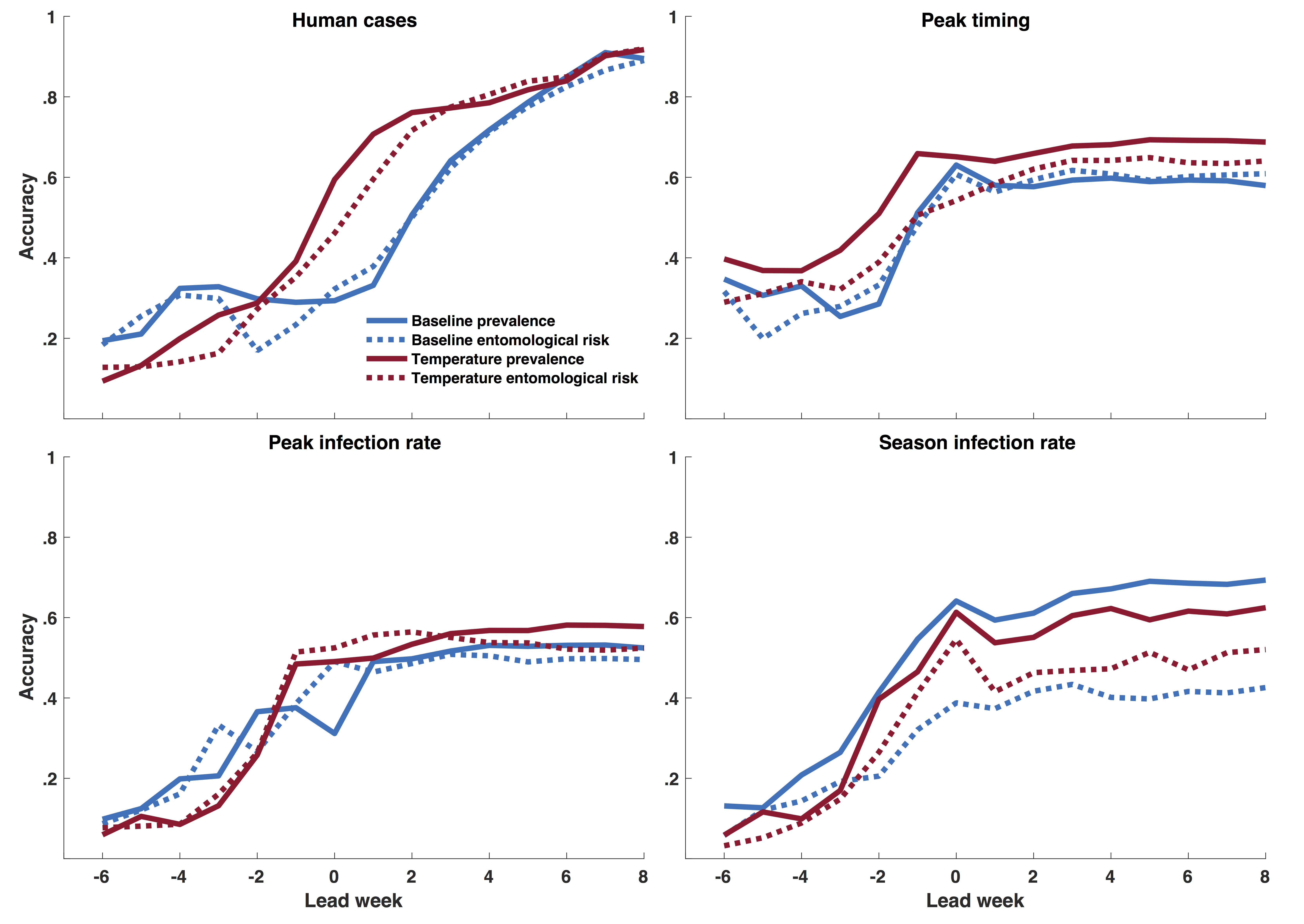

Supplement: S21 Fig — A forecast was deemed accurate if: 1) peak timing was within ±1 week of the observed peak of infectious mosquitoes; 2) peak infection rate was within ±25% of the observed peak infection rate; 3) total infectious mosquitoes were within ±25% of the observed; and 4) human WNV cases were within ±25% or ±1 case of the total number of reported cases, whichever was larger. Note that for all metrics lead week is shown with respect to the week of peak mosquito infection. The size of the dot at each lead week represents the number of forecast generated for that lead week; larger dots indicate more forecasts were generated with a particular predicted lead. (TIFF) [file pcbi.1006047.s022.tiff]

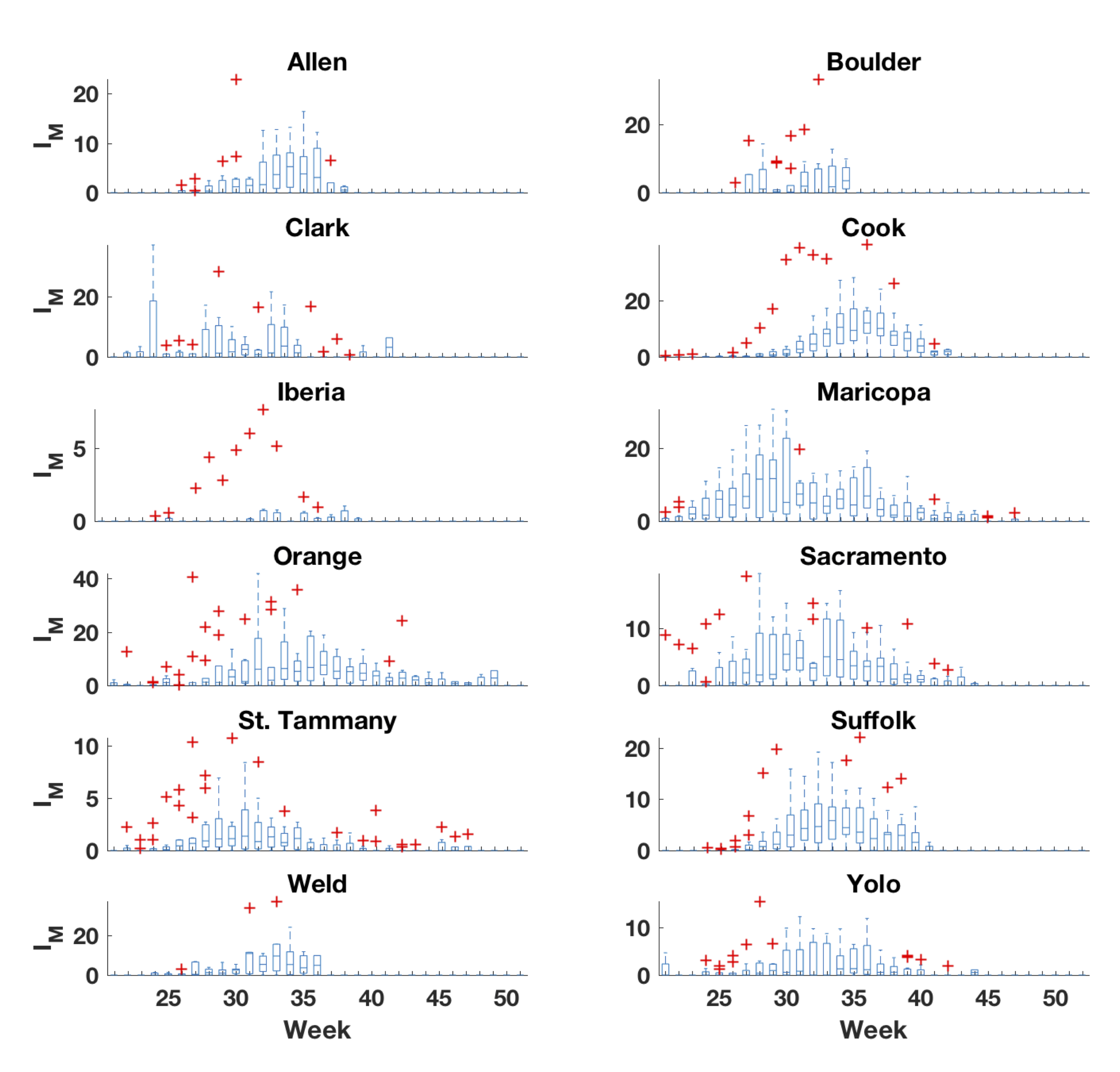

Supplement: S22 Fig — The boxes and whiskers show the median (red horizontal line), 25th and 75th percentiles (box boundaries), the whiskers mark the highest and lowest values within a multiple of 1.5 of the interquartile range of the box boundaries, and outliers are shown as red (+). (TIFF) [file pcbi.1006047.s023.tiff]

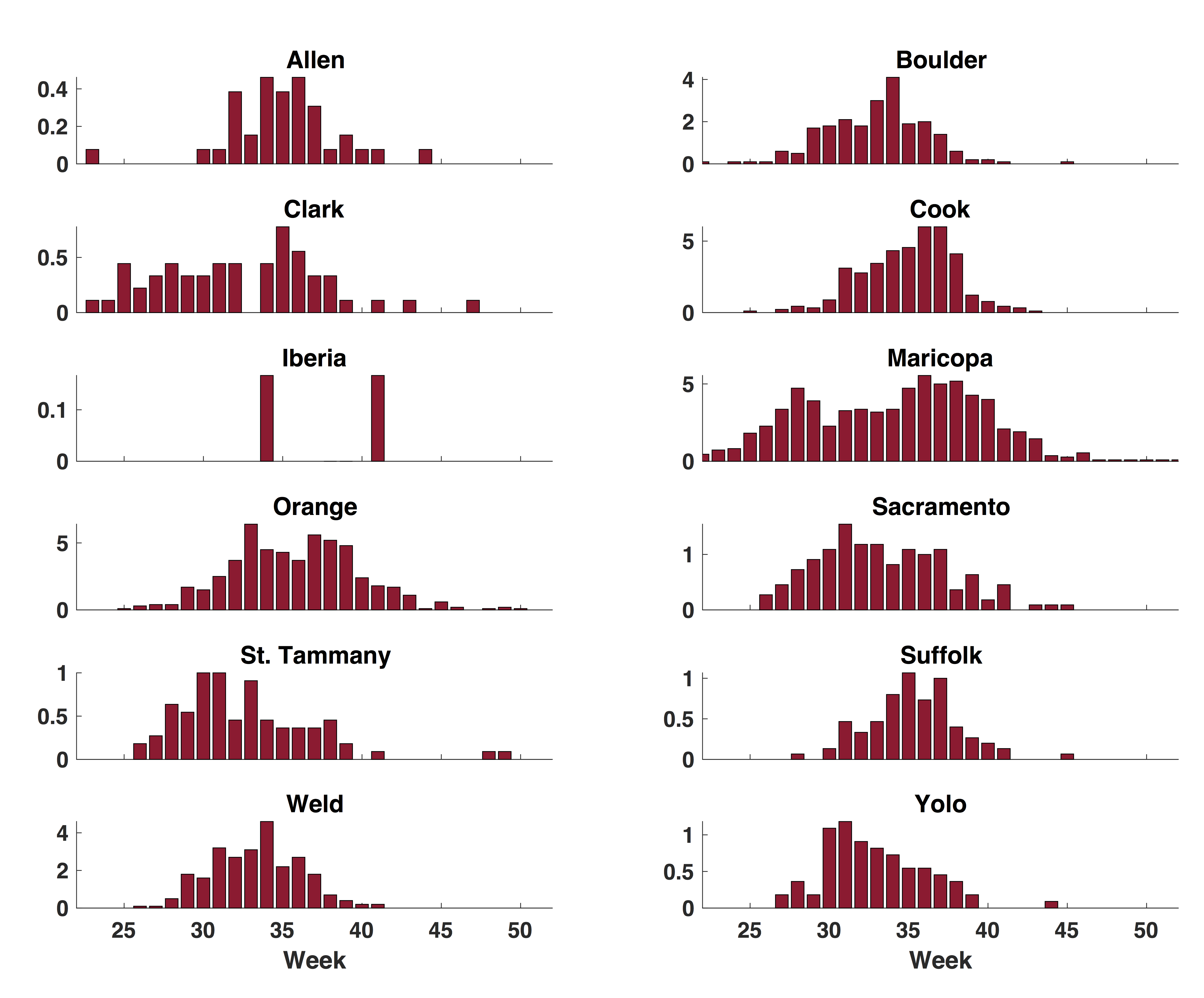

Supplement: S23 Fig — (TIFF) [file pcbi.1006047.s024.tiff]

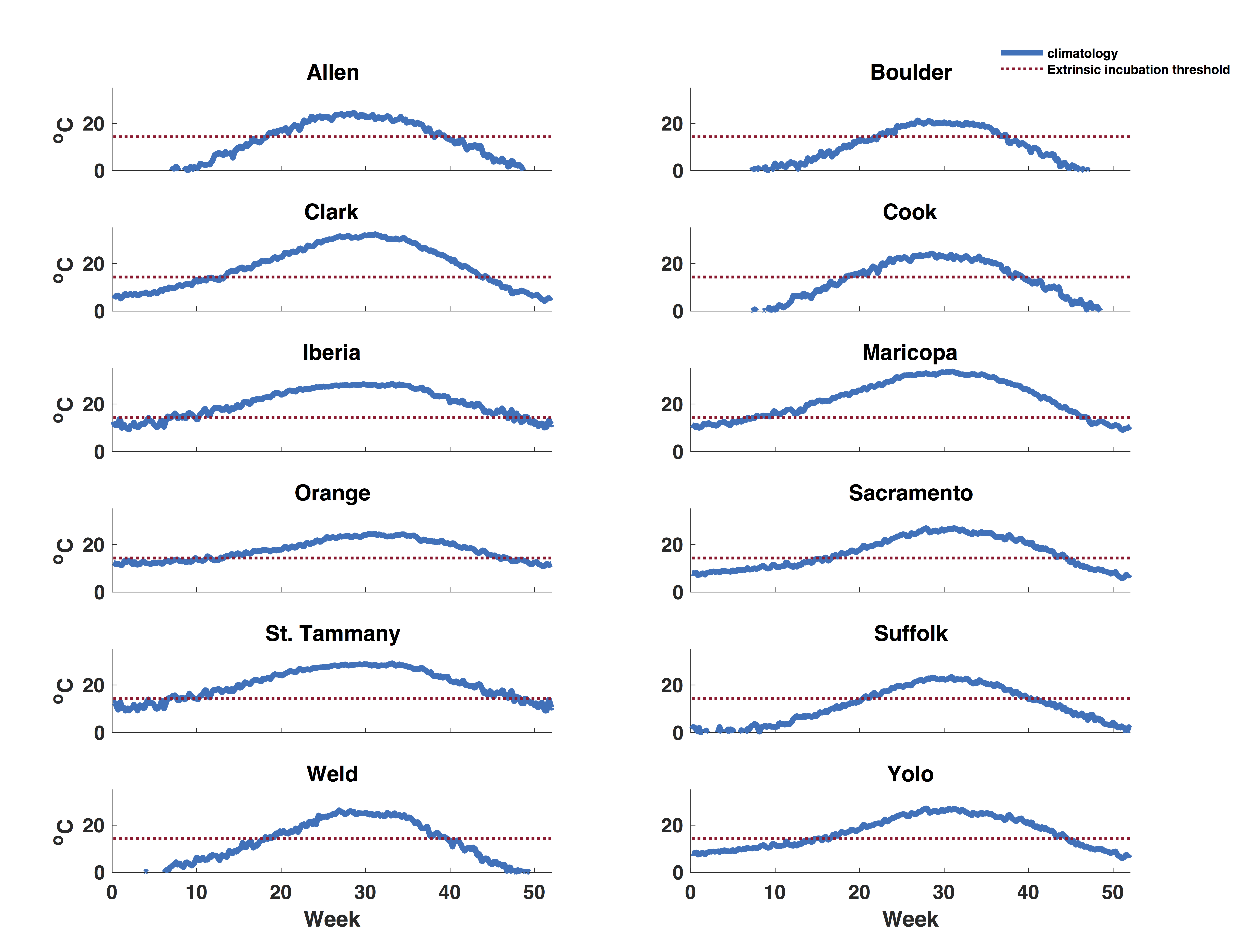

Supplement: S24 Fig — These data are the daily input for the temperature-forced model. The red dotted line is 14.3°C, which represents the minimum threshold for virus development [10, 59]. (JPG) [file pcbi.1006047.s025.jpg]

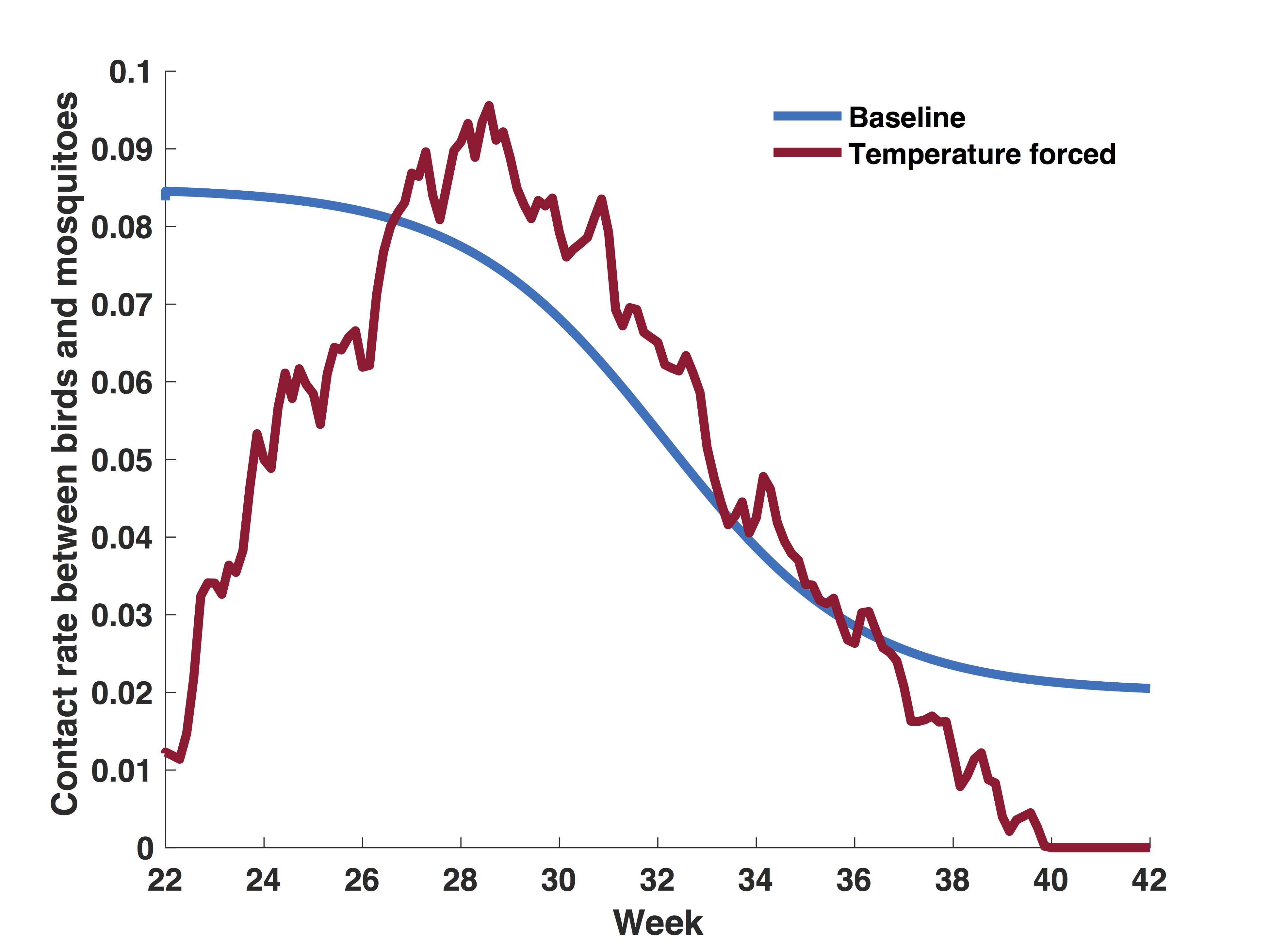

Supplement: S25 Fig — (TIFF) [file pcbi.1006047.s026.tiff]

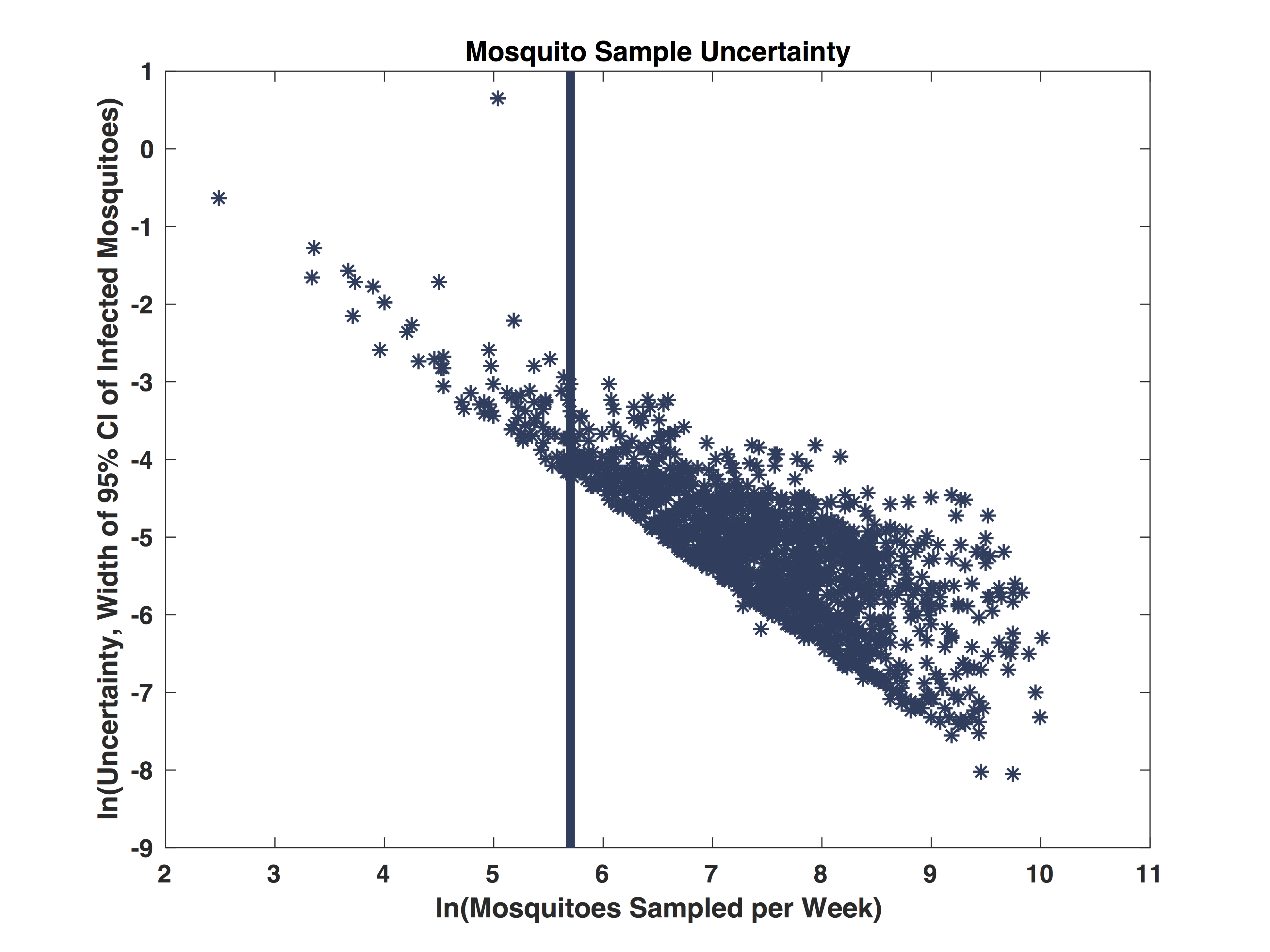

Supplement: S26 Fig — The uncertainty associated with the proportion of mosquitoes infected becomes sensible when 300 or more mosquitoes are sampled. (TIFF) [file pcbi.1006047.s027.tiff]
