# Supplementary material for: Use of temperature to improve West Nile virus forecasts
Source: PLoS Comput Biol. 2018 Mar 9;14(3):e1006047. doi: 10.1371/journal.pcbi.1006047 (PMC5862506; doi:10.1371/journal.pcbi.1006047)
Supplement: S2 Table — Absolute error was calculated and compared for each prediction of total number of human cases over the next week, 2 weeks, 3 weeks and 4 weeks. 1 indicates the temperature-forced model forecasts had statistically significantly less error than the baseline model and -1 indicates the baseline model forecasts had statistically significant less error. (DOCX) [file pcbi.1006047.s029.docx]

**Table S2.** Wilcoxon signed-rank test comparing predicted error between the two modeling approaches. Absolute error was calculated and compared for each prediction of total number of human cases over the next week, 2 weeks, 3 weeks and 4 weeks. 1 indicates the temperature-forced model forecasts had statistically significantly less error than the baseline model and -1 indicates the baseline model forecasts had statistically significant less error.

| Forecast Week | 1 Week | 2 Weeks | 3 Weeks | 4 Weeks |
| --- | --- | --- | --- | --- |
| 21 | 1*** | 1*** | 1*** | 0 |
| 22 | 1*** | 1* | -1* | -1*** |
| 23 | 1** | 0 | -1*** | -1*** |
| 24 | 0 | -1*** | -1*** | -1*** |
| 25 | 0 | -1*** | -1*** | -1*** |
| 26 | 0 | 0 | -1*** | -1*** |
| 27 | 0 | 0 | -1*** | -1*** |
| 28 | 0 | -1*** | -1*** | -1*** |
| 29 | -1*** | -1*** | -1*** | -1*** |
| 30 | -1* | -1*** | 0 | -1* |
| 31 | 0 | 0 | 1* | 1*** |
| 32 | 0 | 1*** | 1*** | 1*** |
| 33 | 1* | 1*** | 1*** | 1*** |
| 34 | 1* | 1*** | 1*** | 1*** |
| 35 | 1*** | 1*** | 1*** | 1*** |
| 36 | 1*** | 1*** | 1*** | 1*** |
| 37 | 1*** | 1*** | 1*** | 1*** |
| 38 | 1*** | 1*** | 1*** | 1*** |
| 39 | 1*** | 1*** | 1*** | 1*** |
| 40 | 1*** | 1*** | 1*** | 1*** |
| 41 | 1*** | 1*** | 1*** | 1*** |
| 42 | 1*** | 1*** | 1*** | 1*** |
| 43 | 1*** | 1*** | 1*** | 1*** |
| 44 | 1*** | 1*** | 1*** | 1*** |
| 45 | 1*** | 1*** | 1*** | 1*** |
| 46 | 1*** | 1*** | 1*** | 1*** |
| 47 | 1*** | 1*** | 1*** | 1*** |
| 48 | 1*** | 1*** | 1*** | 1*** |
| 49 | 1*** | 1*** | 1*** | 1*** |
| 50 | 1*** | 1*** | 1*** | 0 |
| 51 | 1*** | 1*** | 0 | 0 |

Asterisks designate differences significant at p<0.05 (*), p<0.01 (**) and p<0.001 (***).
