# Supplementary material for: Use of temperature to improve West Nile virus forecasts
Source: PLoS Comput Biol. 2018 Mar 9;14(3):e1006047. doi: 10.1371/journal.pcbi.1006047 (PMC5862506; doi:10.1371/journal.pcbi.1006047)
Supplement: S4 Table — Absolute error was calculated and compared for each prediction of observed peak of infectious mosquitoes, maximum mosquito infection rate, and the total number of human cases over the entire season, whereas root mean squared error (RMSE) was used to calculate future forecasts of the total number of mosquitoes observed over the season. 1 indicates the daily observed temperature-forced model forecasts had statistically significantly less error than the daily climatology temperature-forced forecasts and -1 indicates the daily climatology temperature-forced forecasts had statistically significant less error. (DOCX) [file pcbi.1006047.s031.docx]

**Table S4.** Wilcoxon signed-rank test comparing predicted error between the using weather and climatology in the temperature-forced model. Absolute error was calculated and compared for each prediction of observed peak of infectious mosquitoes, maximum mosquito infection rate, and the total number of human cases over the entire season, whereas root mean squared error (RMSE) was used to calculate future forecasts of the total number of mosquitoes observed over the season. 1 indicates the daily observed temperature-forced model forecasts had statistically significantly less error than the daily climatology temperature-forced forecasts and -1 indicates the daily climatology temperature-forced forecasts had statistically significant less error.

| Forecast Week | Human Cases | Peak Timing | Peak Magnitude | Season Forecast |
| --- | --- | --- | --- | --- |
| 21 | -1*** | 0 | -1*** | -1*** |
| 22 | -1* | 0 | -1*** | -1*** |
| 23 | 0 | -1* | -1*** | -1*** |
| 24 | -1** | -1** | -1*** | -1*** |
| 25 | 0 | 0 | -1*** | -1*** |
| 26 | -1*** | 1*** | -1*** | -1*** |
| 27 | -1*** | 0 | -1*** | -1*** |
| 28 | -1*** | -1* | -1*** | -1*** |
| 29 | 0 | 1** | -1*** | -1*** |
| 30 | 0 | 0 | -1*** | -1*** |
| 31 | -1*** | 0 | -1*** | -1*** |
| 32 | -1*** | 0 | -1*** | -1*** |
| 33 | -1*** | 0 | -1** | -1*** |
| 34 | -1*** | -1* | -1*** | -1*** |
| 35 | -1*** | -1* | -1* | -1*** |
| 36 | -1*** | -1*** | 0 | -1*** |
| 37 | -1*** | 0 | 0 | -1*** |
| 38 | -1*** | -1*** | 1* | -1*** |
| 39 | -1*** | -1*** | 1* | -1*** |
| 40 | -1*** | 0 | 0 | 0 |
| 41 | -1*** | -1* | 1* | 1* |
| 42 | -1*** | 0 | 1* | 1* |
| 43 | -1*** | 0 | 1* | -1* |
| 44 | -1*** | 0 | 1* | 0 |
| 45 | -1*** | 0 | 1* | 0 |
| 46 | -1*** | 0 | 1* | 0 |
| 47 | -1*** | 0 | 1* | 0 |
| 48 | -1*** | 0 | 1* | -1** |
| 49 | -1*** | 0 | 1* | -1*** |
| 50 | -1*** | 0 | 1* | -1** |
| 51 | -1*** | 0 | 1* | -1** |

Asterisks designate differences significant at p<0.05 (*), p<0.01 (**) and p<0.001 (***).
