# Supplementary material for: Use of temperature to improve West Nile virus forecasts
Source: PLoS Comput Biol. 2018 Mar 9;14(3):e1006047. doi: 10.1371/journal.pcbi.1006047 (PMC5862506; doi:10.1371/journal.pcbi.1006047)
Supplement: S5 Table — (DOCX) [file pcbi.1006047.s032.docx]

Table S5. Overview of the dominant WNV vector in each county along with the trap type used to monitor these mosquitoes.

| County | Primary WNV vector | Trap Type |
| --- | --- | --- |
| Allen | *Cx. pipiens* and *Cx. restuans* | Gravid and light |
| Boulder | *Cx. tarsalis* and *Cx. pipiens* | Light |
| Clark | *Cx. tarsalis* and *Cx. quinquefasciatus* | Gravid, CO2-baited, and sentinel |
| Cook | *Cx. pipiens* and *Cx. restuans* | Gravid and light |
| Iberia | *Cx. quinquefasciatus* | Gravid and light |
| Maricopa | *Cx. tarsalis* and *Cx. quinquefasciatus* | CO2-baited |
| Orange | *Cx. tarsalis* and *Cx. pipiens* | Gravid and CO_2_-baited |
| Sacramento | *Cx. tarsalis* and *Cx. pipiens* | Gravid and CO_2_-baited |
| St. Tammany | *Cx. quinquefasciatus* | Gravid, CO2-baited, and light |
| Suffolk | *Cx. pipiens* and *Cx. restuans* | Gravid and light |
| Weld County | *Cx. tarsalis* and *Cx. pipiens* | Light |
| Yolo | *Cx. tarsalis* and *Cx. pipiens* | Gravid and CO_2_-baited |
