# Supplementary material for: Use of temperature to improve West Nile virus forecasts
Source: PLoS Comput Biol. 2018 Mar 9;14(3):e1006047. doi: 10.1371/journal.pcbi.1006047 (PMC5862506; doi:10.1371/journal.pcbi.1006047)
Supplement: S6 Table — (DOCX) [file pcbi.1006047.s033.docx]

Table S6. Overview of Human WNV Cases, Mosquito Infection Rates, and Mosquito Data

| Year | Human cases | Weeks sampled | Positive weeks | Peak infection rate* | Peak timing^^^ | No. of *Culex* pools tested | No. of WNV-positive *Culex* pools | Average number of pools per week |
| --- | --- | --- | --- | --- | --- | --- | --- | --- |
| Allen County, IN(46) | | | | | | | | |
| 2007 | 1 | 21 | 1 | 1.6 | 11-Aug-07 | 320 | 1 | 15.2 |
| 2008 | 0 | 19 | 6 | 6.5 | 20-Sep-08 | 467 | 19 | 24.6 |
| 2009 | 0 | 14 | 9 | 3.3 | 5-Sep-09 | 579 | 18 | 41.4 |
| 2010 | 2 | 12 | 9 | 13.2 | 28-Aug-10 | 301 | 51 | 25.1 |
| 2011 | 1 | 11 | 8 | 7.5 | 27-Aug-11 | 475 | 43 | 43.2 |
| 2012 | 12 | 12 | 9 | 12.8 | 25-Aug-12 | 231 | 37 | 19.3 |
| 2013 | 1 | 13 | 11 | 16.4 | 7-Sep-13 | 325 | 48 | 25.0 |
| 2014 | 0 | 12 | 5 | 5.5 | 16-Aug-14 | 259 | 8 | 21.6 |
| 2015 | 4 | 12 | 7 | 9.2 | 22-Aug-15 | 167 | 17 | 13.9 |
| 2016 | 2 | 14 | 4 | 6.6 | 20-Aug-16 | 141 | 7 | 10.1 |
| Boulder County, CO(52) | | | | | | | | |
| 2007 | 95 | 15 | 8 | 10.6 | 25-Aug-07 | 359 | 36 | 23.9 |
| 2008 | 13 | 13 | 3 | 3.5 | 23-Aug-08 | 370 | 5 | 28.5 |
| 2009 | 12 | 13 | 3 | 2.2 | 29-Aug-09 | 460 | 6 | 35.4 |
| 2010 | 6 | 12 | 1 | 0.7 | 24-Jul-10 | 283 | 1 | 23.6 |
| 2011 | 2 | 11 | 3 | 9.9 | 27-Aug-11 | 309 | 14 | 28.1 |
| 2012 | 1 | 12 | 5 | 10.8 | 21-Jul-12 | 191 | 15 | 15.9 |
| 2013 | 52 | 14 | 13 | 33.4 | 17-Aug-13 | 281 | 100 | 20.1 |
| 2014 | 11 | 12 | 8 | 16.6 | 2-Aug-14 | 245 | 34 | 20.4 |
| 2015 | 11 | 12 | 5 | 7.2 | 1-Aug-15 | 200 | 18 | 16.7 |
| 2016 | 23 | 11 | 6 | 12.8 | 20-Aug-16 | 176 | 22 | 16.0 |
| Clark County, NV(45) | | | | | | | | |
| 2008 | 13 | 23 | 5 | 10.1 | 26-Jul-08 | 322 | 16 | 14.0 |
| 2009 | 12 | 32 | 7 | 6.7 | 1-Aug-09 | 497 | 12 | 15.5 |
| 2011 | 11 | 27 | 3 | 2.5 | 6-Aug-11 | 653 | 7 | 24.2 |
| 2012 | 8 | 21 | 4 | 6.4 | 20-Oct-12 | 373 | 6 | 17.8 |
| 2013 | 9 | 24 | 7 | 10.3 | 24-Aug-13 | 460 | 15 | 19.2 |
| 2014 | 2 | 23 | 9 | 21.7 | 16-Aug-14 | 893 | 61 | 38.8 |
| Cook, IL(47, 48, 50) | | | | | | | | |
| 2007 | 33 | 24 | 17 | 15.3 | 25-Aug-07 | 6164 | 749 | 256.8 |
| 2008 | 9 | 23 | 15 | 10.7 | 6-Sep-08 | 4275 | 348 | 185.9 |
| 2009 | 1 | 24 | 16 | 8.4 | 5-Sep-09 | 4737 | 145 | 197.4 |
| 2010 | 30 | 22 | 16 | 39.7 | 4-Sep-10 | 5960 | 1130 | 270.9 |
| 2011 | 22 | 22 | 15 | 14.3 | 10-Sep-11 | 4187 | 333 | 190.3 |
| 2012 | 174 | 23 | 19 | 38.8 | 4-Aug-12 | 5087 | 1528 | 221.2 |
| 2013 | 59 | 24 | 18 | 20.2 | 31-Aug-13 | 6666 | 1242 | 277.8 |
| 2014 | 26 | 23 | 16 | 15.9 | 6-Sep-14 | 6103 | 632 | 265.3 |
| Iberia Parish, LA(49) | | | | | | | | |
| 2012 | 2 | 29 | 14 | 7.7 | 18-Aug-12 | 2292 | 161 | 79.0 |
| 2013 | 0 | 32 | 9 | 0.9 | 28-Sep-13 | 2064 | 15 | 64.5 |
| 2014 | 0 | 32 | 1 | 0.5 | 16-Aug-14 | 1862 | 2 | 58.2 |
| 2016 | 0 | 32 | 2 | 0.2 | 3-Sep-16 | 2154 | 2 | 67.3 |
| Maricopa County, AZ(51) | | | | | | | | |
| 2006 | 77 | 52 | 14 | 14.9 | 2-Sep-06 | 3065 | 124 | 58.9 |
| 2007 | 68 | 52 | 20 | 18.6 | 8-Sep-07 | 5208 | 121 | 100.2 |
| 2008 | 91 | 51 | 18 | 15.6 | 6-Sep-08 | 7005 | 100 | 137.4 |
| 2009 | 19 | 51 | 17 | 17.9 | 18-Jul-09 | 5135 | 59 | 100.7 |
| 2010 | 116 | 51 | 20 | 30.7 | 17-Jul-10 | 4138 | 160 | 81.1 |
| 2011 | 45 | 51 | 18 | 22.4 | 9-Jul-11 | 4851 | 93 | 95.1 |
| 2012 | 88 | 52 | 26 | 29.4 | 28-Jul-12 | 5592 | 166 | 107.5 |
| 2013 | 52 | 51 | 26 | 24.2 | 6-Jul-13 | 6436 | 148 | 126.2 |
| 2014 | 94 | 50 | 25 | 30.3 | 26-Jul-14 | 9106 | 220 | 182.1 |
| 2015 | 59 | 50 | 23 | 14.6 | 20-Jun-15 | 9709 | 97 | 194.2 |
| 2016 | 60 | 51 | 27 | 11.5 | 9-Jul-16 | 10515 | 112 | 206.2 |
| Orange County, CA(44) | | | | | | | | |
| 2007 | 9 | 32 | 9 | 20.2 | 8-Sep-07 | 509 | 24 | 15.9 |
| 2008 | 71 | 44 | 25 | 40.4 | 5-Jul-08 | 1192 | 305 | 27.1 |
| 2009 | 4 | 40 | 10 | 5.7 | 29-Aug-09 | 1098 | 15 | 27.5 |
| 2010 | 1 | 37 | 11 | 3.2 | 24-Jul-10 | 1193 | 18 | 32.2 |
| 2011 | 10 | 34 | 15 | 8.7 | 6-Aug-11 | 1055 | 90 | 31.0 |
| 2012 | 42 | 43 | 19 | 13.4 | 6-Oct-12 | 1179 | 69 | 27.4 |
| 2013 | 11 | 38 | 12 | 7.7 | 14-Sep-13 | 1512 | 46 | 39.8 |
| 2014 | 263 | 37 | 23 | 41.8 | 9-Aug-14 | 2310 | 497 | 62.4 |
| 2015 | 92 | 49 | 27 | 18.8 | 12-Sep-15 | 4717 | 571 | 96.3 |
| Sacramento County, CA(44) | | | | | | | | |
| 2006 | 15 | 38 | 14 | 6.3 | 29-Jul-06 | 1991 | 35 | 52.4 |
| 2007 | 25 | 39 | 13 | 5.5 | 28-Jul-07 | 3889 | 129 | 99.7 |
| 2008 | 13 | 33 | 17 | 5.0 | 16-Aug-08 | 4988 | 202 | 151.2 |
| 2009 | 0 | 29 | 11 | 3.3 | 5-Sep-09 | 2732 | 35 | 94.2 |
| 2010 | 12 | 38 | 17 | 9.7 | 10-Jul-10 | 4223 | 201 | 111.1 |
| 2011 | 4 | 33 | 15 | 16.7 | 20-Aug-11 | 4689 | 370 | 142.1 |
| 2012 | 29 | 34 | 20 | 19.7 | 14-Jul-12 | 4591 | 484 | 135.0 |
| 2013 | 11 | 27 | 20 | 13.8 | 24-Aug-13 | 4485 | 383 | 166.1 |
| 2014 | 10 | 28 | 20 | 14.5 | 9-Aug-14 | 5231 | 487 | 186.8 |
| 2015 | 4 | 28 | 18 | 5.8 | 22-Aug-15 | 4568 | 164 | 163.1 |
| St. Tammany Parish, LA(33) | | | | | | | | |
| 2006 | 33 | 39 | 17 | 10.7 | 29-Jul-06 | 1084 | 80 | 27.8 |
| 2007 | 1 | 49 | 23 | 3.8 | 13-Oct-07 | 2564 | 47 | 52.3 |
| 2008 | 8 | 50 | 30 | 3.2 | 2-Aug-08 | 2978 | 88 | 59.6 |
| 2009 | 6 | 46 | 18 | 4.3 | 18-Jul-09 | 2575 | 49 | 56.0 |
| 2010 | 1 | 29 | 8 | 2.5 | 28-Aug-10 | 1067 | 13 | 36.8 |
| 2011 | 2 | 41 | 10 | 1.0 | 9-Jul-11 | 1794 | 17 | 43.8 |
| 2012 | 21 | 44 | 17 | 10.3 | 7-Jul-12 | 2214 | 145 | 50.3 |
| 2013 | 3 | 47 | 10 | 2.4 | 3-Aug-13 | 1852 | 26 | 39.4 |
| 2014 | 4 | 51 | 12 | 8.4 | 2-Aug-14 | 1720 | 36 | 33.7 |
| 2015 | 2 | 50 | 11 | 5.0 | 8-Aug-15 | 2380 | 65 | 47.6 |
| 2016 | 1 | 45 | 10 | 2.7 | 27-Aug-16 | 2614 | 19 | 58.1 |
| Suffolk County, NY(32) | | | | | | | | |
| 2001 | 1 | 18 | 10 | 9.0 | 11-Aug-01 | 721 | 48 | 40.1 |
| 2002 | 8 | 18 | 9 | 9.9 | 31-Aug-02 | 762 | 30 | 42.3 |
| 2003 | 10 | 19 | 8 | 4.1 | 30-Aug-03 | 1088 | 33 | 57.3 |
| 2004 | 0 | 19 | 5 | 3.3 | 18-Sep-04 | 613 | 7 | 32.3 |
| 2005 | 9 | 19 | 12 | 22.0 | 3-Sep-05 | 1051 | 69 | 55.3 |
| 2006 | 2 | 19 | 10 | 10.1 | 26-Aug-06 | 858 | 54 | 45.2 |
| 2007 | 0 | 18 | 8 | 4.0 | 11-Aug-07 | 432 | 12 | 24.0 |
| 2008 | 9 | 19 | 12 | 7.9 | 23-Aug-08 | 644 | 41 | 33.9 |
| 2009 | 1 | 18 | 6 | 7.8 | 19-Sep-09 | 775 | 15 | 43.1 |
| 2010 | 24 | 19 | 14 | 19.0 | 14-Aug-10 | 1473 | 285 | 77.5 |
| 2011 | 4 | 20 | 11 | 10.5 | 13-Aug-11 | 1338 | 79 | 66.9 |
| 2012 | 14 | 17 | 12 | 19.8 | 28-Jul-12 | 912 | 204 | 53.6 |
| 2013 | 4 | 21 | 14 | 8.6 | 3-Aug-13 | 1332 | 178 | 63.4 |
| 2014 | 1 | 18 | 14 | 11.5 | 16-Aug-14 | 1347 | 186 | 74.8 |
| 2015 | 5 | 19 | 16 | 12.1 | 5-Sep-15 | 1350 | 197 | 71.1 |
| Weld County(52) | | | | | | | | |
| 2011 | 0 | 12 | 5 | 5.3 | 27-Aug-11 | 664 | 12 | 55.3 |
| 2012 | 5 | 12 | 7 | 37.6 | 25-Aug-12 | 315 | 36 | 26.3 |
| 2013 | 43 | 12 | 10 | 15.4 | 24-Aug-13 | 462 | 114 | 38.5 |
| 2014 | 25 | 12 | 11 | 24.3 | 30-Aug-14 | 538 | 111 | 44.8 |
| 2015 | 10 | 12 | 8 | 10.9 | 8-Aug-15 | 373 | 35 | 31.1 |
| 2016 | 27 | 10 | 7 | 11.4 | 6-Aug-16 | 323 | 62 | 32.3 |
| Yolo County, CA(44) | | | | | | | | |
| 2006 | 27 | 35 | 9 | 3.2 | 26-Aug-06 | 1209 | 30 | 34.5 |
| 2007 | 2 | 39 | 5 | 0.8 | 4-Aug-07 | 1664 | 8 | 42.7 |
| 2008 | 1 | 32 | 7 | 1.3 | 30-Aug-08 | 1719 | 11 | 53.7 |
| 2009 | 2 | 28 | 5 | 4.8 | 29-Aug-09 | 1027 | 16 | 36.7 |
| 2010 | 0 | 38 | 6 | 2.1 | 2-Oct-10 | 1245 | 10 | 32.8 |
| 2011 | 0 | 33 | 4 | 3.3 | 1-Oct-11 | 1090 | 7 | 33.0 |
| 2012 | 10 | 32 | 13 | 11.8 | 8-Sep-12 | 1631 | 154 | 51.0 |
| 2013 | 6 | 25 | 16 | 15.4 | 13-Jul-13 | 1994 | 245 | 79.8 |
| 2014 | 15 | 26 | 16 | 12.2 | 2-Aug-14 | 2185 | 220 | 84.0 |
| 2015 | 8 | 26 | 17 | 7.5 | 8-Aug-15 | 2426 | 172 | 93.3 |

*Per 1,000 mosquitoes estimated using the MLE

^ Last day of the week
