# Supplementary material for: Use of temperature to improve West Nile virus forecasts
Source: PLoS Comput Biol. 2018 Mar 9;14(3):e1006047. doi: 10.1371/journal.pcbi.1006047 (PMC5862506; doi:10.1371/journal.pcbi.1006047)
Supplement: S7 Table — (DOCX) [file pcbi.1006047.s034.docx]

**Table S7.** Annual average precipitation, climatology and latitude for each county evaluated.

| County | Annual Precipitation (mm/day) | Weeks Greater than 14.3 | Latitude |
| --- | --- | --- | --- |
| Allen | 29.7 | 21 | 41.1°N |
| Boulder | 10.0 | 15 | 40.2°N |
| Clark | 3.7 | 31 | 36.1°N |
| Cook | 28.5 | 21 | 41.7°N |
| Iberia | 45.2 | 41 | 29.7°N |
| Maricopa | 9.1 | 39 | 33.3°N |
| Orange | 8.6 | 33 | 33.7°N |
| Sacramento | 18.4 | 28 | 38.5°N |
| St. Tammany | 47.2 | 41 | 30.4°N |
| Suffolk | 34.2 | 20 | 41°N |
| Weld County | 12.5 | 21 | 40.4°N |
| Yolo | 18.4 | 28 | 38.8°N |
