# Supplementary material for: Use of temperature to improve West Nile virus forecasts
Source: PLoS Comput Biol. 2018 Mar 9;14(3):e1006047. doi: 10.1371/journal.pcbi.1006047 (PMC5862506; doi:10.1371/journal.pcbi.1006047)
Supplement: S8 Table — (DOCX) [file pcbi.1006047.s035.docx]

**Table S8.** Correlation between the seasonal sum of weekly observed infected mosquito (prevalence) and the total number of human WNV cases over the season or seasonal sum of weekly observed infected mosquito (Entomological risk, prevalence times weekly average number of mosquitoes per trap night) and the total number of human WNV cases over the season.

| Annual Correlation | | |
| --- | --- | --- |
| County | Prevalence | Entomological risk |
| Allen | 0.62* | 0.56* |
| Boulder | 0.29 | 0.69* |
| Clark | 0.19 | -0.08 |
| Cook | 0.82* | 0.78* |
| Iberia Parish | 0.99* | 0.99* |
| Maricopa | 0.61* | 0.54 |
| St. Tammany Parish | 0.72* | 0.98* |
| Suffolk | 0.75* | 0.44 |
| Weld | -0.08 | -0.03 |

* P is significant at 0.05
